# Selective inactivation of hypomethylating agents by SAMHD1 provides a rationale for therapeutic stratification in AML

**DOI:** 10.1038/s41467-019-11413-4

**Published:** 2019-08-02

**Authors:** Thomas Oellerich, Constanze Schneider, Dominique Thomas, Kirsten M. Knecht, Olga Buzovetsky, Lars Kaderali, Christoph Schliemann, Hanibal Bohnenberger, Linus Angenendt, Wolfgang Hartmann, Eva Wardelmann, Tamara Rothenburger, Sebastian Mohr, Sebastian Scheich, Federico Comoglio, Anne Wilke, Philipp Ströbel, Hubert Serve, Martin Michaelis, Nerea Ferreirós, Gerd Geisslinger, Yong Xiong, Oliver T. Keppler, Jindrich Cinatl

**Affiliations:** 1 Department of Medicine II, Hematology/Oncology, Goethe University of Frankfurt, Frankfurt, 60590 Germany; 2 German Cancer Consortium/German Cancer Research Center, Heidelberg, 69120 Germany; 3 Frankfurt Cancer Institute, Goethe University Frankfurt, Frankfurt, 60596 Germany; 4 Institute of Medical Virology, University of Frankfurt, Frankfurt, 60590 Germany; 5 pharmazentrum frankfurt/ZAFES, Institute of Clinical Pharmacology, Goethe University of Frankfurt, Frankfurt, 60590 Germany; 6 Department of Molecular Biophysics and Biochemistry, Yale University, New Haven, CT 06520 USA; 7 Institute of Bioinformatics, University Medicine Greifswald, Greifswald, 17475 Germany; 8 Department of Medicine A, University Hospital Münster, Münster, 48149 Germany; 9 Institute of Pathology, University Medical Center, Göttingen, 37075 Germany; 10 Gerhard Domagk Institute for Pathology, University Hospital Münster, Münster, 48149 Germany; 11 Department of Haematology, Cambridge Institute of Medical Research, Cambridge University, Cambridge, CB2 0XY UK; 12 Industrial Biotechnology Centre and School of Biosciences, University of Kent, Canterbury, CT2 7NJ UK; 13 Fraunhofer Institute for Molecular Biology and Applied Ecology (IME), Project group Translational Medicine and Pharmacology (TMP), Frankfurt, 60596 Germany; 14 Max von Pettenkofer Institute, Virology, Faculty of Medicine, LMU München, Munich, 80336 Germany

**Keywords:** Cancer, Biomarkers, Oncology

## Abstract

Hypomethylating agents decitabine and azacytidine are regarded as interchangeable in the treatment of acute myeloid leukemia (AML). However, their mechanisms of action remain incompletely understood, and predictive biomarkers for HMA efficacy are lacking. Here, we show that the bioactive metabolite decitabine triphosphate, but not azacytidine triphosphate, functions as activator and substrate of the triphosphohydrolase SAMHD1 and is subject to SAMHD1-mediated inactivation. Retrospective immunohistochemical analysis of bone marrow specimens from AML patients at diagnosis revealed that SAMHD1 expression in leukemic cells inversely correlates with clinical response to decitabine, but not to azacytidine. SAMHD1 ablation increases the antileukemic activity of decitabine in AML cell lines, primary leukemic blasts, and xenograft models. AML cells acquire resistance to decitabine partly by SAMHD1 up-regulation. Together, our data suggest that SAMHD1 is a biomarker for the stratified use of hypomethylating agents in AML patients and a potential target for the treatment of decitabine-resistant leukemia.

## Abstract (web summary)

In acute myeloid leukemia, hypomethylating agents decitabine and azacytidine are used interchangeably. Here, the authors show that the major metabolite of decitabine, but not azacytidine, is subject to SAMHD1 inactivation, highlighting SAMHD1 as a potential biomarker and therapeutic target

## Introduction

DNA hypomethylating agents (HMAs), including the azanucleosides decitabine (DAC, 5-aza-2′-deoxycytidine) and azacytidine (AZA, 5-azacytidine), have emerged as less toxic alternative treatment options for myelodysplastic syndrome (MDS) and acute myeloid leukemia (AML) patients who cannot tolerate intensive chemotherapy. Several clinical trials have revealed a significant benefit of DAC and AZA treatment over alternative care regimens (predominantly best supportive care or low-dose Ara-C) with overall response rates ranging from 10 to 70%^1–4^. Because in some patients responses are restricted to either of the drugs, predictive biomarkers for HMAs are urgently needed to enable personalized drug selection. Currently, both drugs are frequently considered to be equivalent in clinical practice^5–7^. However, there is a lack of HMA head-to-head comparisons, and the understanding of their mechanisms of action is incomplete. Moreover, guadecitabine (SGI-110), a dinucleotide of DAC and deoxyguanosine and DAC prodrug, is under clinical investigation as additional HMA for AML treatment^8^.

**Fig. 1:**
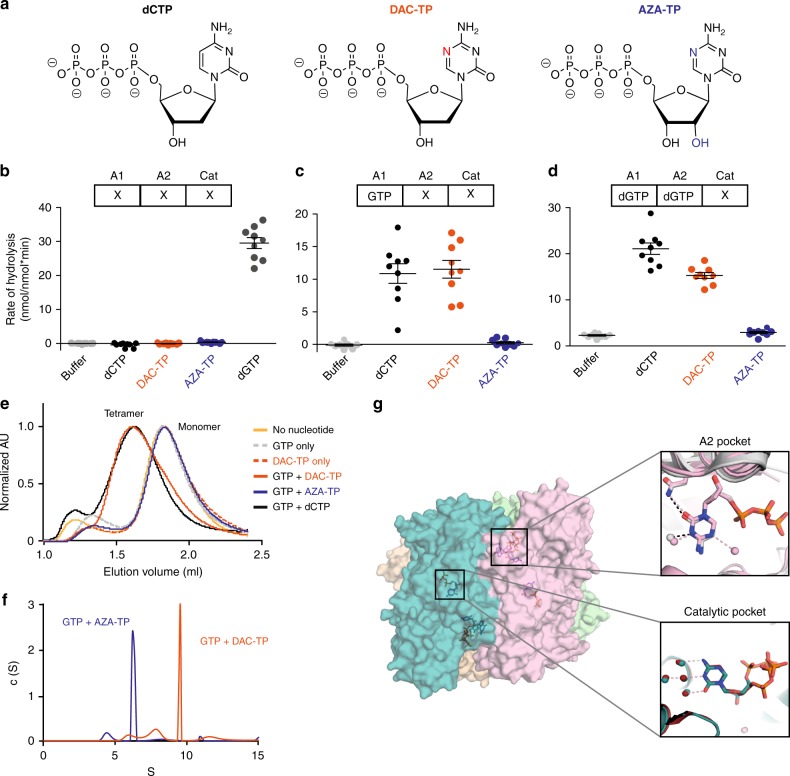
DAC-TP, but not AZA-TP, is an allosteric activator and substrate of SAMHD1. **a** Chemical structures of dCTP, decitabine triphosphate (DAC-TP), and azacytidine triphosphate (AZA-TP). **b**–**d** dNTPase activity assay for recombinant SAMHD1. The rate of hydrolysis for the indicated nucleotide triphosphates was quantified in the presence of (**b**) buffer alone, (**c**) GTP, which can occupy allosteric site 1 (A1), or (**d**) dGTP, which can occupy A1 and allosteric site 2 (A2). Cat: Catalytic site. **e** Size-exclusion chromatograms of SAMHD1 in absence of nucleotide (yellow line) or in the presence of GTP alone (gray dashed line), DAC-TP alone (orange dashed line), GTP + DAC-TP (orange line), GTP + AZA-TP (blue line), or GTP + dCTP (black line). **f** Sedimentation velocity of SAMHD1 incubated in the presence of GTP in combination with either AZA-TP or DAC-TP. **g** Structure of the SAMHD1 tetramer in complex with DAC-TP. Each SAMHD1 subunit is shown as a surface representation, colored in light pink, deep teal, light green and wheat. In the insets, nucleotides are shown as sticks. (Top, right panel) An overlay of SAMHD1 structures in complex with dCTP (PDB ID: 4TNP; gray) or DAC-TP (light pink) in allosteric site 2. (Bottom, right panel) An overlay of SAMHD1 structures in complex with dCTP (PDB ID: 4TNP; gray) or DAC-TP (deep teal) in the catalytic pocket

 AZA and DAC are structurally related cytidine nucleoside analogs (Fig. 1a) that become intracellularly activated by triphosphorylation (Supplementary Fig. 1)^9,10^. DAC triphosphate (DAC-TP) is exclusively incorporated into DNA, while AZA-TP is incorporated primarily into RNA. However, about 10–35% of AZA are metabolized into DAC-TP, following the intracellular conversion of AZA diphosphate (AZA-DP) by ribonucleotide reductase (RNR) to DAC-DP (Supplementary Fig. 1)^11,12^. AZA- and DAC-derived DAC-TP can therefore exert identical effects, including depletion of DNA methyltransferases (DNMTs), suppression of DNA methylation, and induction of DNA damage and apoptosis^9,10^. AZA-TP incorporation into RNA results in AZA-specific effects, such as disruption of transcription and protein synthesis (Supplementary Fig. 1)^9,10^. Since suppression of DNA methylation was not found to correlate well with HMA efficacy in AML and MDS patients^13,14^, the exact modes of the antileukemic action of AZA and DAC remain to be elucidated.

The sterile alpha motif and histidine-aspartate domain-containing protein 1 (SAMHD1) is a 2′-deoxynucleoside-5′-triphospohate (dNTP) triphosphohydrolase that cleaves physiological dNTPs (but not ribose-based NTPs) into 2′-deoxynucleosides and inorganic triphosphate^15,16^. The triphosphohydrolase activity of SAMHD1 requires the assembly of a homotetramer complex, which is regulated by the binding of GTP or dGTP to the allosteric site 1 (A1) and any canonical dNTP to the allosteric site 2 (A2)^17,18^ (Supplementary Fig. 2a). Recently, SAMHD1 was found to hydrolyze cytarabine triphosphate (Ara-CTP) and several other nucleoside analog triphosphates used for the treatment of leukemia^19–21^. Moreover, SAMHD1 was identified as a biomarker that is predictive for the clinical response of AML patients to Ara-C-based therapy^19,22^. Independently of its triphosphohydrolase function, SAMHD1 may further counteract the activity of DNA-damaging drugs through promotion of a cellular nuclease activity involved in DNA end resection, which facilitates DNA double-strand break (DSB) repair by homologous recombination (HR)^23^. However, the relevance of this additional mechanism has not been investigated in the context of nucleoside analog treatment of AML, yet.

Previous observations suggested that SAMHD1 interacts with DAC-TP^21^ and influences DAC efficacy in leukemic cell lines^24^. However, detailed mechanistic and functional studies are currently lacking and the effect of SAMHD1 on DAC or AZA responses in a clinical context remains unknown. Therefore, we here investigate the interaction of SAMHD1 with DAC-TP and AZA-TP and the consequences that this may have on their antileukemic activities in AML patients. We show that DAC-TP is both an activator and a substrate of SAMHD1. Its triphosphohydrolase activity, but not nuclease-promoting activity, determines the therapeutic efficacy of DAC and SGI-110, while that of AZA remains unaffected. Thus, SAMHD1 is a clinically relevant DAC resistance factor and a predictive biomarker for DAC (and SGI-110) stratification in AML.

## Results

### DAC-TP is an allosteric activator and substrate of SAMHD1

DAC-TP and AZA-TP both contain the same modified nucleobase that closely resembles cytosine, except for the carbon at position 5 of the base that is replaced with a nitrogen atom. The only difference between these two nucleotide analogs is that DAC-TP contains a 2′-deoxyribose sugar, while AZA-TP possesses a ribose sugar with a 2′-hydroxyl group (Fig. 1a).

Neither AZA-TP nor DAC-TP were hydrolyzed by SAMHD1 in the absence of additional activators in an in vitro enzymatic assay, suggesting that they cannot bind both allosteric sites to activate the enzyme (Fig. 1b). However, when GTP was added to the mixture, DAC-TP (but not AZA-TP) was hydrolyzed by SAMHD1 just as efficiently as canonical dCTP (Fig. 1c). As GTP can only bind the A1 site^18^, this implies that DAC-TP can occupy the A2 site to activate SAMHD1-mediated hydrolysis. In an established SAMHD1 pre-assembly assay^17,18^, SAMHD1 tetramers were pre-assembled in the presence of dGTP and subsequently diluted into either reaction buffer alone or buffer containing dCTP, DAC-TP, or AZA-TP (Fig. 1d). dCTP and DAC-TP, but not AZA-TP, were hydrolyzed, confirming that DAC-TP, but not AZA-TP, is a substrate of SAMHD1 enzyme.

To confirm our finding that DAC-TP can promote SAMHD1 tetramerization by binding to the A2 site, we monitored the oligomerization state of SAMHD1 in the presence of the A1-site activator GTP following addition of either DAC-TP or AZA-TP. Size-exclusion chromatography revealed that DAC-TP, but not AZA-TP, induced the formation of higher-order SAMHD1 oligomers (Fig. 1e). In line with these results, the sedimentation velocity measured by an analytical ultracentrifugation assay confirmed that DAC-TP, but not AZA-TP, induces the formation of SAMHD1 tetramers (Fig. 1f).

To elucidate how DAC-TP binds the catalytic pocket and A2 site of SAMHD1, DAC-TP was co-crystallized with the inactivated catalytic domain of SAMHD1 (residues 113–626 with H206R and D207N mutations), which has been extensively used to study nucleotide binding to SAMHD1^17,18^. The crystal structure of the complex, determined at a 2 Å resolution (Supplementary Table 1), demonstrated that DAC-TP molecules can occupy both the A2 pockets and catalytic sites of the SAMHD1 tetramer (Fig. 1g). Comparison of the DAC-TP/SAMHD1 structure (PDB ID code 6CM2) with a previously determined dCTP/SAMHD1 structure (PDB ID 4TNP)^18^ showed that the two structures align very well with an overall root-mean-square deviation of ~0.4 Å. The hydrogen-bonding interactions between the SAMHD1 catalytic pocket and DAC-TP are the same as those involved in dCTP binding. Although an additional water molecule forms a hydrogen bond with the position five nitrogen moiety that is only present in the base of DAC-TP, this extra water molecule, interestingly, did not engage in any interaction with SAMHD1. Moreover, SAMHD1’s catalytic pocket was not altered by the interaction with DAC-TP, suggesting that DAC-TP adopts a binding mode similar to that of canonical dCTP (Fig. 1g, lower inset). In line with our biochemical analyses (Fig. 1b–d), AZA-TP did not co-crystallize with SAMHD1. Taken together, DAC-TP is an allosteric activator and substrate of SAMHD1, while AZA-TP does not interact with SAMHD1 (Supplementary Fig. 2c).

### SAMHD1 expression controls DAC-TP levels and cytotoxicity

**Fig. 2:**
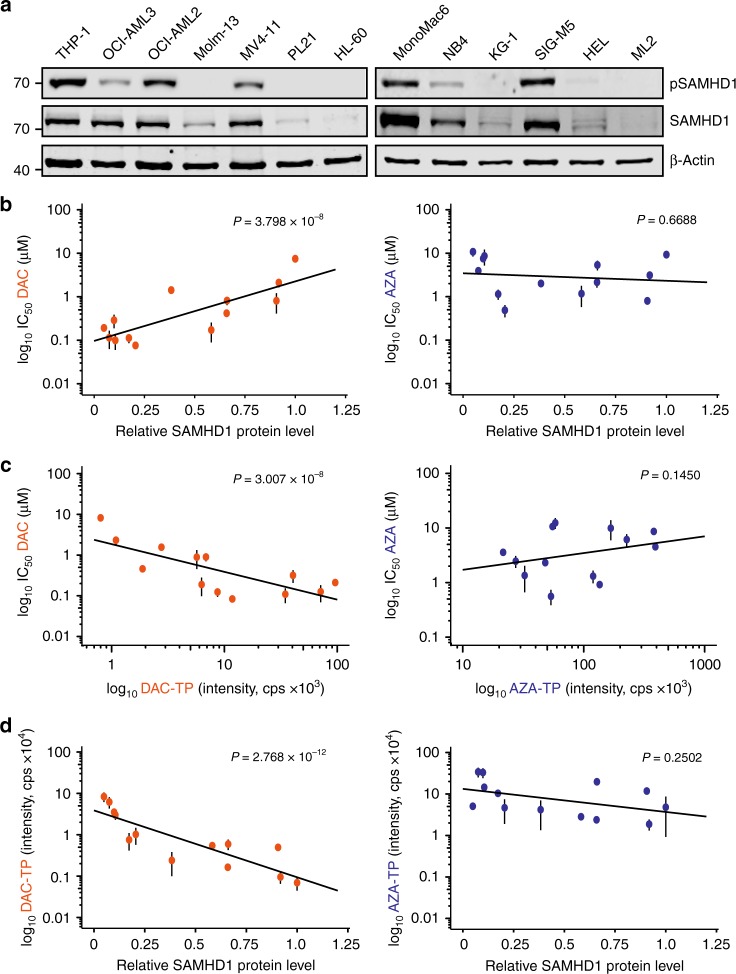
SAMHD1 expression levels are inversely correlated with DAC efficacy. **a** Representative immunoblots of SAMHD1 and phospho-SAMHD1 in the AML cell lines indicated. β-Actin served as a loading control. **b**–**d** Correlation analyses for DAC (orange cirles) and AZA (dark blue circles) in AML cell lines shown in (**a**) between (**b**) IC_50_ values and cells’ relative expression levels of SAMHD1, or **c** IC_50_ values and their corresponding triphosphate levels determined by LC–MS/MS, or **d** triphosphate levels and relative expression levels of SAMHD1. Expression levels were normalized to β-actin and are shown as arbitrary units (a.u.); the relative expression of each protein in THP-1 cells was set to 1. For (**b**–**d**), closed circles and error bars represent means ± s.d. of three independent experiments, each performed in three technical replicates. Data were analyzed using a generalized log-logistic model. *P*-values for goodness-of-fit were computed using the likelihood-ratio (chi-squared) test

To investigate whether SAMHD1 expression may differentially affect the cytotoxicity of DAC and AZA, we tested their effects in a panel of human AML cell lines with differential SAMHD1 expression (Fig. 2a). In addition, we included the investigational drug SGI-110 in these analyses. SGI-110 is a dinucleotide of DAC and deoxyguanosine, which is intracellularly converted to DAC and has shown promising preclinical and clinical activity^8^. The activity of these three drugs against AML cell lines was determined by the half-maximal inhibitory concentration (IC_50_). We found that levels of SAMHD1 protein (Fig. 2a) and *SAMHD1* mRNA (Supplementary Fig. 3) were inversely correlated with cytotoxicity of DAC and SGI-110, but not of AZA (Fig. 2b, Supplementary Figs 4, 5, 6a, b, and Supplementary Table 2). Notably, the IC_50_ values of DAC correlated with those of SGI-110 (Supplementary Fig. 6c), but not with those of AZA (Supplementary Fig. 6d) or of non-nucleoside cancer drugs, including the topoisomerase II inhibitors daunorubicin and etoposide, the topoisomerase I inhibitors topotecan, and the alkylating agents lomustine and melphalan (Supplementary Fig. 7).

Next, intracellular DAC-TP and AZA-TP concentrations were measured by liquid chromatography-coupled tandem mass spectrometry (LC–MS/MS) in AML cell lines displaying varying SAMHD1 levels. DAC-TP, but not AZA-TP, concentrations correlated with drug efficacy (Fig. 2c). Moreover, SAMHD1 protein levels were inversely correlated with DAC-TP, but not AZA-TP, concentrations (Fig. 2d). In addition, SAMHD1 levels correlated with endogenous dNTP concentrations, but no correlation was observed between the antileukemic efficacy of DAC or AZA and the cellular dNTP concentrations (Supplementary Fig. 8). The levels of other proteins involved in either HMA uptake (including the human equlibrative nucleoside transporter (hENT1; SLC29A1) and the ergothioneine transporter (OCTN1)) or HMA metabolism (including deoxycytidine kinase (DCK), uridine-cytidine kinase 1 and 2 (UCK1 and UCK2), cytidine deaminase (CDA), deoxycytidilate deaminase (DCTD), and RNR large and small subunit (RNR1 and RNR2)) (Supplementary Fig. 1)^9,25^ did not correlate with the cytotoxic activity of either AZA or DAC in AML cell lines (Supplementary Figs 9 and 10). Collectively, these results further confirm that the antileukemic potency of DAC, but not of the structurally closely related AZA, is correlated to SAMHD1 triphosphohydrolase activity in AML cell lines.

Interestingly, SAMHD1 phosphorylation at threonine 592 (T592) was detected in most AML cell lines (Fig. 2a), although T592 phosphorylation had in the past been suggested to be primarily associated with a loss of triphosphohydrolase activity during the S-phase of the cell cycle^26–28^. Our results are more in line with findings by Tramentozzi et al., which showed that the enzymatic function of SAMHD1 can remain active during the entire cell cycle irrespectively of its phosphorylation status^29^.

### Modulation of SAMHD1 expression affects DAC efficiency

**Fig. 3:**
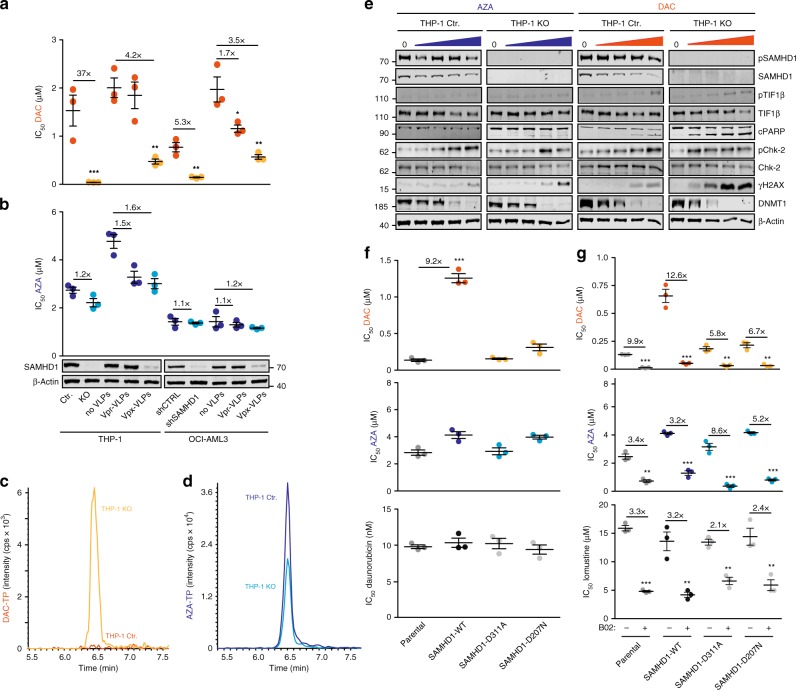
SAMHD1 triphosphohydrolase activity controls DAC response. **a**, **b** IC_50_ values for (**a**) DAC or (**b**) AZA in AML cell lines THP-1 and OCI-AML3 with experimentally modulated SAMHD1 levels. SAMHD1 was depleted by either CRISPR/Cas9-mediated *SAMHD1* knockout in THP-1 cells (THP-1 KO), by shRNA-mediated silencing of *SAMHD1* in OCI-AML3 cells, or by transduction with VLPs carrying SAMHD1-degrading Vpx protein (Vpx-VLPs). Unedited THP-1 cells (THP-1 Ctr.), cells transduced with control-shRNAs (shCTRL), or cells treated with Vpr-carrying VLPs (Vpr-VLPs) served as controls. Each point represents an average of three technical replicates, and in each case, as shown, three independent experiments were performed. Horizontal lines and error bars represent means ± s.d. The numbers above the data points indicate the factor of decrease of IC_50_ values in SAMHD1-depleted cells relative to control cells. Representative SAMHD1 immunoblots for the different experimental conditions are shown. β-Actin served as a loading control. **c**, **d** Representative LC–MS/MS analyses of (**c**) DAC-TP levels or (**d**) AZA-TP levels in THP-1 Ctr. or THP-1 KO cells. **e** Representative immunoblots of proteins involved in DNA damage response and DNA methylation in THP-1 Ctr. or THP-1 KO cells following treatment with increasing concentrations (0, 0.08, 0.4, 2, and 10 µM) of either AZA (left panels) or DAC (right panels) for 72 h. **f**, **g** IC_50_ values of (**f**) DAC, AZA and daunorubicin or (**g**) DAC, AZA and lomustine without or with the RAD51 inhibitor B02 in parental HEL cells and HEL cells overexpressing either SAMHD1-WT or the dNTPase-defective SAMHD1 mutants SAMHD1-D311A or SAMHD1-D207N. Each closed circle represents a technical replicate (*n* = 3) of one representative experiment out of three. Horizontal lines and error bars represent mean ± s.d. Numbers indicate the factor of decrease of IC_50_ values in SAMHD1-depleted relative to control cells. Statistical analyses were performed using unpaired two-tailed Students’ *t*-test. **p < 0.01; ***p < 0.001

Next, we investigated the impact of SAMHD1 deficiency on DAC activity by different approaches: (i) CRISPR–Cas9-mediated *SAMHD1* gene disruption in AML cell lines exhibiting high endogenous SAMHD1 levels, (ii) *SAMHD1* knockdown using lentiviral vectors encoding shRNA, or (iii) targeted degradation of SAMHD1 with virus-like particles (VLPs) that shuttle the SAMHD1-interacting lentiviral Vpx protein (Vpx-VLPs) into cells^19^. Vpx recruits SAMHD1 to a cullin4A-RING E3 ubiquitin ligase (CRL4DCAF1), which targets SAMHD1 for proteasomal degradation^28^. In AML cells, *SAMHD1* knockout (THP-1 KO), *SAMHD1* knockdown (OCI-AML3), and SAMHD1 depletion by transduction with Vpx-VLPs (THP-1 and OCI-AML3) markedly increased the efficacy of DAC treatment (3.5–37-fold), but not of AZA treatment (1.2–1.6-fold) (Fig. 3a, b; Supplementary Fig. 11). Similarly, the DAC-based dinucleotide SGI-110 showed enhanced cytotoxicity in SAMHD1-deficient cells (Supplementary Fig. 12).

Next, we constitutively expressed wild-type SAMHD1 (SAMHD1-WT) or the triphosphohydrolase-defective mutant SAMHD1-D311A^19^ in HEL cells, which are characterized by low SAMHD1 levels (Supplementary Fig. 13a). Constitutive expression of SAMHD1-WT, but not of SAMHD1-D311A, decreased the cytotoxicity of DAC and SGI-110, but not of AZA (Supplementary Fig. 13b-d). Furthermore, DAC-TP levels (and to a lesser extent endogenous dNTPs; Supplementary Fig. 14a) were elevated in SAMHD1-deficient THP1 KO cells compared with THP-1 control cells (Fig. 3c), while AZA-TP levels were not increased upon ablation of SAMHD1 (Fig. 3d). Similar drug and dNTP responses were observed in the SAMHD1 triphosphohydrolase-functional and -defective HEL cell lines (Supplementary Figs 13e,f and 14b).

About 10–35% of AZA can be converted into DAC-TP^11,12^ (Supplementary Fig. 1). Consequently, DAC-TP was readily detectable in AZA-treated THP-1 KO cells, but, interestingly, hardly detectable in AZA-treated THP-1 control cells (Supplementary Fig. 15a). Similarly, DAC-TP was readily found in AZA-treated parental HEL cells and HEL cells expressing the catalytically inactive SAMHD1-D311A, while being barely detectable in HEL SAMHD1-WT cells (Supplementary Fig. 15b). These results show that SAMHD1’s triphosphohydrolase activity controls the intracellular DAC-TP levels following both DAC and AZA treatment of AML cells, while AZA-TP levels are not affected.

### Effects of SAMHD1 on DAC-induced apoptosis and DNA damage

Next, we explored the degree to which SAMHD1 may influence DNA damage and apoptosis induction by DAC and AZA. In THP-1 KO cells, DAC treatment elicited an enhanced concentration-dependent DNA damage response compared with THP-1 control cells, as indicated by increased levels of phosphorylated histone H2AX (ser139) (ɣH2AX), phosphorylated Chk2 (Thr68), and phosphorylated TIF1β (ser824) (Fig. 3e, right panels). Similarly, enhanced levels of apoptosis, as indicated by PARP1 cleavage (Fig. 3e) and increased fractions of sub-G1 cells (Supplementary Fig. 16a), were observed at lower concentrations of DAC in SAMHD1-deficient THP-1 KO cells compared with THP-1 control cells. In AZA-treated THP-1 cells, SAMHD1 exerted less pronounced effects on phosphorylated ɣH2AX, Chk2, TIF1β, cPARP1, and sub-G1 cells, which were only observed at the highest AZA concentrations in THP-1 KO cells (Fig. 3e, left panels and Supplementary Fig. 16b).

DAC and AZA may promote DNA damage through induction of DNA DSBs resulting in repair processes including non-homologous end joining and/or HR^11,30,31^. SAMHD1 was recently shown to facilitate HR-mediated DSB repair independently of its triphosphohydrolase function by promoting DNA end resection through direct interaction with MRE11/CtIP and stimulation of their nuclease activity^23,32^. In human carcinoma and osteosarcoma cell lines, SAMHD1 overexpression decreased the efficacy of DSB-inducing drugs such as topoisomerase inhibitors or IR, while SAMHD1 depletion was associated with increased efficacy of these treatments^23^. To investigate a potential role of triphosphohydrolase-independent SAMHD1 effects on the antileukemic drug activity, we first studied the impact of SAMHD1 on the toxicity of non-nucleoside DSB-inducing drugs that do not depend on SAMHD1’s triphosphohydrolase activity. The topoisomerase II inhibitors daunorubicin or etoposide, the topoisomerase I inhibitor topotecan, and the alkylating agents lomustine or melphalan showed similar cytotoxicity and induced markers of DNA damage and apoptosis at similar concentrations in THP-1 KO and THP-1 control cells, irrespective of their SAMHD1 status (Supplementary Figs 16c, and 17-19). Next, we tested whether overexpression of the SAMHD1 mutants D207N and D311A, which are both characterized by disrupted triphosphohydrolase function but sustained nuclease-promoting activity^23,33,34^, in HEL cells may alleviate the efficacy of HMAs or non-nucleoside drugs including daunorubicin and lomustine. Only SAMHD1-WT significantly decreased toxicity of DAC, but not of AZA or any other triphosphohydrolase activity-independent agents, while SAMHD1 mutants D311A and D207 did not influence the activity of any of these drugs including DAC (Fig. 3f and Supplementary Fig. 20). To test whether inhibition of enzymes involved in HR other than SAMHD1 may influence the activity of HMAs, parental HEL cells and HEL cells expressing either SAMHD1-WT or the triphosphohydrolase-deficient mutants were treated with the RAD51 inhibitor B02^31,35^, which prevents HR downstream of MRE11/CtIP/SAMHD1 activity^23,32^. In line with previous reports^31,35^, B02 markedly increased toxicity of DAC, AZA, and lomustine (Fig. 3g), confirming that interference with HR can in principle affect the antileukemic activity of these drugs. Together, these data suggest that SAMHD1 does not significantly influence the efficacy of HMAs through its nuclease-promoting functions in AML cells.

### SAMHD1 regulates DAC-TP-mediated methylation changes

DNA (cytosine-5)-methyltransferase 1 (DNMT1) depletion and changes in global DNA methylation induced by DAC and AZA were investigated in the THP-1 model cell lines. Incorporation of DAC-TP into DNA results in the formation of adducts between DNA and DNMT1, which are degraded by the proteasome, resulting in decreased DNA methylation^9,10^. Therefore, DNMT1 depletion and suppression of global DNA methylation may be used as downstream surrogates for bioactive DAC-TP levels. DAC-induced DNMT1 depletion (Fig. 3e, right panels) and suppressed DNA methylation (Supplementary Fig. 21a) at lower concentrations in THP-1 KO cells compared with THP-1 control cells. The maximum effect of DAC on DNA methylation was achieved at a concentration of 0.08 µM in THP-1 KO cells (Supplementary Fig. 21a). AZA affected DNMT1 levels and DNA methylation only at higher drug concentrations than DAC. Again, these effects were more pronounced in THP-1 KO cells than in SAMHD1-expressing cells (Fig. 3e, left panels and Supplementary Fig. 21b). Together, this indicates that SAMHD1 also affects demethylation caused by AZA-derived DAC-TP.

### AML cell adaptation to HMAs results in SAMHD1 upregulation

**Fig. 4:**
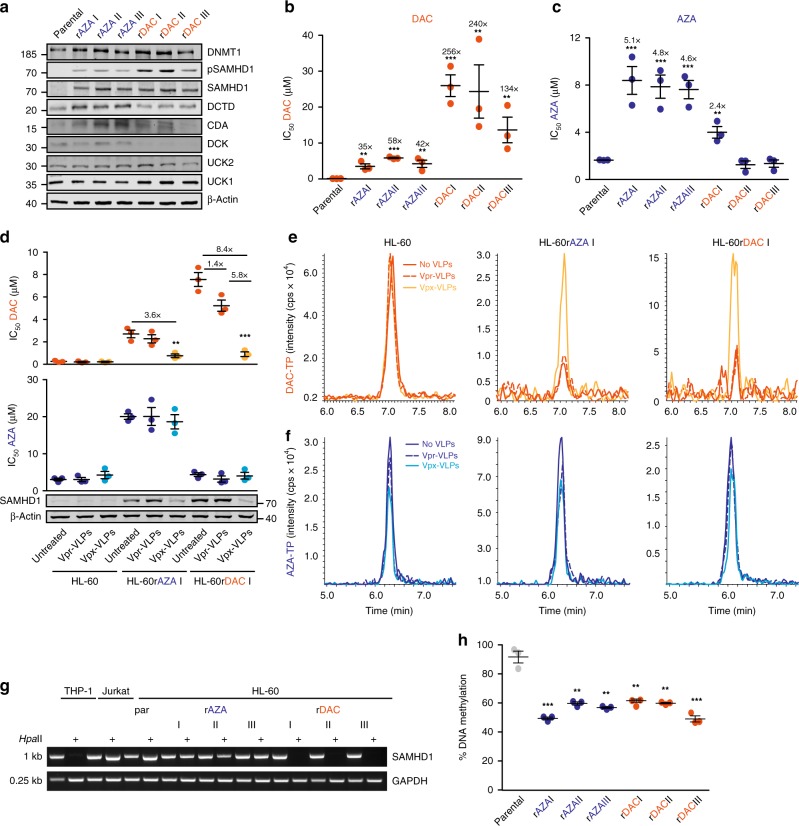
HMA resistance development in HL-60 cells results in SAMHD1 upregulation. **a** Representative immunoblots of SAMHD1, other proteins involved in AZA and DAC metabolism, and DNA methylation (DNMT1) in parental HL-60 cells and in three AZA- or DAC-resistant HL-60 sublines (rAZA I–III, rDAC I–III). β-Actin served as a loading control. **b**, **c** IC_50_ values for (**b**) DAC and (**c**) AZA in AZA- and DAC-resistant cell lines relative to the parental counterpart. Each circle represents a technical replicate (*n* = 3) of three independent experiments performed. Horizontal lines and error bars represent means ± s.d. The numbers above the data points indicate the factor of increase of IC_50_ values in resistant cells relative to parental HL-60 cells. **d** Parental, AZA- and DAC-resistant HL-60 cells were treated with the VLPs indicated and subsequently analyzed for AZA and DAC cytotoxicity (top) and SAMHD1 expression (bottom). IC_50_ values of three independent experiments each performed with technical replicates (*n* = 3) are presented as closed circles. Horizontal lines and error bars represent means ± s.d. Numbers above the bars indicate the factor of decrease in IC_50_ values in Vpx-VLP-treated cells relative to Vpr-VLP-treated controls. **e**, **f** Representative LC–MS/MS analyses of (**e**) DAC-TP and (**f**) AZA-TP in parental and AZA- or DAC-resistant HL-60 cells, treated with Vpx-VLPs (yellow/light blue chromatogram), with control Vpr-VLPs (dashed orange and dark blue chromatogram) or left untreated (orange and dark blue chromatogram). **g** Analysis of *SAMHD1* promotor methylation through amplification of a single PCR product (993-bp) corresponding to the promoter sequence after *Hpa*II digestion. A 0.25-kb fragment of the GAPDH gene lacking *Hpa*II sites was PCR-amplified using the same template DNA served as loading control, THP-1 and Jurkat cells served as positive and negative controls, respectively. **h** Global DNA methylation determined by measurement methylation levels of long interspersed element (LINE)-1 in parental HL-60 cells and rAZA or rDAC HL-60 sublines. Each circle represents means ± s.d. of a technical replicate (*n* *=* 3) of one representative experiments out of three

Next, we explored the potential role of SAMHD1 in acquired resistance formation to HMAs. SAMHD1-low-expressing HL-60 cells were gradually adapted over a period of at least 6 months to proliferation in the presence of either DAC (1 µM) or AZA (10 µM), concentrations that are within the range of clinically achievable steady-state plasma levels of these HMAs (DAC: 0.3–1.6 µM; AZA: 3–11 µM)^36–38^. Short-term (3 days) exposure of parental HL-60 cells with DAC or AZA did not result in increased SAMHD1 expression, although DNMT1 levels and global DNA methylation were already found to be suppressed (Supplementary Fig. 22). In contrast, all three long-term selected, AZA-resistant (rAZA; I–III) and rDAC HL-60 sublines displayed increased SAMHD1 expression at the protein (Fig. 4a) and mRNA levels (Supplementary Fig. 23). rDAC sublines were highly resistant to DAC (Fig. 4b), but not or only marginally resistant to AZA (Fig. 4c). In contrast, HL-60 cells adapted to AZA displayed considerable cross-resistance to DAC, ranging from 35- to 58-fold (Fig. 4b, c). Targeted degradation of SAMHD1 using Vpx-VLPs sensitized both rDAC and rAZA HL-60 sublines, but not parental HL-60 cells, to DAC-induced cytotoxicity (Fig. 4d, upper panel). This differential response was mirrored by the respective intracellular DAC-TP levels (Fig. 4e). In contrast, the cytotoxic response to AZA (Fig. 4d, lower panel) and AZA-TP levels (Fig. 4f) were unaffected by Vpx-VLP exposure and SAMHD1 depletion. Collectively, these results suggest that long-term treatment of AML cells with HMAs, the bioactive metabolites of which are fully (DAC) or only in part (AZA) SAMHD1-dependent, results in SAMHD1-overexpressing leukemic cells. We also observed that the sublines rDACI to III displayed decreased levels of DCK compared with parental HL-60 cells. In addition, rDACI and II cells, but not rDACIII, displayed increased CDA levels (Fig. 4a). The sublines rAZAI to III displayed increased levels of DCTD in addition to enhanced CDA levels (Fig. 4a). These differences indicate that HL-60 adaptation to DAC and AZA can, in addition to SAMHD1 upregulation, be associated with further resistance mechanisms that partly differ between the individual HMA-resistant sublines.

Cellular SAMHD1 levels may be regulated by mechanisms, including DNA promoter methylation, proteasomal SAMHD1 degradation, and autophagy^39–41^. We tested whether SAMHD1 overexpression in HL-60 sublines resistant to DAC or AZA may be associated with demethylation of the *SAMHD1* gene promoter. PCR amplification of genomic DNA treated with the methylation-sensitive *Hpa*II endonuclease revealed a single PCR product corresponding to the size of the *SAMHD1* promoter (1 kb) in both *Hpa*II treated and untreated parental HL-60 cells indicating methylation of the promoter (Fig. 4g), but not in genomic DNA from rDAC HL-60 and THP-1 cells, which served as a control for an unmethylated *SAMHD1* promoter^40^. This shows that the *SAMHD1* promoter is primarily in an unmethylated state in HL-60 cells with acquired resistance to DAC. Interestingly, the *SAMHD1* promoter appeared to be primarily in a methylated state in rAZA HL-60 sublines (Fig. 4g), albeit the global DNA methylation was suppressed in both rAZA HL-60 and rDAC HL-60 sublines to a similar extent (Fig. 4h). These results indicate that different molecular mechanisms underlie SAMHD1 overexpression during AZA and DAC resistance formation.

### SAMHD1 predicts DAC response in AML xenotransplant models

**Fig. 5:**
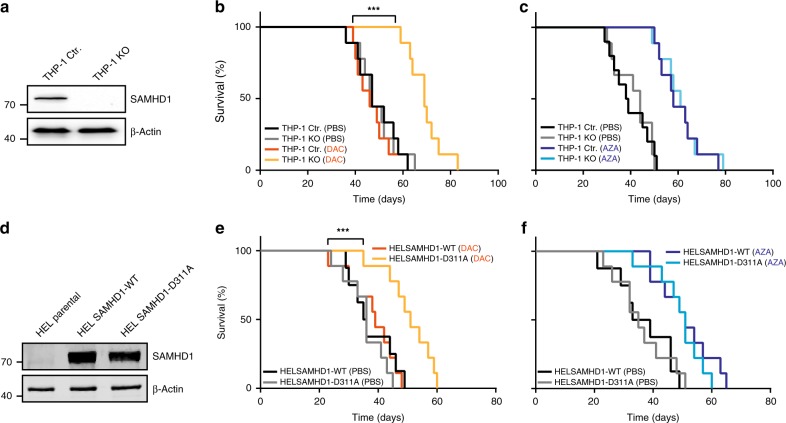
SAMHD1 expression in xenotransplanted AMLs predicts the response to DAC. **a**, **d** SAMHD1 protein expression in transplanted cell lines determined by immunoblotting. **b**, **c**, **e**, **f** Kaplan–Meier survival analyses of NSG mice transplanted with either (**b**, **c**) THP-1 control or THP-1 KO cells or (**e**, **f**) HEL cells expressing SAMHD1-wild-type (WT) or the D311A mutant. **b**, **e** DAC was administered i.p. (0.4 mg/kg twice per week), **c**, **f** AZA was administered i.v. (2.8 mg/kg 5× per week); PBS (control treatment). *P*-values are from the Mantel Cox (Logrank) test. ***P < 0.0001

To investigate whether SAMHD1 expression differentially influences the anti-tumor activity of DAC and AZA in vivo, immunodeficient NSG mice, transplanted with the human AML cell lines genetically engineered to express different levels of SAMHD1, were treated with these HMAs. Mice transplanted with THP-1 KO cells survived significantly longer than mice transplanted with THP-1 control cells in response to DAC (Fig. 5a, b). In contrast, no SAMHD1 dependency was observed in the survival of AZA-treated (Fig. 5c) or PBS-treated mice (Fig. 5b, c). In line with these findings, mice bearing HEL SAMHD1-D311A xenografts survived significantly longer than their HEL SAMHD1-WT-bearing counterparts, when treated with DAC, but not with AZA or PBS (Fig. 5d–f and Supplementary Fig. 24).

### SAMHD1 determines the clinical response to DAC in AML

**Fig. 6:**
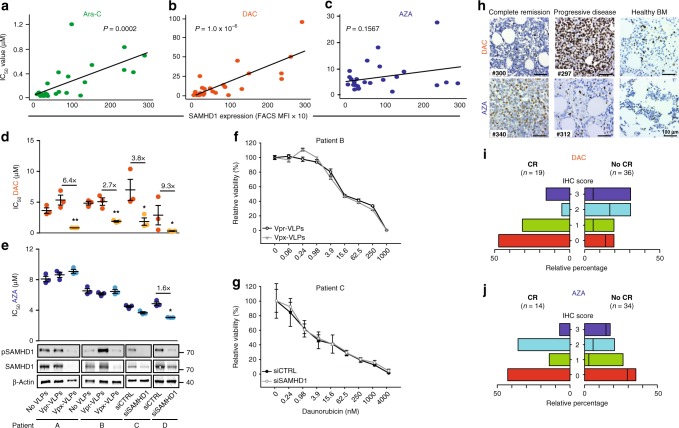
SAMHD1 expression is a predictive biomarker for DAC therapy in AML. **a**–**c** Correlative analyses of SAMHD1 expression and cytotoxicity for (**a**) Ara-C, (**b**) DAC, or (**c**) AZA was performed in bone-marrow-derived blasts from therapy-naive AML patients (blast purities of > 80%). SAMHD1 expression was analyzed by flow cytometry. IC_50_ measurements were determined in parallel. Data were analyzed by using a generalized log-logistic model. The *r2* value indicates goodness-of-fit of the regression model to the data, and represents variance explained by the independent variable divided by total variance of the IC_50_ values for Ara-C. **d**, **e** Blasts from four AML patients were either transduced with Vpx- or Vpr-VLPs or left untreated (no VLPs; patients A, B), or transfected with *SAMHD1-*specific or control siRNAs (patients C, D), and 2 days later, analyzed for cytotoxicity induced by (**d**) AZA or (**e**) DAC. SAMHD1 expression was analyzed by immunoblotting. Horizontal lines and error bars represent means ± s.d. of the IC_50_ values. Circles represent single values of one technical triplicate for each patient. The numbers above the data points indicate the factor by which the SAMHD1-depleted and the control groups differed. Statistical analyses were performed using unpaired two-tailed Students’ *t* test. **f**, **g** Dose–response analyses in patients transduced either with Vpx- or Vpr-VLPs (patient B), or transfected with *SAMHD1-*specific or control siRNAs (patient C) and subsequently treated with different concentrations of daunorubicin and incubated for 96 h before viability was quantified by MTT analyses. Values are mean ± s.d. of triplicates of one technical experiment. **h** Representative IHC micrographs showing SAMHD1 expression in bone marrow (BM) from patients on DAC- or AZA-based therapy with complete remission (CR) (#300, #340) or progressive disease (#297, #312). BM from two healthy donors is shown as reference. Scale bars, 100 μm. **i**, **j** Shown are relative frequencies of patients with IHC scores of 0, 1, 2, or 3 among CR and no-CR patients who had received monotherapy with either (**i**) DAC or (**j**) AZA. The bar for no-CR patients is subdivided into patients with stable disease (left side) and progressive disease (right side) patients

Primary leukemic cells were isolated and enriched from the bone marrow of therapy-naive AML patients (for patients’ characteristics see Supplementary Data file 1). Basal SAMHD1 protein expression levels, quantified by flow cytometry (Supplementary Fig. 25a, b), were correlated with the IC_50_ values of the dNTPase-sensitive drugs, Ara-C and DAC (*P* = 0.0002 and *P* = 1.0 × 10^−6^, respectively), but not of AZA (*P* = 0.1567) (Fig. 6a–c). Next, we evaluated the effects of SAMHD1 depletion by either Vpx-VLPs or siRNA in blasts of four AML patients (Supplementary Data file 1; patients A–D). Transient reduction of SAMHD1 in blasts lowered the IC_50_ values for DAC by 2.7–9.3-fold (Fig. 6d), whereas sensitivity to AZA was largely unaffected (Fig. 6e). In addition, Vpx-VLP- and siRNA-mediated SAMHD1 depletion did not increase the toxicity of the non-nucleoside drug daunorubicin in the blasts of patients B and C (Fig. 6f, g).

Finally, we examined whether SAMHD1 protein levels in leukemic blasts may represent a biomarker for predicting the response to DAC-based therapy. AML blasts in sections of paraffinized bone-marrow specimens taken at primary diagnosis from a multi-centric cohort of adult AML patients, 55 of which received first-line DAC monotherapy and 48 of which received first-line AZA monotherapy (detailed patient characteristics are listed in Supplementary Data file 2), were retrospectively analyzed for SAMHD1 protein levels in blasts by immunohistochemical (IHC) staining and histopathological evaluation in a blinded manner. SAMHD1 levels were markedly increased in the DAC-treated patients, who did not achieve complete remission (progressive disease and stable disease summarized as ‘No CR’) (Fig. 6h, i). In contrast, SAMHD1 levels were not correlated with clinical response in AZA-treated patients (Fig. 6h, j). Of the 55 DAC-treated patients, 19 achieved a CR as best response. Fifteen of these 19 patients were scored as ‘SAMHD1 low’ (IHC score of 0 or 1, 79%, Fig. 6i) and 4 were scored as ‘SAMHD1 high’ (IHC score of 2 or 3, 21%, Fig. 6i) (*P* *=* 0.012 by chi-squared test with 1 degree of freedom). No significant difference was observed between ‘SAMHD1 low’ and ‘SAMHD1 high’ patients in the AZA-treated cohort (*P* *=* 1, Fig. 6j).

## Discussion

**Fig. 7:**
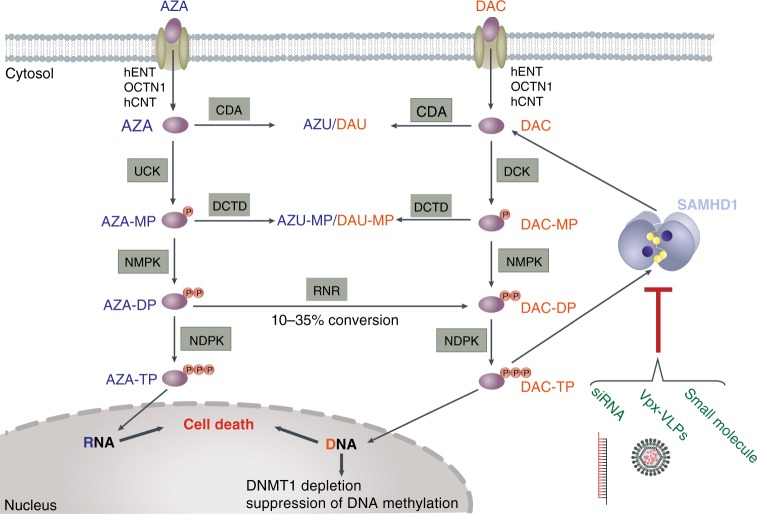
Scheme of SAMHD1’s role in HMA metabolism and effector functions. Metabolic pathway of DAC and AZA.After their cellular uptake by nucleoside-specific transporters, AZA and DAC are activated and metabolically converted into the active nucleotides AZA-TP and DAC-TP, respectively, by sequential phosphorylation events. Importantly, a fraction of the intermediate AZA-DP can be converted to DAC-DP by ribonucleotide reductase (RNR). DAC-TP, but not AZA-TP, can activate and become hydrolyzed by SAMHD1. Strategies to interfere with SAMHD1 expression and function include degradation through Vpx-carrying virus-like particles (Vpx-VLPs), RNA interference or small-molecule inhibitors of SAMHD1’s triphosphohydrolase activity. See also Supplementary Fig. 1 for details, figure created by Dr. Alessia Ruggieri, University of Heidelberg

Our study identified SAMHD1 as a predictive biomarker and a therapeutic target for DAC-based, but not AZA-based therapy, in AML (Fig. 7). Importantly, these findings may also contribute to the mechanistic understanding of previously reported preclinical^11,42^ and clinical results^6,43,44^ that showed differences in efficacy and initial response rates between these two drugs, which are often regarded as interchangeable therapeutic options in AML^5–7^.

A recent report by Knecht et al. has provided detail on the determinants of nucleoside analog triphosphate interactions with the catalytic and allosteric sites of SAMHD1 via the Watson-Crick edge of the base and the sugar and phosphate groups and identified 2’-sugar modifications as a major determinant of nucleotide analog binding to SAMHD1^20^. The allosteric A2 site was only permissive to 2′-deoxyribose-based (e.g., cladribine-TP) nucleotide analogs, but not to arabinose-based (e.g., cytarabine-TP and fludarabine-TP) or 2′-deoxy-2′-fluororibose-based (i.e., clofarabine-TP) nucleotide analogs. Nucleoside analog triphosphates with modifications at the 2′ sugar position, which bind only to the catalytic site, require the presence of other (canonical) nucleotides to induce SAMHD1 tetramerization and activity (Supplementary Fig. 2b). In accordance, our current results show that the 2′-deoxyribose nucleotide DAC-TP (but not the ribose nucleotide AZA-TP) binds to both the allosteric A2 and the catalytic site of SAMHD1, being both a substrate and an activator of the enzyme (Supplementary Fig. 2c). Importantly, our data also demonstrate that a modification at the Hoogsteen edge, the introduction of a nitrogen atom in 5 position of the cytosine base, does not affect DAC-TP binding to SAMHD1 compared with dCTP (Supplementary Fig. 26).

About 10–35% of administered AZA become metabolized into DAC-TP^11,12^. In accordance, we detected substantial amounts of DAC-TP in AZA-treated SAMHD1-deficient AML cells, but only DAC-TP traces in SAMHD1-expressing cells. This indicates that AZA is indeed partially converted into DAC-TP in AML cells and that this AZA-derived DAC-TP is subject to SAMHD1-mediated hydrolysis. In line with these data, DAC-TP-dependent activities of AZA, such as DNMT1 depletion and changes in global DNA methylation, were inhibited by SAMHD1. However, SAMHD1 was not found to interfere with AZA activity in AML cell lines, primary leukemic blasts, and xenotransplanted mice. Moreover, the AZA response of AML patients did not correlate with the SAMHD1 levels in leukemic cells. This indicates that the therapeutic effects of AZA are primarily caused by other mechanisms than conversion into AZA-derived DAC-TP in AML. In accordance, recent clinical investigations in MDS and chronic myelomonocytic leukemia patients reported a lack of correlation between AZA activity and AZA metabolization into DAC-TP^45^.

Recently, SAMHD1 was shown to promote repair of (chemotherapy-induced) DNA DSBs by HR, independently of its triphosphohydrolase function^23,32^. However, our results do not indicate a significant relationship between SAMHD1 and HMA toxicity in AML cells through this mechanism. Moreover, SAMHD1 hydrolyzes and depletes endogenous dNTPs, which has been described to induce replication stress and to increase sensitivity to cytotoxic drugs^46^. However, we observed that high SAMHD1 levels were associated with low DAC-induced cytotoxicity, although they had also resulted in reduced endogenous dNTP concentrations. Consequently, SAMHD1 interferes with the action of DAC predominantly via hydrolysis of DAC-TP in AML cells.

In our models of acquired resistance, the DAC- and AZA-adapted HL-60 sublines displayed multifactorial resistance mechanisms that partially differed between the individual sublines, as indicated by varying expression of HMA-metabolizing enzymes (i.e., DCK, CDA, and DCTD), which have been reported previously to contribute to HMA resistance^14,42^. Interestingly, both DAC- and AZA-adapted HL-60 sublines displayed increased SAMHD1 levels and triphosphohydrolase activity. Since SAMHD1 directly inactivates DAC-TP, it does not come as a surprise that its upregulation represents an acquired DAC resistance mechanism. In AZA-adapted cells, however, the origin of the SAMHD1 upregulation is less intuitive. In a previous study, low DAC doses, which did not cause acute cytotoxic effects, nevertheless elicited antileukemic effects after long-term treatment^47^. Low DAC-TP levels resulting from AZA metabolization, which do not acutely affect AML cell viability, may thus still affect AML cells after long-term exposure. Hence, the increased SAMHD1 levels observed in AZA-adapted cells are likely to be the consequence only of the selection pressure exerted by long-term AML cell exposure to low levels of AZA-derived DAC-TP.

Several biomarkers have been proposed to predict response to HMAs, including mutations in genes coding for the epigenetic enzymes DNMT3A, IDH1/2, TET2, ASXL1, or the tumor suppressor TP53^13,14^. However, none of these candidate biomarkers appears to be sufficiently robust to guide a personalized HMA selection. Our retrospective IHC investigations indicate that SAMHD1 has potential to serve as a biomarker to predict the AML response to DAC (and SGI-110). The expression analyses of specific proteins in tissue sections by IHC is typically based on visual scoring, semi-quantitative by nature and difficult to standardize, despite an evaluation by experienced histopathologists. It has to be noted, however, that IHC scoring is already part of the diagnostic standard work-up in several tumors, including breast, brain, and lung cancers, e.g., in the latter to quantify PD1/PD-L1 expression to guide checkpoint inhibitor therapy^48^. Moreover, qPCR-based or flow cytometry-based quantification of SAMHD1 mRNA or protein levels, respectively, were similarly predictive as IHC scoring of AML blast responses to Ara-C in previous studies^19,22^. *SAMHD1* mutations seem to be rare in AML patients^49^ and are, hence, not suitable as biomarker for DAC therapy.

In conclusion, our findings demonstrate that the activity of DAC, but not of AZA, is affected by the triphosphohydrolase activity of SAMHD1 in AML cells. This indicates substantial differences in the mode of action of these HMAs that are often regarded to be interchangeable in AML therapy. More research will be needed to identify biomarkers that reliably guide the decision on whether patients should be treated with DAC or AZA. Based on our data, however, SAMHD1 has the potential to become a relevant biomarker for the stratification of HMAs in AML patients and a therapeutic target for the improvement of DAC- and SGI-110-based therapies.

## Methods

### Protein expression and purification

N-terminal 6 × His-tagged SAMHD1 constructs were expressed in *Escherichia coli* BL21(DE3). Cells were harvested, resuspended in lysis buffer (50 mM Tris, pH 8, 500 mM NaCl, 20 mM imidazole, 5 mM MgCl_2_, 0.5 mM TCEP), and lysed using a microfluidizer. Lysate was clarified by centrifugation (26,892 × *g* for 25 min). SAMHD1 protein was purified using Ni-NTA affinity and size-exclusion chromatography in SAMHD1 buffer (50 mM Tris-HCl, pH 8.0, 150 mM NaCl, 5 mM MgCl_2_, and 0.5 mM TCEP).

### Analytical size-exclusion chromatography

Purified samples of SAMHD1 (2 mg per ml, 50 μl) mixed with a final concentration of 500 µM GTP and 2–4 mM nucleotide analog were applied to a Superdex 200 5/150 GL column (GE Healthcare) pre-equilibrated with SAMHD1 buffer. The UV absorbance at 280 nm was measured as the protein sample eluted from the column, and values were normalized to their respective peak heights.

### Analytical ultracentrifugation (AUC)

Sedimentation velocity experiments were performed with a Beckman XL-I analytical ultracentrifuge. Samples were prepared with protein concentration of 0.8–1.3 mg/ml in SAMHD1 buffer and equilibrated with a final concentration of 150 μM nucleotides. AUC was performed at 169,167 × *g* and 20 °C with an An60-Ti rotor. The experimental parameters including sample partial specific volume, buffer density and viscosity were calculated with SEDNTERP (http://sednterp.unh.edu/). Velocity data were analyzed with the program SEDFIT^50^.

### Crystallization and data collection

SAMHD1 protein in buffer (50 mM Tris-HCl (pH 8.0), 150 mM NaCl, 5 mM MgCl_2_, and 0.5 mM TCEP) was mixed with 1 mM GTP and 10 mM nucleotide analogs and incubated at 4 °C for 15 min before crystallization. All crystals were grown at 25 °C using the microbatch under-oil method by mixing 1 μL protein (5 mg per ml) with 1 μL crystallization buffer (100 mM SPG (Qiagen), pH 7.4, 25% PEG 1500). Crystals were cryoprotected by crystallization buffer supplemented with 25% (vol/vol) glycerol before being frozen in liquid nitrogen. Diffraction data were collected at the Advanced Photon Source beamline 24-IDC. The data statistics are summarized in Supplementary Table 1.

### Structure determination and refinement

The structures were solved by molecular replacement using PHASER^51^. The previously published SAMHD1 tetramer structure, with all bound nucleotides removed was used as the search model (PDB ID 4BZB). The model was refined with iterative rounds of TLS and restrained refinement using *Refmac*5^52^, followed by rebuilding the model to the 2F_o_–F_c_ and the F_o_–F_c_ maps using Coot^53^. Refinement statistics are summarized in Supplementary Table 1.

### Malachite green colorimetric assay

The enzymatic activity assay was modified from Seamon and Stivers (2015)^54^. All assays were performed with purified wild-type SAMHD1 (residues 113–626) at 25 °C in a reaction buffer containing 50 mM Tris-HCl pH 8, 150 mM NaCl, 5 mM MgCl_2_, and 0.5 mM TCEP. Each 40 µL reaction, containing 10 µM pyrophosphatase, 0.5 µM SAMHD1, and 125 µM substrate or allosteric activator was quenched with 40 µL 20 mM EDTA after 15 min. Then, 20 µL Malachite green reagent was added to the solution and developed for 15 min before the absorbance at 650 nm was measured.

### Cell Lines and primary AML blasts

The human AML cell lines THP-1, OCI-AML2, OCI-AML3, Molm13, PL-21, HL-60, MV4-11, SIG-M5, ML2, NB4, KG1, MonoMac6, and HEL were obtained from DSMZ (Deutsche Sammlung von Mikroorganismen und Zellkulturen GmbH). THP-1 cells deficient in SAMHD1 (THP-1 KO) and control cells (THP-1 Ctr.) were generated using a CRISPR/Cas9 approach^55^.

THP-1 cells were plated at a density of 2 × 10^5^ cells per ml. After 24 h, 2.5 × 10^6^ cells were resuspended in 250 µl Opti-MEM, mixed with 5 µg CRISPR/Cas plasmid DNA, and electroporated in a 4-mm cuvette using an exponential pulse at 250 V and 950 mF utilizing a Gene Pulser electroporation device (Bio-Rad Laboratories). We used a plasmid encoding a CMV-mCherry-Cas9 expression cassette and a human SAMHD1 gene specific gRNA driven by the U6 promoter. An early coding exon of the SAMHD1 gene was targeted using the following gRNA construct: 5′-CGGAAGGGGTGTTTGAGGGG-3′. Cells were allowed to recover for 2 days in 6-well plates filled with 4 ml medium per well before being FACS sorted for mCherry-expression on a BD FACS Aria III (BD Biosciences). For subsequent limiting dilution cloning, cells were plated at a density of 5, 10, or 20 cells per well of nine round-bottom 96-well plates and grown for 2 weeks. Plates were scanned for absorption at 600 nm and growing clones were identified using custom software and picked and duplicated by a Biomek FXp (Beckman Coulter) liquid handling system.

For shRNA-mediated silencing of SAMHD1, OCI-AML3 cells were transduced by spinoculation with VSV-G pseudotyped lentiviral vectors carrying either pLKO.1-puro-control-shRNA or pLKO.1-puro-SAMHD1-shRNA^56^.

OCI-AML3 stable cell lines were generated by transduction with lentiviral vectors encoding unspecific shRNA (pLKO-*nontarget*) or shRNA specific to SAMHD1 (shSAMHD1 TRCN0000343807: sequence: *CCGGCCCTGAAGAAGATATTTGCTTCTCGAGAAGCAAATATCTTCTTCAGGGTTTTTG*) (Sigma) and selected with puromycin.

The HEL SAMHD1-WT and HEL SAMHD1-D311A cell lines were generated by co-transfection of the packaging vector pPAX2 (Addgene), either pHR-SAMHD1-WT or pHR-SAMHD1-D311A and a plasmid encoding VSV-G^57^. DAC- or AZA-resistant cell sublines were established by the continuous exposure of parental (DAC/AZA sensitive) HL-60 cell line to increasing drug concentrations^19^, and are part of the Resistant Cancer Cell Line (RCCL) collection (http://www.kent.ac.uk/stms/cmp/RCCL/RCCLabout). Briefly, cells were cultured at increasing AZA or DAC concentrations starting with the concentrations that inhibited viability of the parental cell lines by 50% (IC_50_). HMA concentrations were doubled every 2–6 weeks until cells readily grew in the presence of 10 µM and 1 µM AZA or DAC, respectively.

All cell lines were routinely tested for Mycoplasma, using the MycoAlert PLUS assay kit from Lonza, and were authenticated by short tandem repeat profiling, as described elsewhere^58^.

Mononuclear cells from blood or bone-marrow AML samples were purified by Ficoll-Hypaque gradient centrifugation. Leukemic cells were enriched by negative selection with a combination of CD3-, CD19- and CD235a-microbeads (all obtained from Miltenyi Biotec) according to the manufacturer’s instructions and separated by the autoMACS™ Pro Separator. All preparations were evaluated for purity, resulting in > 90% leukemic blasts.

All AML cell lines were cultured in IMDM (Biochrom) supplemented with 10% FBS (SIG-M5 20% FBS, Sigma-Aldrich), 4 mM L-glutamine (Sigma-Aldrich), 100 IU per ml penicillin (Sigma-Aldrich), and 100 mg per ml streptomycin (Sigma-Aldrich) at 37 °C in a humidified 5% CO_2_ incubator. AML blasts were cultivated in IMDM (Biochrom) supplemented with 10% FBS, 4 mM L-glutamine, 25 ng per ml hTPO, 50 ng per ml hSCF, 50 ng per ml hFlt3-Ligand, and 20 ng per ml hIL3 (all obtained from Miltenyi Biotec) in 5% CO_2_ and 37 °C.

### Manipulation of SAMHD1 through siRNA and Vpx-VLPs

For siRNA-mediated silencing, AML blasts (1.2 × 10^6^) were transfected with 2.5 µM ON-TARGET plus human SAMHD1 siRNA SMART-pool obtained from Dharmacon in resuspension electroporation buffer R (Invitrogen) using the Neon transfection system (Invitrogen) according to the manufacturer’s recommendation. In addition, ON-TARGET plus Non-targeting Pool (Dharmacon) was transfected in parallel. The electroporation was performed with one 20 ms pulse of 1700 V and analyzed 48 h after transfection by western blotting and a cell viability assay. The following siRNA duplexes were used: non-targeting (UGGUUUACAUGUCGACUAA; UGGUUUACAUGUUGUGUGA; UGGUUUACAUGUUUUCUGA; UGGUUUACAUGUUUUCCUA), SAMHD1 (GACAAUGAGUUGCGUAUUU; CAUGUUUGAUGGACGAUUU; AAGUAUUGCUAGACGUGAA; UUAGUUAUAUCCAGCGAUU)^19^. AML cell lines and primary AML blasts were spinoculated with VSV-G pseudotyped VLPs carrying either Vpx or Vpr from SIVmac251^19^. Briefly, VLPs, carrying either Vpx or Vpr from SIV_mac251_, were produced by co-transfection of 293T cells with pSIV3 + gag pol expression plasmids and a plasmid encoding VSV-G. The SAMHD1 degradation capacity of Vpx-VLPs was determined in THP-1 cells 24-h post transduction by intracellular SAMHD1 staining.

### Compounds

5-Aza-2′-deoxycytidine (DAC) and B02 was purchased from Sigma-Aldrich, 5-azacytidine (AZA), Ara-C, and Daunorubicin from Tocris, Melphalan from Aspen, Topotecan from Novartis, Etoposide from Teva GmbH, and Lomustine was obtained from Santa Cruz. SGI-110 and dNs were obtained from Sigma-Aldrich^59^. All nucleotide standards and internal standards for the LC–MS/MS analysis were obtained from Sigma-Aldrich, Silantes or Alsachim. Labeled 5-azacytidine and 5-aza-2′-deoxycytidine, 5-azacytidine-^15^N_4_ and 5-aza-2′-deoxycytidine-^15^N_4_, for LC–MS/MS analysis were manufactured by Toronto Research Chemicals.

### Cell viability assay

The viability of AML cell lines treated with various drug concentrations was determined by the 3-(4,5-dimethylthiazol-2-yl)-2,5-diphenyltetrazolium bromide (MTT) dye reduction assay^60^. For AML blasts ex vivo endpoint viability assays were performed using the CellTiter-Glo (Promega) assay according to the manufacturer’s protocol. The amount of ATP is directly proportional to the number of cells present in culture. Briefly, cells were seeded at 5000 cells per well (10,000 cells per well for MTT dye) in 96-well plates and treated for 96 h with compounds, over a range of concentrations. Then the assay was terminated and luminescence was measured on a Tecan infinite M200 (TECAN) or for MTT dye at a wavelength of 560 nm (reference wavelength 620 nm). IC_50_ values were calculated by using CalcuSyn from Biosoft.

### Flow cytometry

The intracellular SAMHD1 staining was performed as previously described. Staining for surface markers (CD33, CD34, CD45) for AML blasts was applied before fixation. The following fluorochrome-conjugated antibodies were used: CD33-PE and CD34-FITC, both from Miltenyi Biotech, CD45-V450 from BD Pharmingen, all diluted 1:11 per 1 × 10^7^ cells, and Alexa-Fluor-660 from Invitrogen, Life Technologies, 1:200). Samples were analyzed by using a FACSVerse or FACSCanto II flow cytometer from BD Biosciences and the FlowJo software from TreeStar.

### Western blotting

For western blotting^19^, cells were lysed in Triton X-100 sample buffer and proteins separated by sodium dodecyl sulfate–polyacrylamide gel electrophoresis. Proteins were blotted on a nitrocellulose membrane (Thermo Scientific).

The following primary antibodies were used at the indicated dilutions: SAMHD1 (Proteintech, 1:1000), β-actin (BioVision via BioCat, 1:2000), DCK (Santa Cruz, 1:500), CDA (Santa Cruz, 1:100), ENT1 (Abcam, 1:500), and UCK1 (Thermo Scientific, 1:1000), UCK2 (Thermo Scientific, 1:1000), RRM1 (Santa Cruz, 1:1000), RRM2 (Santa Cruz, 1:1000), OCTN1 (Abnova, 1:1000), cPARP (,1:2000), yH2AX (Cell Signaling, 1:2000), Chk2 (Cell Signaling, 1:1000), pChk2 (Cell Signaling, 1:1000), SAMHD1 (ProteinTech, 1:2000), TIF1β (Cell Signalling, 1:1000), pTIF1β (Cell Signaling, 1:1000), DNMT1 (Active Motif, 1:1000). Visualization and quantification were performed by using peroxidase-labeled secondary antibodies (Calbiochem) and enhanced chemiluminescence (SuperSignal West FEMTO Substrate; Thermo Scientific) or IRDye-labeled secondary antibodies (LI-COR Biotechnology) according to the manufacturer’s instructions. Band volume analysis was conducted by Odyssey LICOR. Uncropped scans of the blots are provided as Source Data file.

### mRNA analyses

RNA extraction and TaqMan-based mRNA quantification of SAMHD1 (assay no. Hs00210019_m1) and RNaseP (TaqMan® RNase P Control Reagents Kit (4316844)) as endogenous reference control were performed according to the manufactures protocol (Applied Biosystems)^61^. Total RNA was extracted using the RNeasy Kit from Qiagen and stored at −80 °C until use. Relative quantitative PCR analyses were performed on the ABI Prism 7500 sequence detection system (Applied Biosystems). SAMHD1 mRNA expression levels were quantified by using the ΔΔCt method with RNaseP mRNA as an endogenous reference control. All samples were run in triplicate. Data analysis was conducted using the 7500 System Software (Applied Biosystems).

### LC–MS/MS analysis

AML cells were seeded at 5 × 10^5^ cells per well in 24-well plates, treated with labeled ^15^N_4_-AZA or ^15^N_4_-DAC and incubated at 37 °C in a humidified 5% CO_2_ incubator for 6 h. Subsequently, cells were washed twice in 1 ml PBS, pelleted and stored at −80 °C until measurement. The concentrations of dNTPs, ^15^N_4_-AZA-TP, and ^15^N_4_-DAC-TP in the samples were analyzed by liquid chromatography–electrospray ionization–tandem mass spectrometry^62^. Briefly, the analytes were extracted by protein precipitation with methanol. An anion-exchange HPLC column (BioBasic AX, 150 × 2.1 mm, 5 µM, Thermo Scientific) was used for the chromatographic separation and a 5500 QTrap (Sciex) was used as analyzer, operating as triple quadrupole in positive multiple reaction monitoring (MRM) mode. The analysis of the dNTP was performed as previously described^62^. In addition, ^15^N_4_-AZA-TP or ^15^N_4_-DAC-TP were quantified using cytidine-^13^C_9_, ^15^N_3_-triphosphate (^13^C_9_,^15^N_3_-CTP) or 2-deoxycytidine-^13^C_9_,^15^N_3_-triphosphate (^13^C_9_,^15^N_3_-dCTP) as internal standard (IS). The precursor-to-product ion transitions used as quantifiers were *m*/*z* 473.1 → 117.0 for ^15^N_4_-DAC-TP and *m*/*z* 489.0 → 117.1 for ^15^N_4_-AZA-TP. Owing to the lack of commercially available standards for ^15^N_4_-AZA-TP and ^15^N_4_-DAC-TP, relative quantification was performed by comparing the peak area ratios (analyte/IS) of the differently treated samples.

### Apoptosis assay

Sub-G1 cells as a marker for DNA fragmentation in late apoptotic cells were measured according to Nicoletti by flow cytometry^63^. Briefly, Aza- or DAC-treated and untreated cells were washed once in 1× PBS, incubated for at least 2 h at 4 °C with Nicoletti buffer (0.1% trisodiumcitrate-dihydrate pH 7.4, 0.1% Triton X-100, 50 µg/ml propidium iodide), and diluted before measurement in 1× PBS. Samples were analyzed with a FACSVerse (BD Biosciences) and FlowJo software (TreeStar).

### Global DNA methylation assay

For the detection of differences in global DNA methylation, the ELISA-based global DNA methylation—LINE-1 kit from Active Motif was used. The assay was performed in triplicates using DNA samples from THP-1 KO and THP-1 control cells after treatment with AZA or DAC according to the manufacturer’s instructions. The colorimetric readout was quantified on a spectrophotometer (Tecan infinite M200, TECAN) at 450 nm with a reference wavelength of 655 nm.

### DNA methylation analyses of the SAMHD1 promoter

*SAMHD1* promoter contains five *HpaII* sites surrounding the transcription start site^40^. Methylation of the *HpaII* sites in the *SAMHD1* promoter would prevent digestion by the HpaII, and the intact sequence would serve as a template for PCR amplification using *SAMHD1* promoter-specific primers that flank the *HpaII* sites. To measure methylation of the *SAMHD1* promoter genomic DNA was treated with the methylation-sensitive HpaII endonuclease or left untreated as described previously with some modifications^40^. PM3.fwd: TTCCGCCTCATTCGTCCTTG and PM3.rev: GGTTCTCGGGCTGTCATCG were used as SAMHD1 promoter-specific primers. A single PCR product (993-bp) corresponding to the *SAMHD1* promoter sequence was obtained from untreated genomic DNA and treated DNA from cells with methylated but not from cells with unmethylated *SAMHD1* promoter. To serve as input control, a 0.25-kb fragment of the *GAPDH* gene lacking *HpaII* sites was PCR-amplified using the same template DNA^40^.

### Mice

Female non-obese diabetic severe combined immunodeficient gamma (NSG) mice were purchased from Jackson ImmunoResearch laboratories (Ban Harbor, ME). All mice used in the experiments were between 6 and 10 weeks of age. All animal experiments were performed according to the regulations of the United Kingdom Home Office and German authorities.

### Xenograft mouse model

In total, 0.2 × 10^6^ THP-1 or HEL cells were intravenously injected into NSG mice through the tail vein as described elsewhere^64^. Ten days after leukemia cell injection, the mice were given either vehicle control or decitabine (0.4 mg per kg, i.p., twice a week) for 4 weeks or azacytidine (either 5 mg per kg, i.v., twice a week for 4 weeks, or 2.5 mg per kg, 5× per week with 1 week drug pause between weeks 2 and 3). Mice were monitored closely for clinical signs of leukemia such as weight loss, hind-limb paralysis. Blood was drawn for blood-counts analysis to confirm leukemia.

### Patients

Patients were admitted to the Frankfurt and Münster University Hospitals between 2012 and 2017 and were treated for newly diagnosed AML with regimens containing standard dose decitabine or azacytidine. In addition, viable AML cells were purified from the bone marrow of patients who were admitted to the University Hospital Frankfurt in 2016 and 2017. Patients at the Frankfurt and Münster University Hospitals are routinely advised to undergo a bone-marrow biopsy at diagnosis. All patients consented to the scientific analyses of their data and of biomaterial obtained for diagnostic purposes. All patients gave informed consent according to the Declaration of Helsinki to participate in the collection of samples. The use of whole blood and bone marrow aspirates was approved by the Ethics Committee of Frankfurt University Hospital (approval no. SHN-11-2016 and SHN-03-2017) and University Hospital Muenster (approval no. 2007-390-f-S). For the analyses, patient records were reviewed by physicians who were unaware of the SAMHD1 expression results in the diagnostic biopsies. Remission criteria and cytogenetic risk groups were assessed according to the ELN guidelines. The best response to DAC or AZA therapy was analyzed in bone-marrow biopsies and aspirates and defined as complete (CR) if the blast count was < 5%, and as “no CR” if the blast count was > 5%. Material is available on request.

### Immunostaining of bone marrow samples

Tissues were fixed in 4% buffered formalin, descaled by EDTA and embedded in paraffin. For immunohistochemical staining^19^. Two micrometers of bone marrow tissue sections were incubated with EnVision Flex Target Retrieval Solution, pH low (K8005, DAKO) and stained with primary antibodies directed against SAMHD1 (12586-1-AP, Proteintech, 1:3000) and against CD34 (IR632, DAKO) for 40 min at room temperature. Polymeric secondary antibodies coupled to HRPO peroxidase and DAB were used for visualization (REAL EnVision Peroxidase/DAB +, K5007, DAKO). Tissue samples were analyzed by light microscopy after counterstaining with Meyer’s hematoxylin (K8008, DAKO). Two pathologists, who were blinded to clinical history and therapeutic response, independently scored the SAMHD1 IHCs. They evaluated all tissue sections for nuclear SAMHD1 staining using a four-stage staining score: 0 = negative; 1 = weak intensity of staining; 2 = strong intensity of staining in <25% of blasts; and 3 = strong intensity of staining in more than 25% of blasts. IHC staining scores of 0 and 1 were defined as ‘no or low expression’ and IHC staining scores of 2 and 3 were defined as ‘high SAMHD1 expression’. Membranous CD34 staining for the quantification of the number of AML blasts was evaluated using a two-stage staining score: 0 = negative; 1 = positive.

### Statistics

Statistical data analysis was performed in R, version 3.3.2. Population means were compared using Student’s *t*-test if data were approximately normally distributed or the rank-sum test as a non-parametric alternative otherwise. Survival analyses were performed using the Kaplan–Meier estimator, assessing statistical significance of survival differences using the logrank test. Pearson’s correlation coefficient was used to compute correlations between variables, using a *t*-test to assess significance of the correlation. Dose–response curves were analyzed using the *drm* package in R, using the logistic and log-logistic models. A likelihood-ratio (chi-squared) test was used to assess model significance and goodness of fit.

### Reporting summary

Further information on research design is available in the Nature Research Reporting Summary linked to this article.

## Supplementary information

Supplementary Information

Description of Additional Supplementary Files

Supplementary Dataset 1

Supplementary Dataset 2

Reporting Summary

Source Data

## Data Availability

The atomic coordinates and structure factors have been deposited in the Protein Data Bank, www.pdb.org (PDB ID code 6CM2). The source data underlying Figs 3–6 and Supplementary Figs 3–27 are provided as a Source Data file. All the other data supporting the findings of this study are available within the article and its supplementary information files and from the corresponding author upon reasonable request. A reporting summary for this article is available as a Supplementary Information file.
