# Supplementary material for: Selective inactivation of hypomethylating agents by SAMHD1 provides a rationale for therapeutic stratification in AML
Source: Nat Commun. 2019 Aug 2;10:3475. doi: 10.1038/s41467-019-11413-4 (PMC6677770; doi:10.1038/s41467-019-11413-4)
Supplement: Supplementary file 1 — Supplementary Information [file 41467_2019_11413_MOESM1_ESM.pdf]

Supplementary Information

Selective Inactivation of Hypomethylating Agents by SAMHD1 Provides a Rationale for Therapeutic Stratification in AML

Oellerich and Schneider et al.

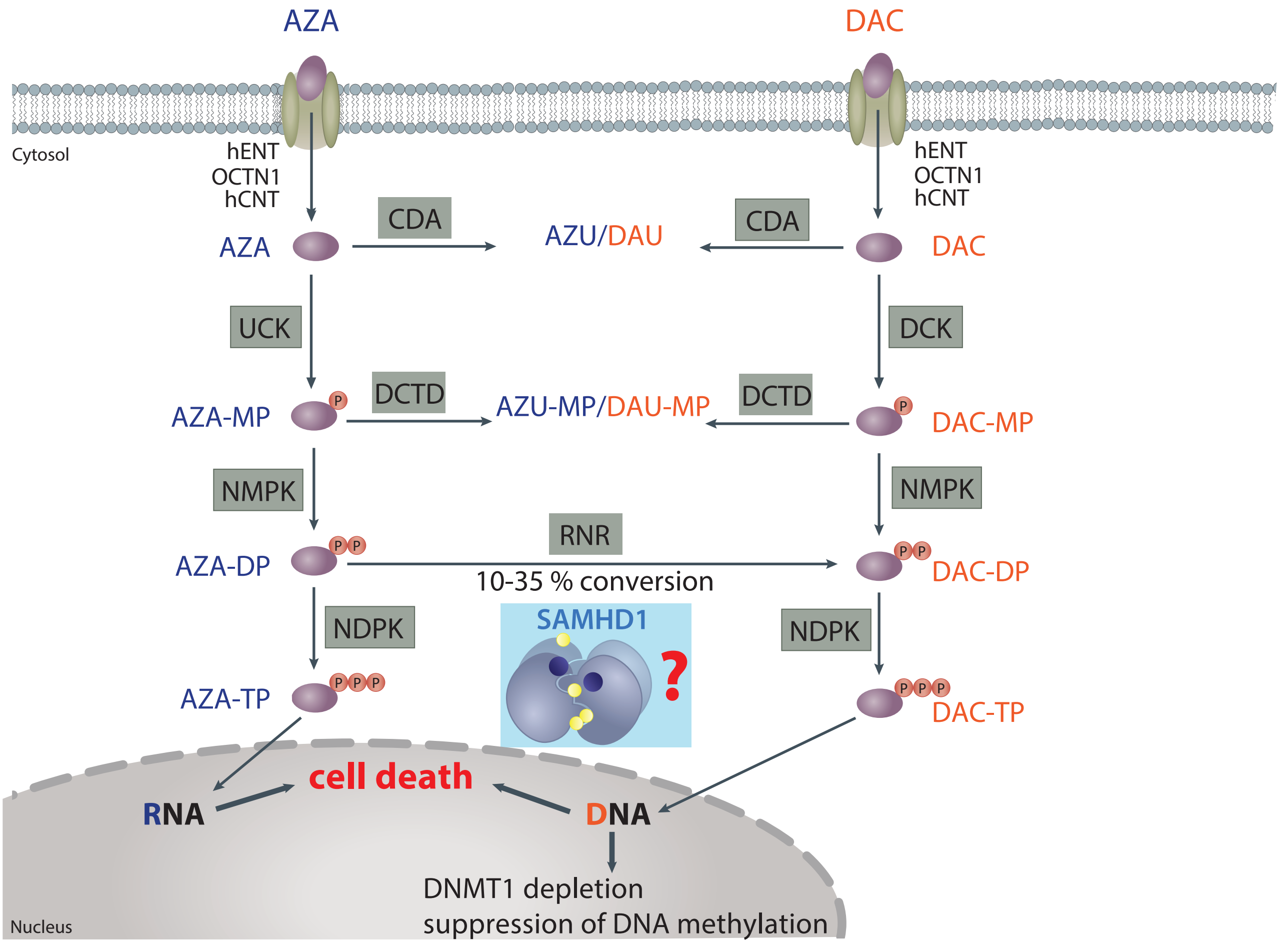

Supplementary Figure 1. Metabolic pathway of DAC and AZA. After their cellular uptake by nucleoside-specific transporters such as the equilibrative nucleosid transporter (SLC29A1; hENT1), ergothioneine transporter OCTN1 (SLC22A4; ETT), or human concentrative nucleoside transporter (SLC28; hCNT), AZA and DAC are activated and metabolically converted into the active nucleotides AZA-triphosphate (AZA-TP) and DAC-TP by sequential phosphorylation events. A rate-limiting step during activation of AZA and DAC is their phosphorylation to monophosphate derivatives by uridine-cytidine kinase (UCK) and deoxycytidine-kinase (DCK), respectively. The resulting monophosphorylated azanucleoside forms are subsequently phosphorylated by pyrimidine monophosphate and diphosphate kinases (nucleoside monophosphate kinase (NMPK) and nucleoside-diphosphate kinase (NDPK), respectively), resulting in diphosphate and triphosphate derivatives. AZA and DAC or their monophosphates AZA-MP and DAC-MP may be also inactivated by deamination to azauridine (AZU) or deazauridine (DAU) or AZU-MP and DAU-MP through activity of cytidine deaminase (CDA) or deoxycytidilate deaminase (DCTD), respectively. Of note, ~10-35 % of AZA were reported to feed into the metabolic pathway of DAC, possibly following the intracellular reduction of AZA diphosphate to DAC diphosphate by ribonucleotide reductase (RNR) (Figure created by Dr. Alessia Ruggieri, University of Heidelberg.).

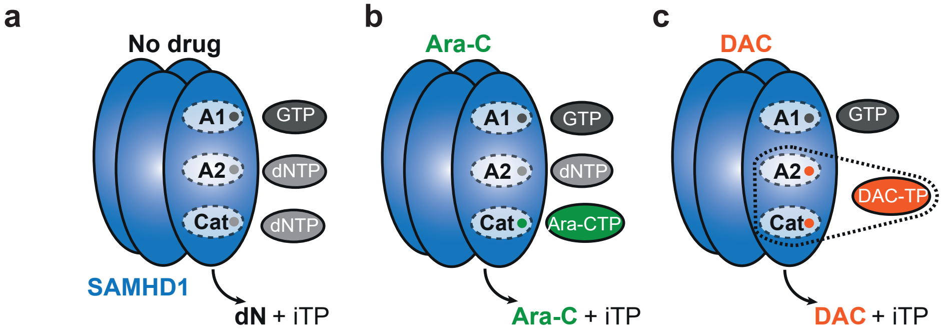

Supplementary Figure 2. SAMHD1 activity in the context of NTP, dNTP and nucleoside analog triphospahtes. The triphosphohydrolase activity of SAMHD1 requires the assembly of a homo-tetramer complex, which is regulated by binding of GTP (or dGTP) to the allosteric site 1 (A1) and any canoical dNTP to the allosteric site 2 (A2) resulting in the hydrolysis of dNTPs in 2´-deoxynucleosides and inorganic triphosphate (a). Nucleoside analog triphosphates with modifications at the 2´sugar position (e.g. arabinose-based Ara-CTP), which interacts only with the catalytic site, require the binding of any canonical dNTP to allosterich site 2 to induce SAMHD1 tetramerization and activity (b). 2´-deoxyribose-based nucleoside analog triphosphate DAC-TP binds to both the allosteric A2 and the catalytic site of SAMHD1, being both a substrate and an activator of the enzyme (c) (Figure created by Federico Comoglio).

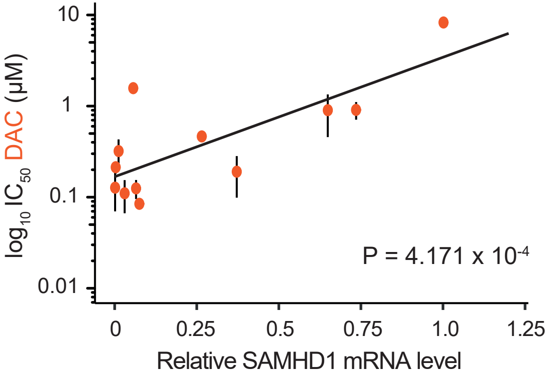

Supplementary Figure 3. SAMHD1 mRNA expression correlates with DAC cytotoxicity. qPCR-based analyses of SAMHD1 mRNA expression were correlated with DAC IC50 values (data taken from Figure 2b and Supplementary Table 2) in AML cell lines. Closed circles and error bars represent mean ± s.d. of three independent experiments, each was performed with 3 technical replicates. Data were analyzed using a generalized log-logistic model (Likelihood-Ratio / Chi-square test) (Source data are provided as Source Data file).

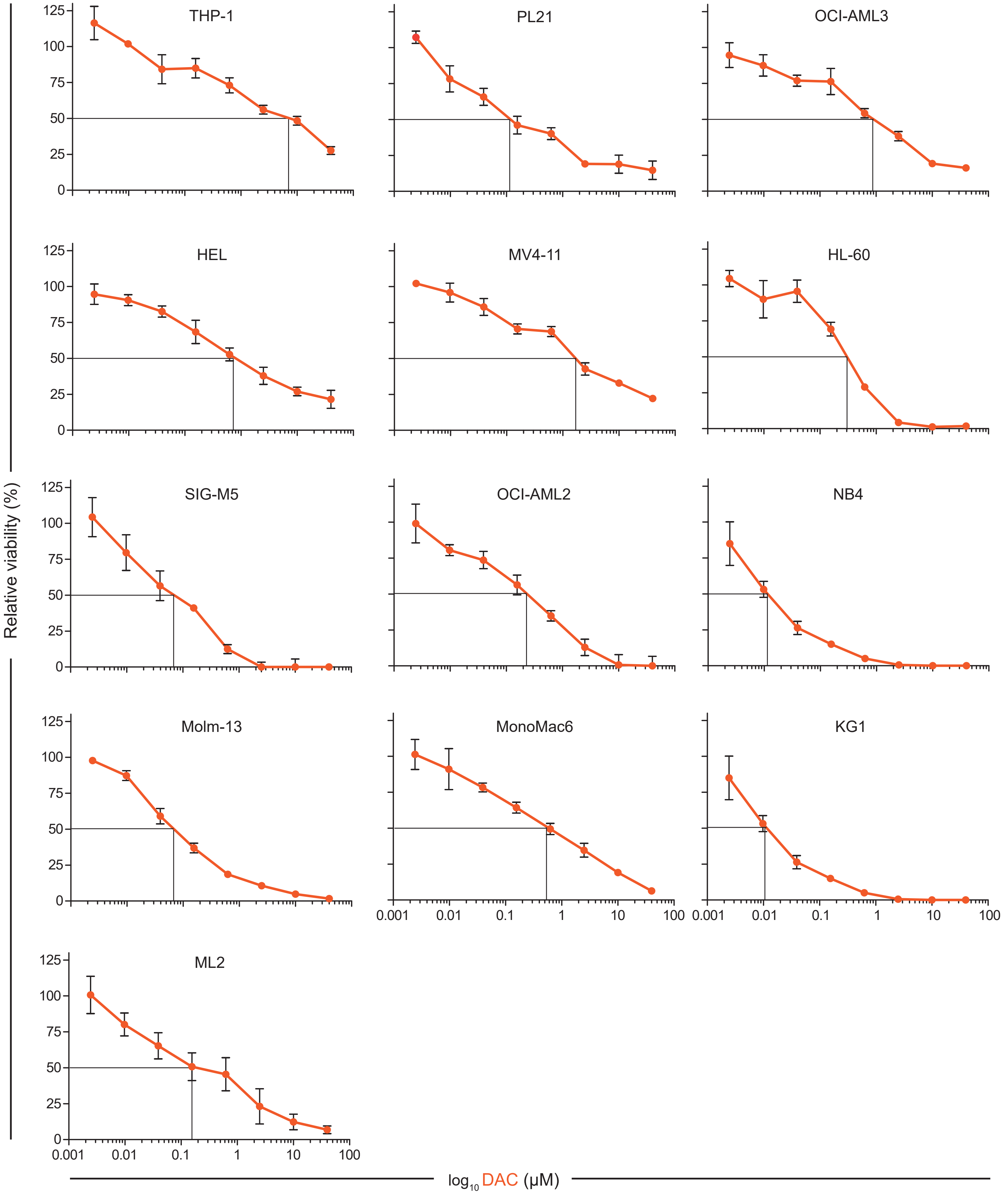

Supplementary Figure 4. Dose-response cytotoxicity of DAC in AML cell lines. 13 AML cell lines were treated with diffenent concentrations of DAC for 96 h before viability was quantified by MTT analyses. Values are means ± s.d. of triplicates of one representative experiment. Three independent experiments were performed. The x-axis intersection of additional lines indicates the IC50 value for each cell line (Source data are provided as Source Data file).

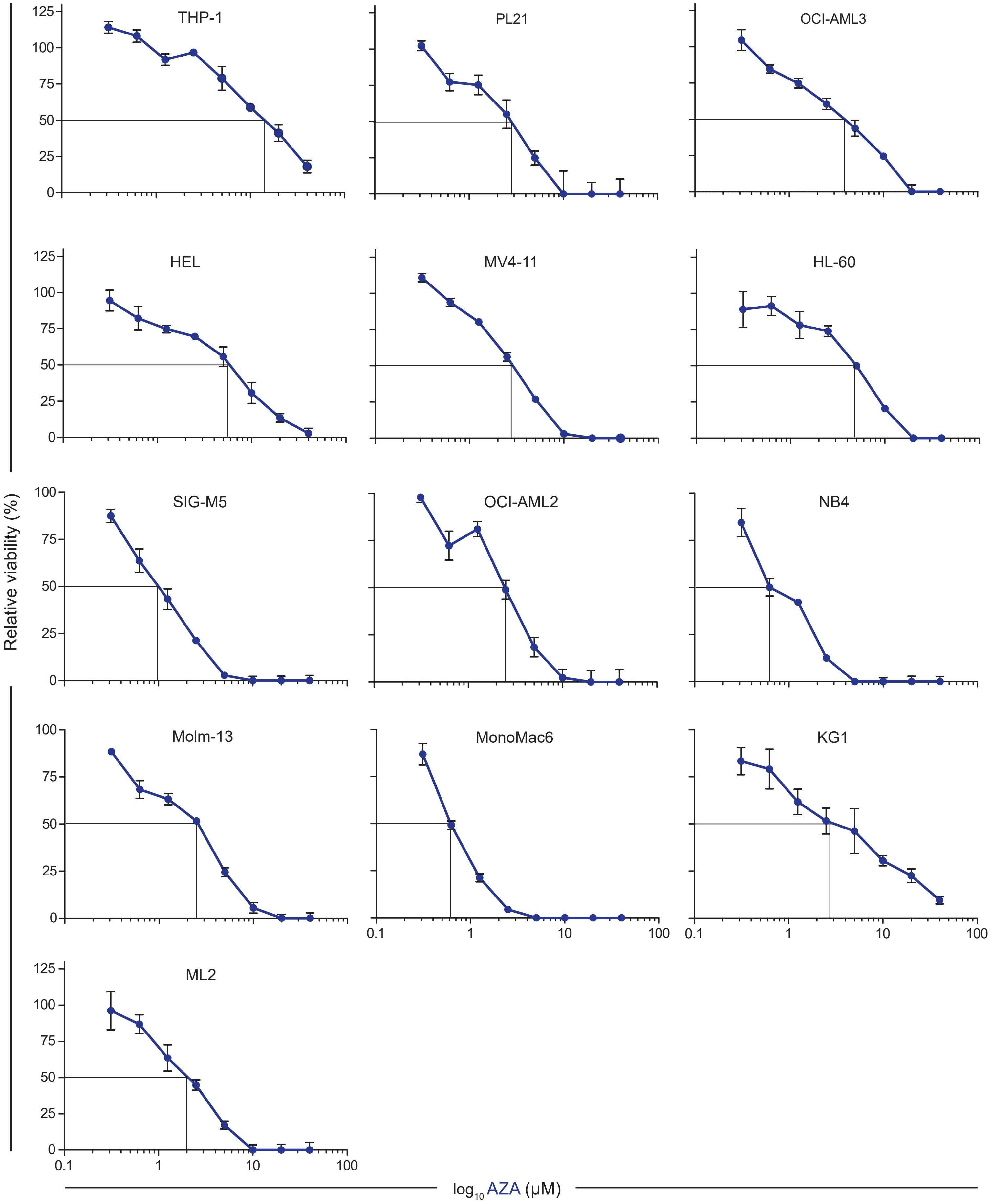

Supplementary Figure 5. Dose-response cytotoxicity of AZA in AML cell lines. 13 AML cell lines were treated with diffenent concentrations of AZA for 96 h before viability was quantified by MTT analyses. Values are means ± s.d. of triplicates of one representative experiment. Three independent experiments were performed. The x-axis intersection of additional lines indicates the IC50 value for each cell line (Source data are provided as Source Data file).

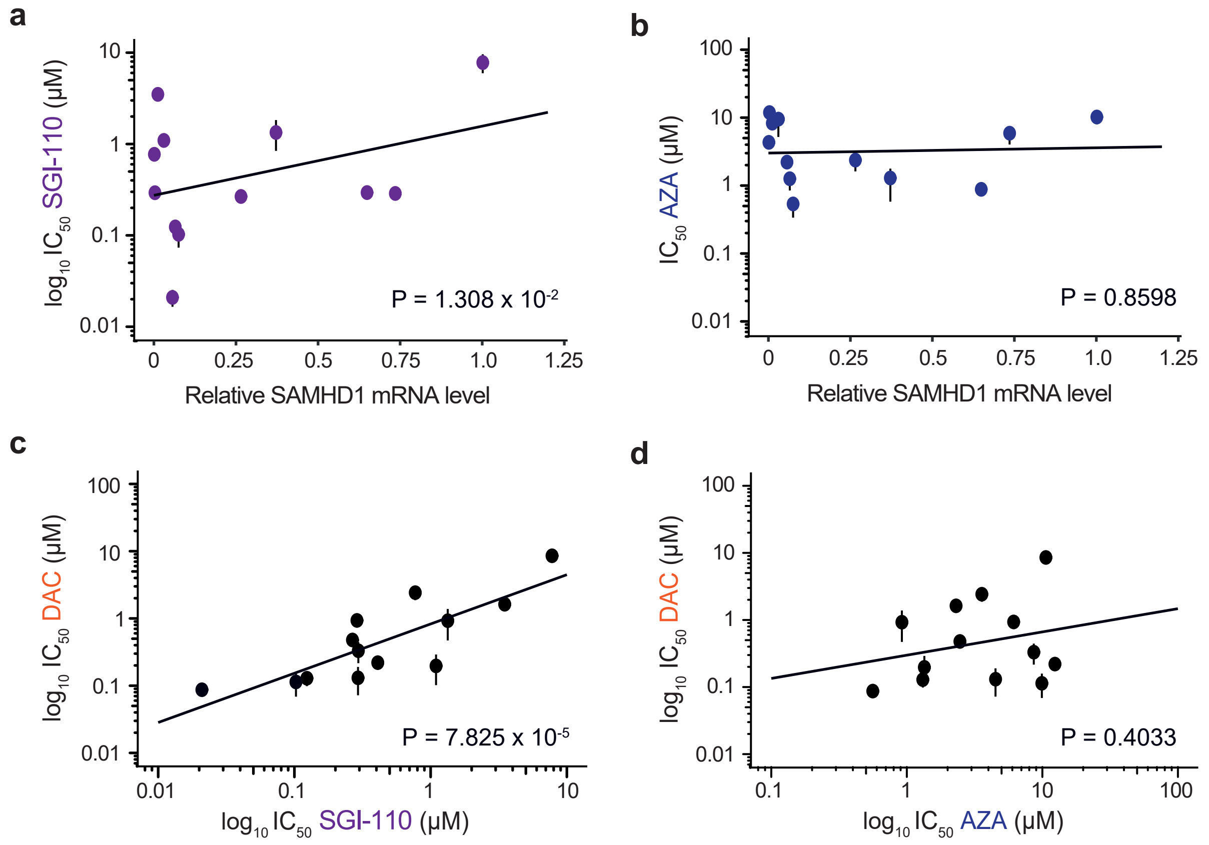

Supplementary Figure 6. SAMHD1 mRNA expression correlates with the cytotoxicity of SGI-110. (a and b) qPCR-based analyses of SAMHD1 mRNA expression were correlated with IC50 values of a) SGI-110 (violet) or b) AZA (dark blue) (data taken from Fig. 2b and Supplementary Table 2) in AML cell lines. Blotting of DAC IC50 values against c) SGI-110 IC50 values showed statistical significance, in contrast to the correlation with IC50 values for SGI-110 and d) AZA. Closed circles and error bars represent mean ± s.d. of three independent experiment each was performed with technical replicates (n=3). Data were analyzed using a generalized log-logistic model (Likelihood Ration / Chi-square test) (Source data are provided as Source Data file).

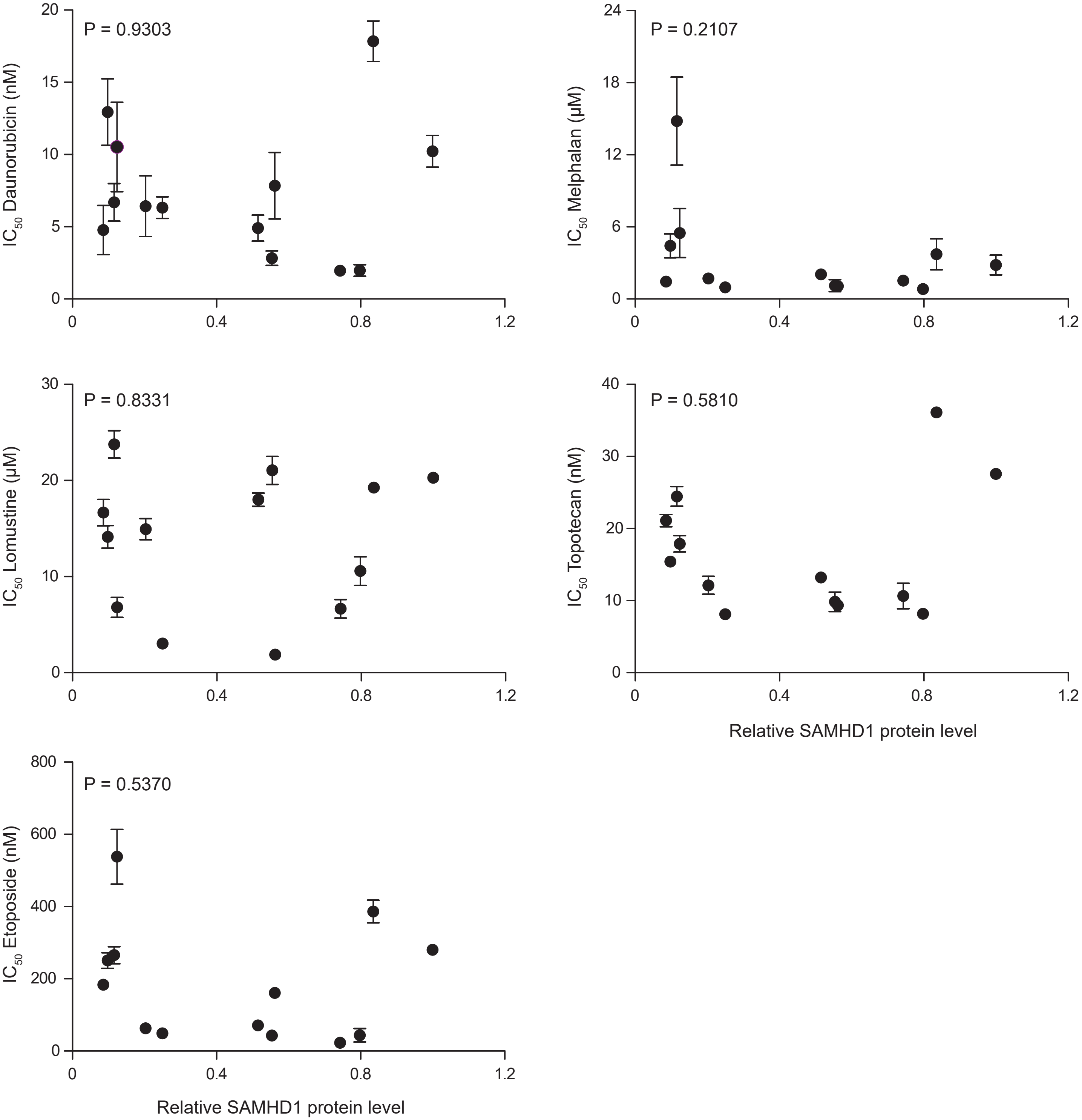

Supplementary Figure 7. SAMHD1 protein levels do not correlate with cytotoxicity of several non-nucleoside cancer drugs. Correlation analyses for IC50 values of daunorubicin, lomustine, etoposide, melphalan or topotecan in 13 AML cell lines and cells´s relative protein expression levels of SAMHD1 (taken from Figure 2a). Expression was normalized to β-actin and is shown as arbitrary units (a.u.); the relative SAMHD1 expression in THP-1 cells was set to 1. Closed circles and error bars represent means ± s.d. of three independent experiments, each performed using 3 technical replicates. Data were analyzed using a generalized log-logistic model. P-values for goodness-of-fit were computed using likelihood-ratio (chi-squared) test (Source data are provided as Source Data file).

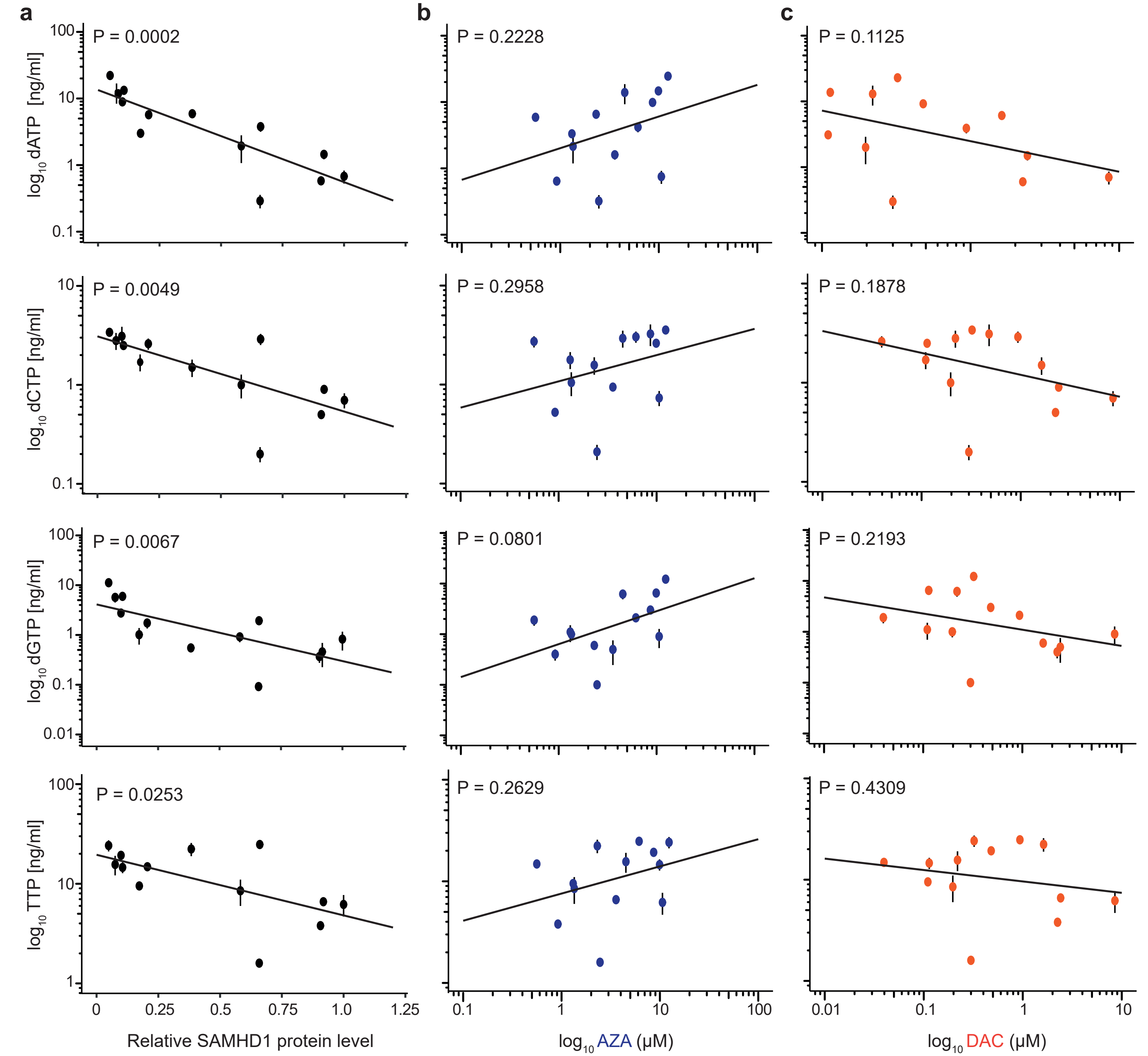

Supplementary Figure 8. Correlation analyses for dNTP levels with a) SAMHD1 expression, b) AZA-, or c) DAC IC50 values in AML cells. SAMHD1 expression levels were normalized to β-actin and are shown as arbitrary units (a.u.); the realtive SAMHD1 expression in THP-1 cells was set to 1. Closed circles and error bars represent means ± s.d. of three indenpendent experiments, each performed using 3 technical replicates. Data were analyzed using a generalized log-logistic model. P-values for goodness-of-fit were computed using the likelihood-ratio (chi-squared) test (Source data are provided as Source Data file).

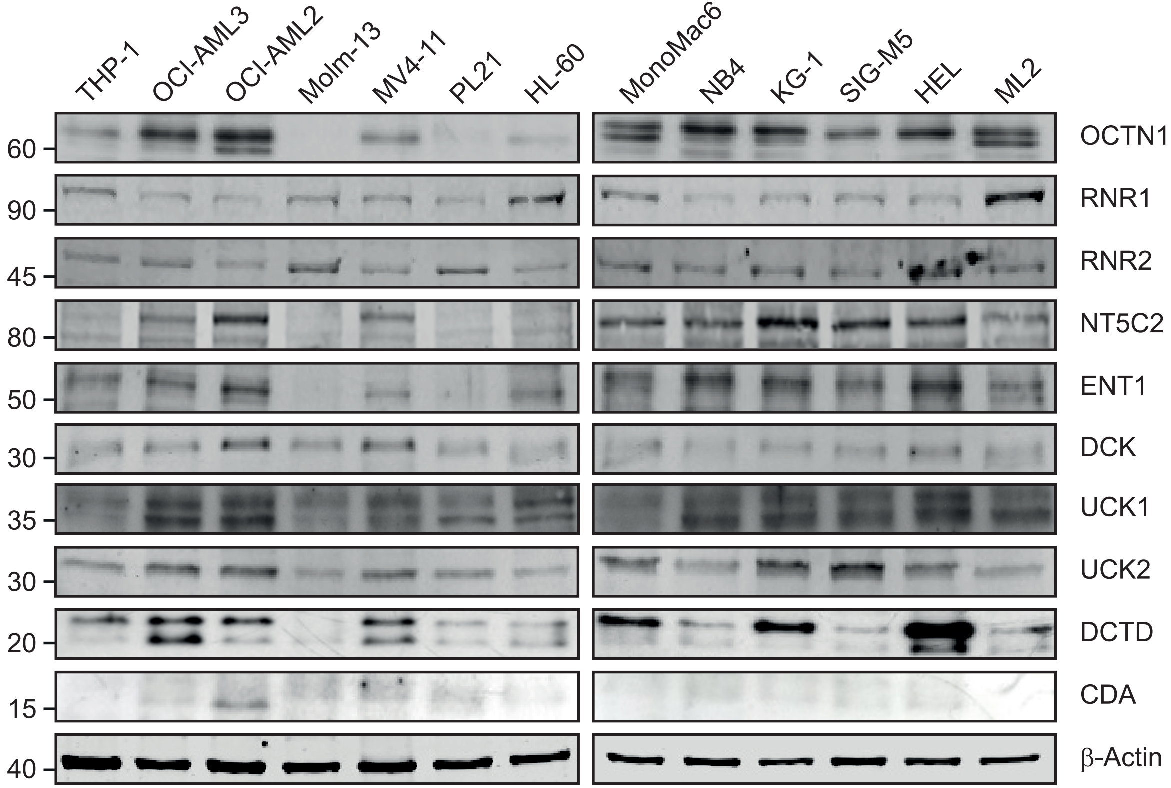

Supplementary Figure 9. Expression of proteins involved in nucleoside-analogue uptake and metabolism. Representative immunoblots of proteins previously reported to be involved in nucleosid analogue uptake and its conversion to the active metabolite, in the indicated AML cell lines. β-Actin served as a loading control. Three independent experiments were performed (Source data are provided as Source Data file).

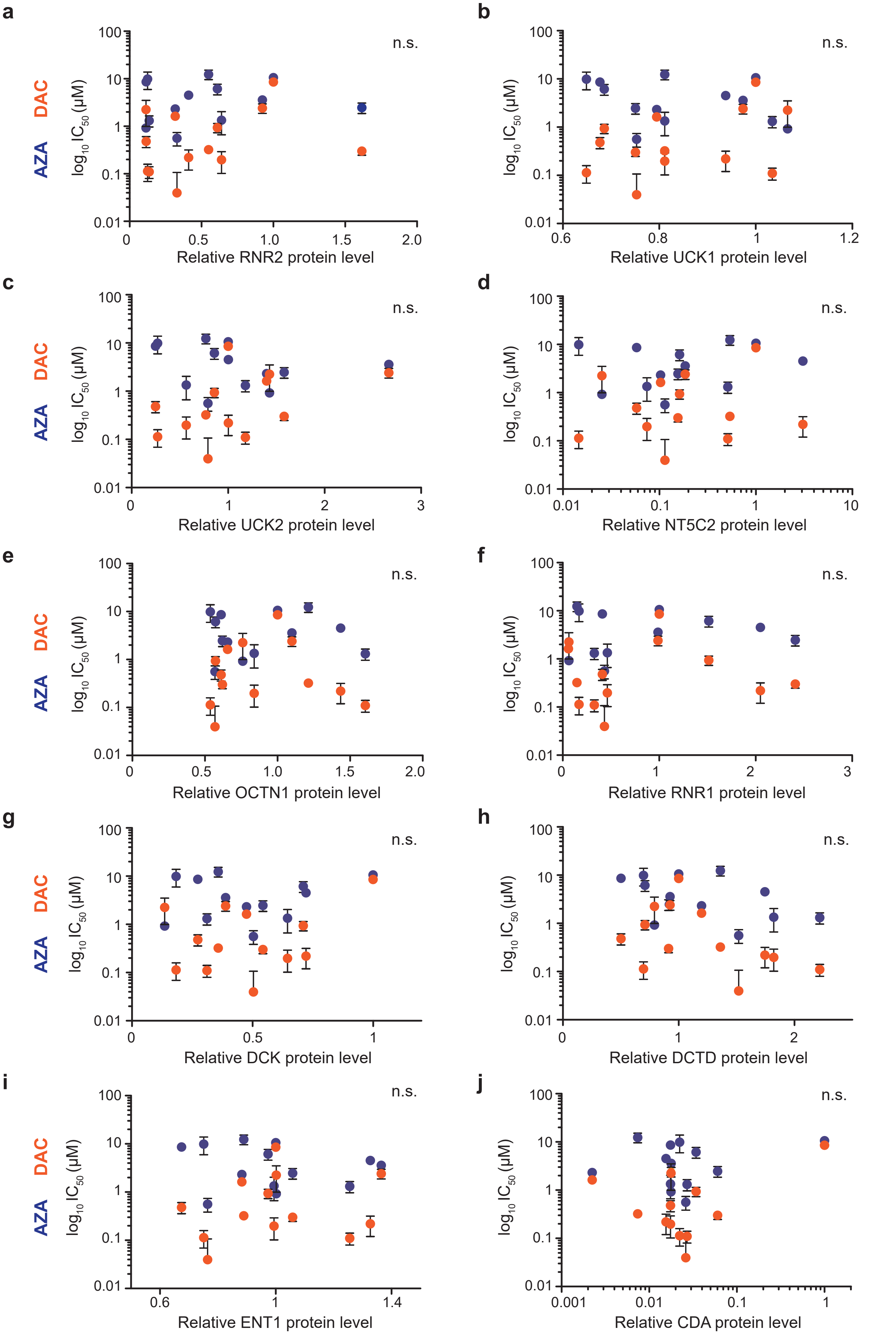

Supplementary Figure 10. Correlation analyses for AZA and DAC with proteins involved in nucleoside analogue metabolism. (a-j) correlation analyses between AZA (dark blue circles) and DAC (orange circles) IC50 values for AML cell lines shown in Figure 2b and Supplementary Figure 4 and the relative protein expression levels of a) RNR2, b) UCK1, c) UCK2, d) NT5C2, e) OCTN1, f) RNR1, g) DCK, h) DCTD, i) ENT1 or j) CDA. Expression levels were normalized to β-Actin and are shown as arbitrary units (a.u.); the relative expression of each protein in THP-1 cells was set to 1. Closed circles and error bars represent mean ± s.d. of three independent experiments each preformed in technical replicates (n=3, data taken from Supplementary Table 2). Data were analyzed using generalized logistic and log-logistic models, goodness of fit was assessed using the likelihood ratio / chi-squared test (Source data are provided as Source Data file).

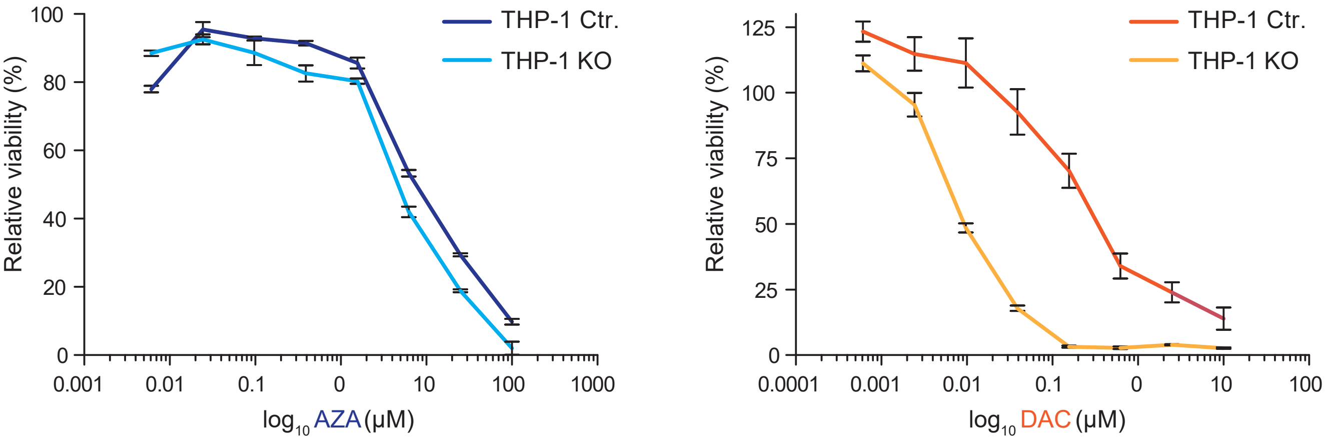

Supplementary Figure 11. Viability assay for THP-1 SAMHD1 KO and control cells. Dose-response curve of THP-1 Ctr. and THP-1 KO cells treated with different concentrations of azacytidine (AZA, blue lines, left panel) and decitabine (DAC, orange lines, right panel) incubated for 96 h before viability was quantified by MTT analyses. Values are mean ± s.d. of triplicates of one representative experiment. Three independent experiments were performed (Source data are provided as Source Data file).

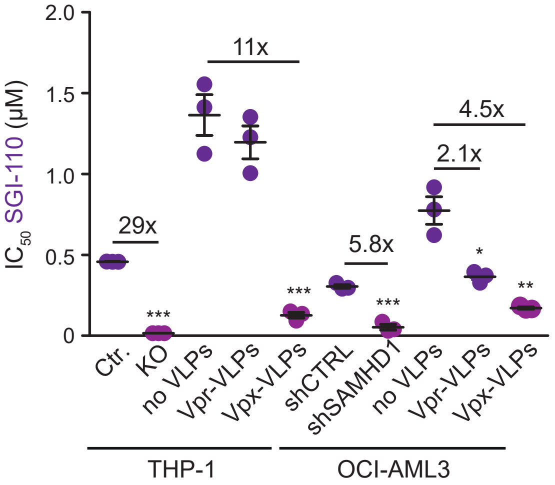

Supplementary Figure 12. SAMHD1 levels influence the cytotoxicity of SGI-110 in AML cells. Cytotoxicity of SGI-110 expressed as IC50 values were quantified in SAMHD1 knockout (THP-1 KO) and control (THP-1 Ctr.) THP-1 cells, SAMHD1-specific or control shRNA treated OCI-AML3 cells and THP-1 or OCI-AML3 cells exposed to either Vpx-VLPs or Vpr-VLPs (control), or left untreated (No VLPs). Horizontal lines and error bars represent mean ± s.d. of three independent experiment each was performed with technical replicates (n=3). Numbers above the dot plots indicate the change in IC50 values after manipulation of SAMHD1. Statistical analyses were performed using unpaired two-tailed Students' t-test. *$p \leq 0.05$; **$p < 0.01$; ***$p < 0.001$ (Source data are provided as Source Data file).

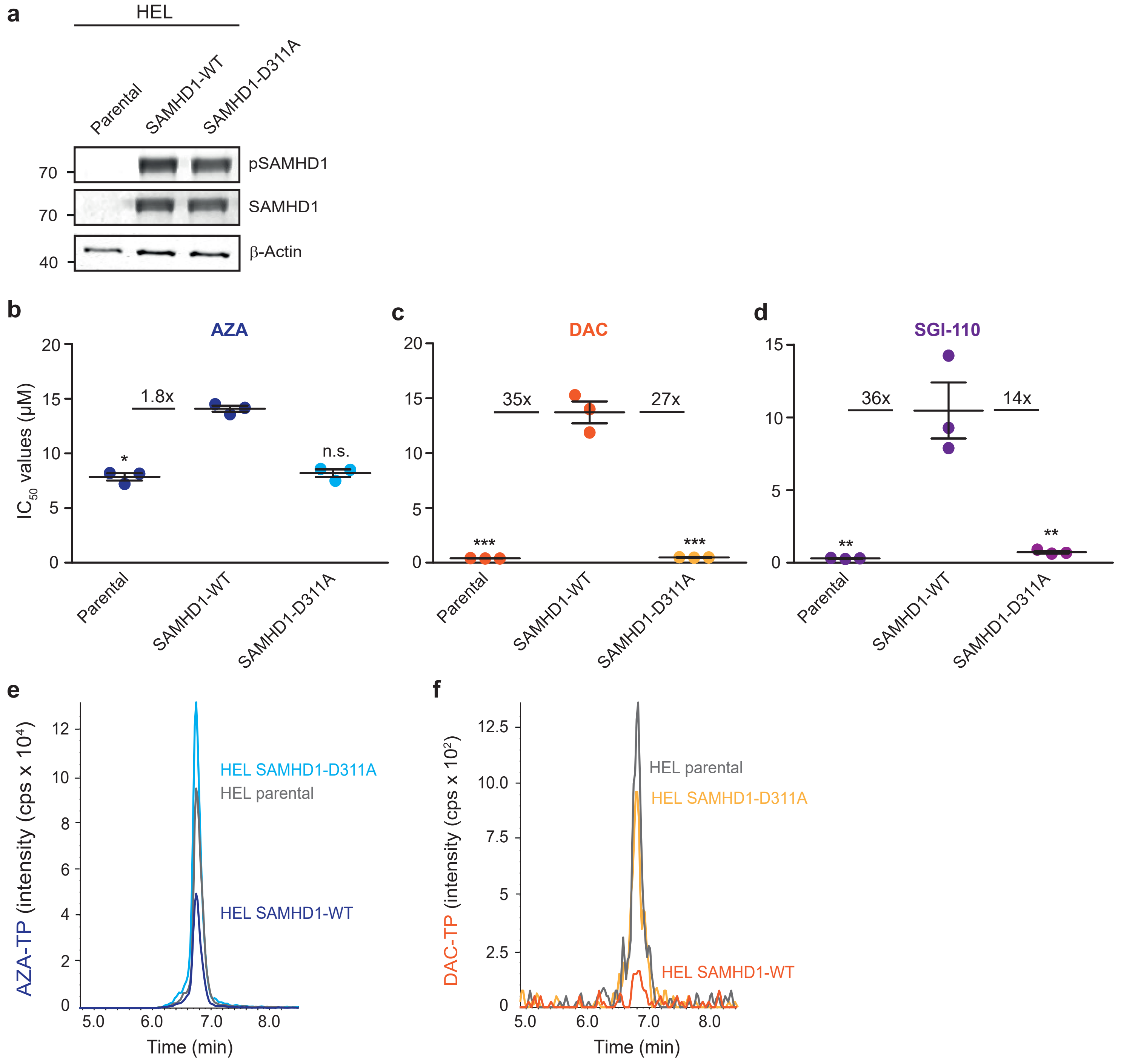

Supplementary Figure 13. Overexpression of catalytically active SAMHD1 reduces cytotoxicity of DAC and SGI-110, but not AZA. (a) SAMHD1 protein expression in parental HEL cells and HEL cells overexpressing either SAMHD1-WT or the dNTPase-defective SAMHD1-D311A mutant. β-Actin served as loading control. (b-d) cytotoxicity of (b) AZA, (c) DAC, or (d) SGI-110, expressed as IC50 values, were quantified for parental and SAMHD1-manipulated HEL cells. Horizontal lines and error bars represent mean ± s.d. of three independent experiment each was performed with technical replicates (n=3). Numbers indicate the change in IC50 values after manipulation of SAMHD1. Statistical analyses were performed using unpaired two-tailed Students' t-test. *p ≤ 0.05; **p < 0.01; ***p < 0.001. (e and f) representative LC-MS/MS chromatograms of (e) AZA-TP or (f) DAC-TP in parental HEL cells (grey) and HEL cells stably expressing either SAMHD1-WT (orange) or the SAMHD1-D311A mutant (yellow) treated with 10 µM for 6 h (Source data are provided as Source Data file).

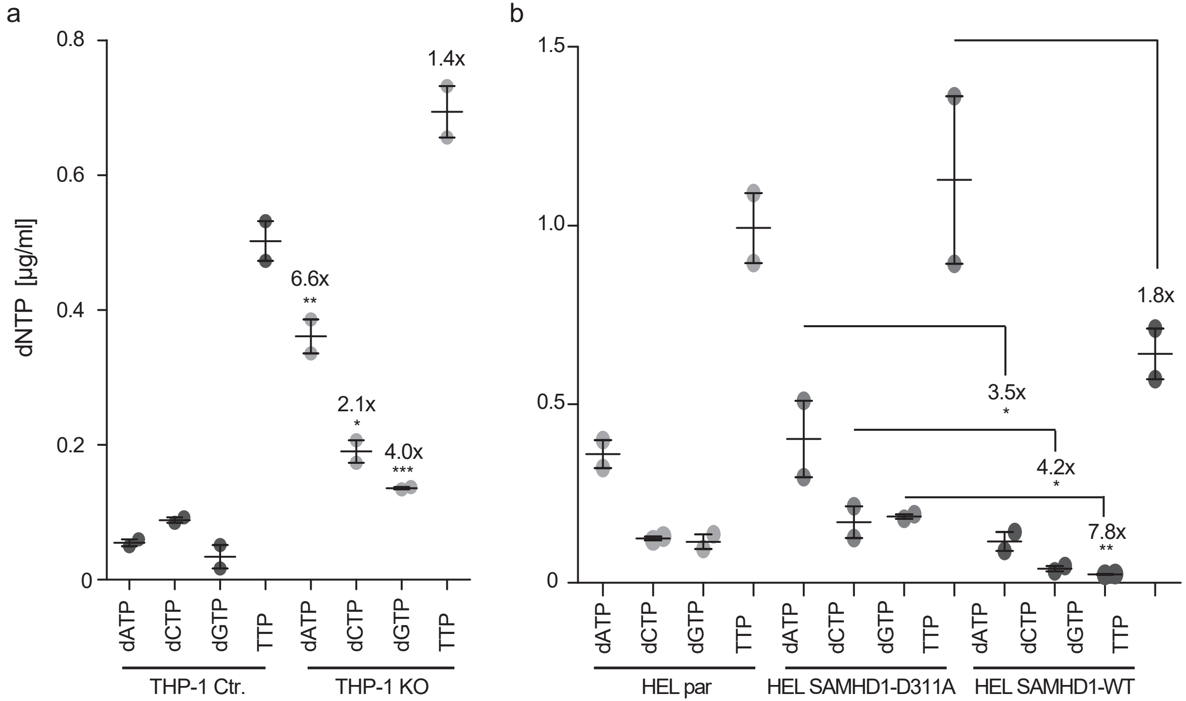

Supplementary Figure 14. Quantification of dNTP levels in SAMHd1 KO and control cells. (a) THP-1 Ctr. and THP-1 SAMHD1 KO cells, and (b) parental HEL cells, HEL cells stabley expressing either SAMHD1-WT or the dNTPase-defective SAMHD1-D311A mutant. Horizontal lines represent means ± s.d of a technical replicate (n=2) out of one representative experiment out of three. Numbers indicate the factor of difference in concentration of the indicated dNTP between cells expressing or not expressing functional SAMHD1. Statistical analyses were performed using unpaired two-tailed Students´t-test. *p ≤ 0.05; **p < 0.01; ***p < 0.001 (Source data are provided as Source Data file).

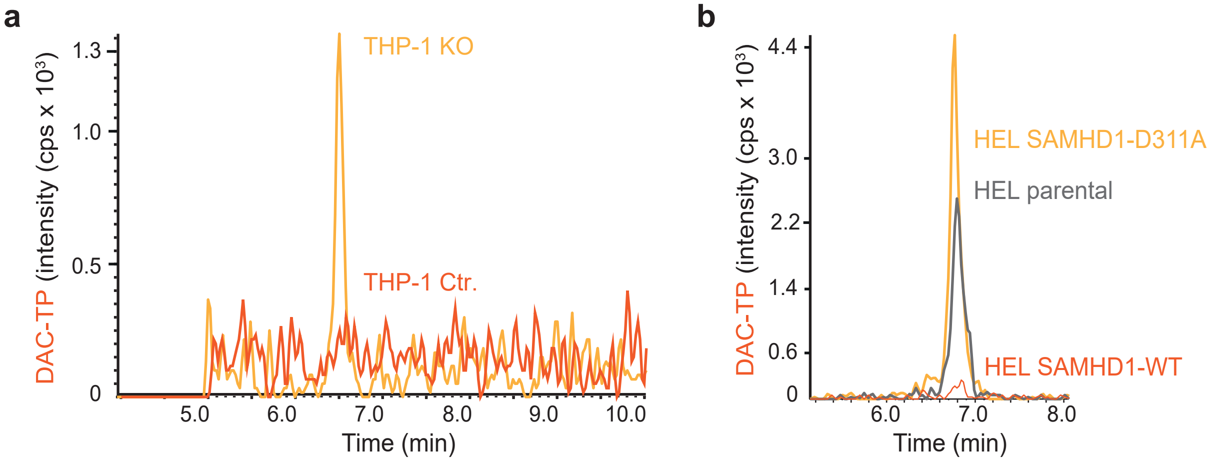

Supplementary Figure 15. DAC-TP levels resulting from metabolic conversion of AZA intermediates depend on SAMHD1. Representative LC-MS/MS chromatograms of DAC-TP in (a) THP-1 control cells (orange) and THP-1 KO cells (yellow) or (b) parental HEL cells (grey) and HEL cells stably expressing either SAMHD1-WT (orange) or the dNTPase-defective SAMHD1-D311A mutant (yellow) (Source data are provided as Source Data file).

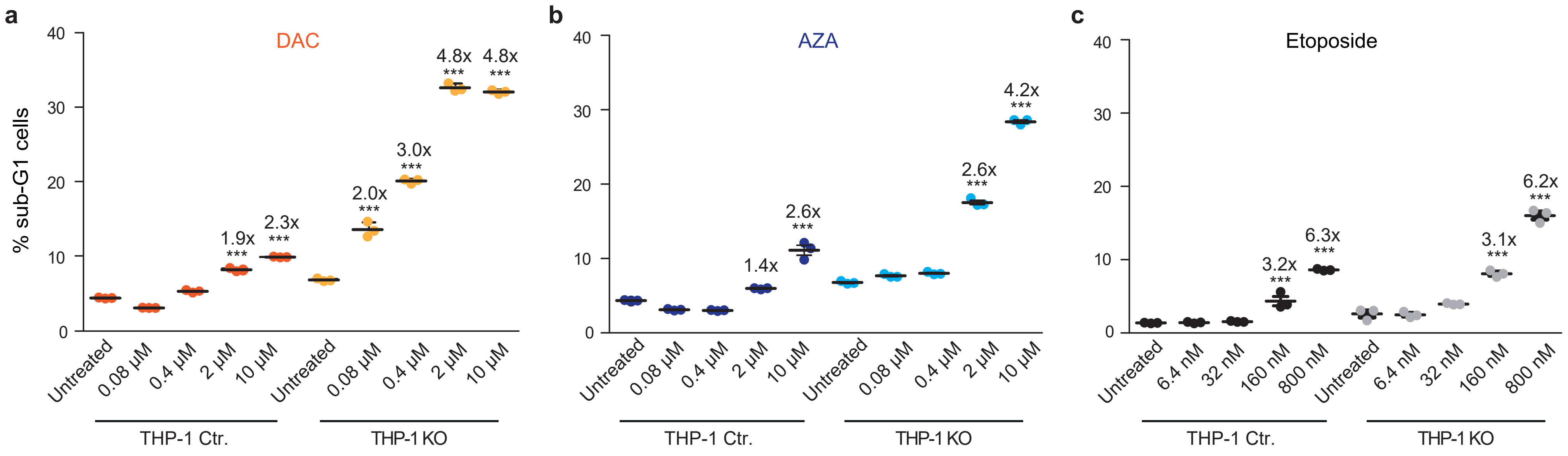

Supplementary Figure 16. DAC treatment induces lower rates of apoptosis in SAMHD1-expressing AML cells. SAMHD1 knockout (THP-1 KO) and control (THP-1 Ctr.) cells were treated with either 0.08, 0.4, 2 or 10 µM of either (a) DAC, (b) AZA, or with (c) 6.4, 32, 160 or 800 nM etoposide for 72 hours or left untreated. Apoptosis rates, quantified by the percentage of sub-diploid DNA peaks (% sub-G1 cells), were measured according to Nicoletti by flow cytometry. Horizontal lines and error bars represent means ± s.d. of a technical replicate (n=3) of one representative experiment out of three. Statistical analyses were performed using unpaired two-tailed Students' t-test comparing treated samples with untreated control. ***$p < 0.001$ (Source data are provided as Source Data file)

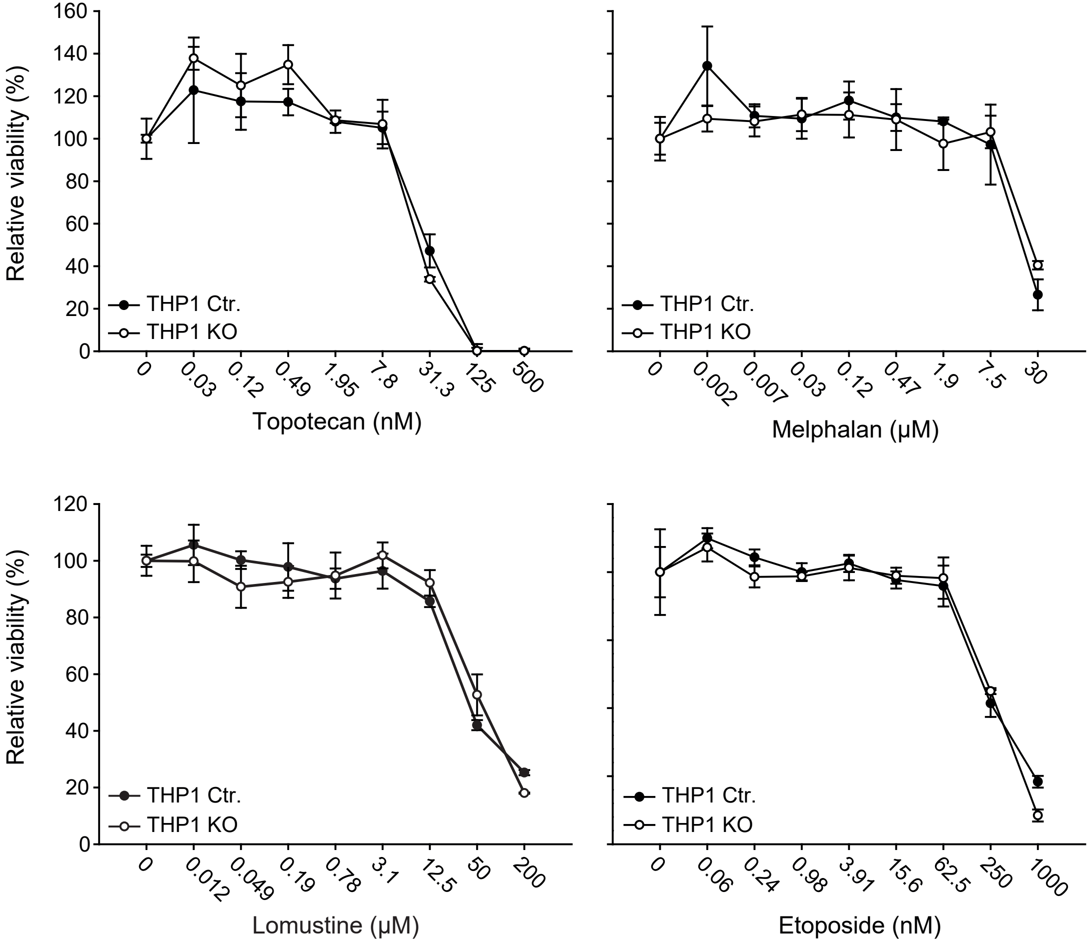

Supplementary Figure 17. SAMHD1 expression has no effect on non-nucleoside cancer drugs. Dose-response analyses of SAMHD1 deficient THP-1 KO and THP-1 Ctr. cells treated with different concentrations of topotecan, lomustine, melphalan or etoposide. After incubation for 96 h viability was quantified by MTT analyses. Values are mean ± s.d. of triplicates of one representative experiment. Three independent experiments were performed (Source data are provided as Source Data file).

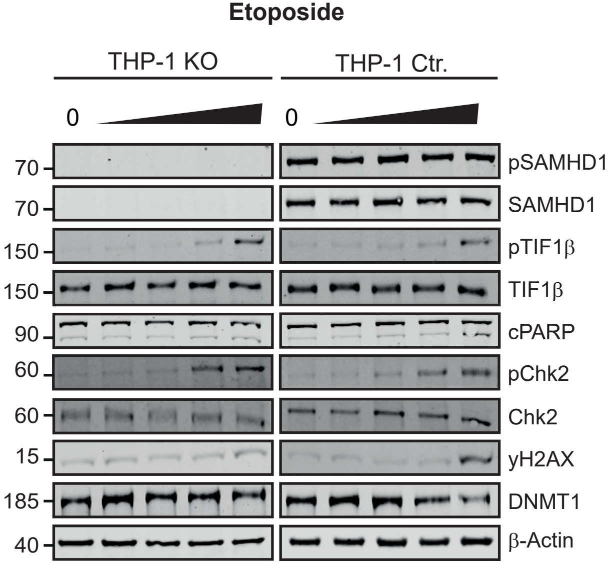

Supplementary Figure 18. SAMHD1 does not influence the cytotoxic effect of etoposide. Representative immunoblots of proteins involved in DNA damage and DNA methylation in THP-1 Ctr. and THP-1 KO cells following treatment with increasing concentrations (0, 1.6, 8, 40 and 200 µM) of etoposide for 48 hours (Source data are provided as Source Data file).

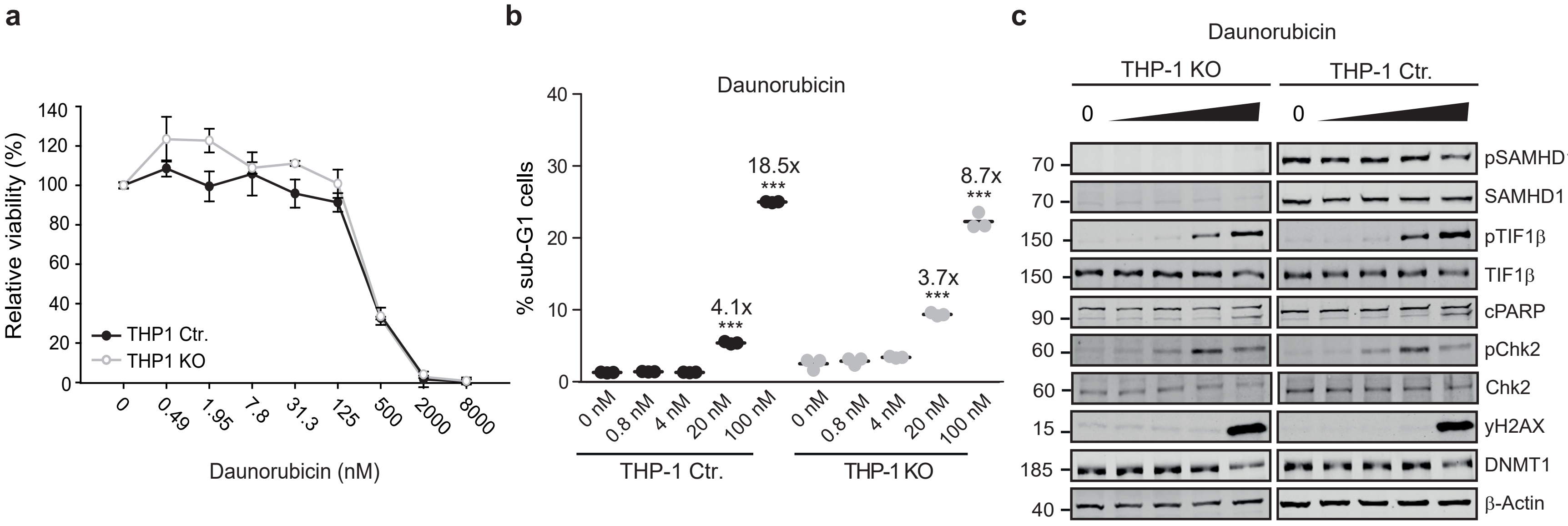

Supplementary Figure 19. Daunorubicin-mediated cytotoxicity is SAMHD1 independent (a) Dose-response analyses in THP-1 Ctr. and THP-1 KO cells treated with different concentrations of daunorubicin and incubated for 96 h before viability was quantified by MTT analyses. Values are mean ± s.d. of triplicates of one representative experiment. Three independent experiments were performed. (b) THP-1 Ctr. and THP-1 KO cells were treated with 100, 20, 4 or 0.8 nM daunorubicin for 72 h or left untreated. Apoptosis rates, quantified by the percentage of sub-diploid DNA peaks (% sub-G1 cells), were measured according to Nicoletti. Horizontal lines and error bars represent means ± s.d. of a technical replicate (n=3) of one representative experiment out of three. Statistical analyses were performed using unpaired two-tailed Students´ t-test, ***$p < 0.001$. (c) Representative immunoblots of proteins involved in DNA damage and DNA methylation in THP-1 Ctr. and THP-1 KO cells following treatment with increasing concentrations (0, 0.8, 4, 20 and 100 µM) of daunorubicin for 72 h (Source data are provided as Source Data file).

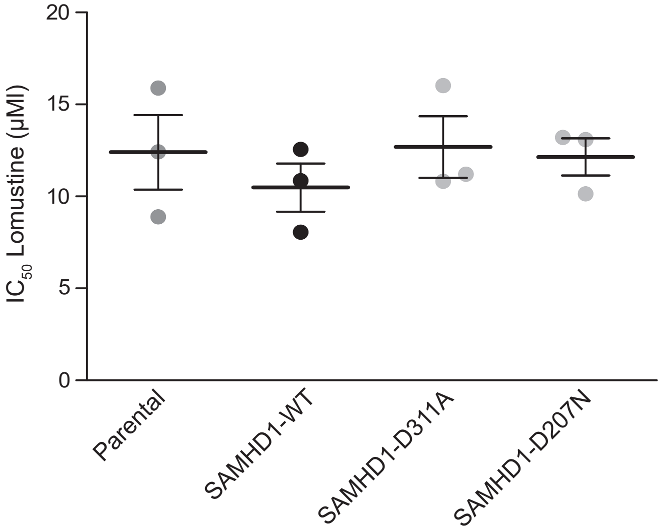

Supplementary Figure 20. SAMHD1 does not affect the cytotoxicity of lomustine. Lomustine IC50 values of parental HEL cells and HEL cells overexpressing either SAMHD1-WT or the dNTPase-defective SAMHD1 mutant SAMHD1-D311A or SAMHD1-D207N. IC50 values of three independent experiments each performed with technical replicates (n=3) are presented as closed circles. Horizontal lines and error bars represent mean ± s.d. (Source data are provided as Source Data file).

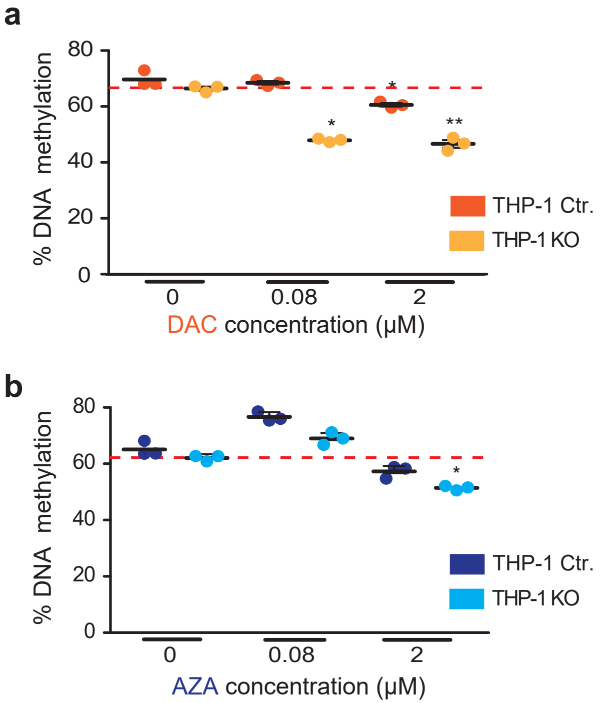

Supplementary Figure 21. Impact of HMAs on DNA methylation in THP-1 cells. Methylation levels of long interspersed elements (LINE)-1 as marker for global DNA methylation was analyzed in THP-1 Ctr. and THP-1 KO cells either left untreated or treated with 0.08 µM or 2µM (a) DAC or (b) AZA for 72 h. Each circle represents a technical replicate (n=3) of one representative experiment out of three. The red dashed line high-lights the DNA methylation level in THP-1 KO cells in the absence of drug. Statistical analyses were performed using unpaired two-tailed Student´s t-test comparing treated with untreated control. $^*p < 0.05$; $^{**}p < 0.01$ (Source data are provided as Source Data file).

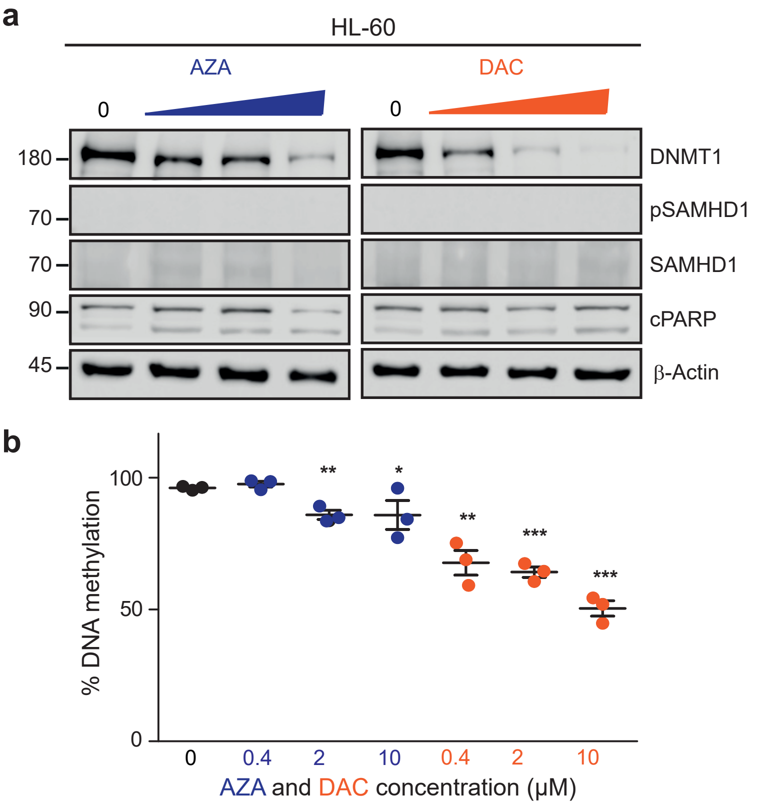

Supplementary Figure 22. Impact of HMAs on DNA damage and methylation in HL-60 cells. (a) Representative immunoblots of SAMHD1 and proteins involved in DNA damage and DNA methylation in HL-60 cells following treatment with increasing concentrations (0, 0.4, 2 and 10 μM) of either AZA or DAC for 48 hours. (b) Methylation levels of LINE-1 elements were analyzed in cells either left untreated or after treatment with 0.4, 2 or 10 μM of either AZA or DAC for 48 hours. Each circle represents a technical replicate (n=3) of one representative experiment out of three. Statistical analyses were performed using unpaired two-tailed Students' t-test comparing treated samples with untreated control. *$p < 0.05$; **$p < 0.01$; ***$p < 0.001$ (Source data are provided as Source Data file).

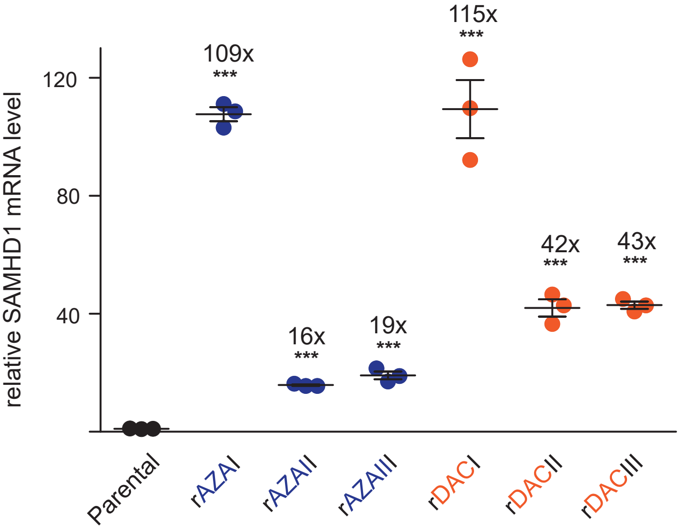

Supplementary Figure 23. Resistance to AZA and DAC increases SAMHD1 mRNA expression. qPCR-based analyses of SAMHD1 mRNA expression in parental, AZA- and DAC-resistant HL-60 cells. Bars and error bars represent mean ± s.d. of three independent experiment each was performed with technical replicates (n=3). Numbers indicate the factor of difference between the resistant cell lines and the parental control. Statistical analyses were performed using unpaired two-tailed Students' t-test comparing treated samples with untreated control. ***p < 0.001 (Source data are provided as Source Data file).

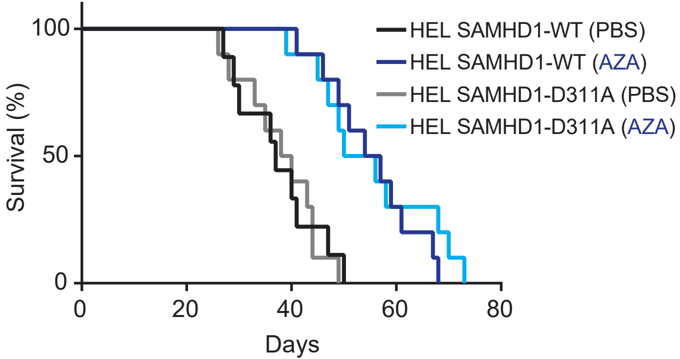

Supplementary Figure 24. Kaplan-Meier survival analyses of xenotransplanted mice treated with AZA. NGS mice transplanted with HEL cells expressing either SAMHD1-wildtype (WT) or the catalytically inactive D311A mutant were treated with AZA. AZA was administered i.v. (3.2 mg per kg 5x per week); PBS (control treatment). All differences were non-significant (Source data are provided as Source Data file).

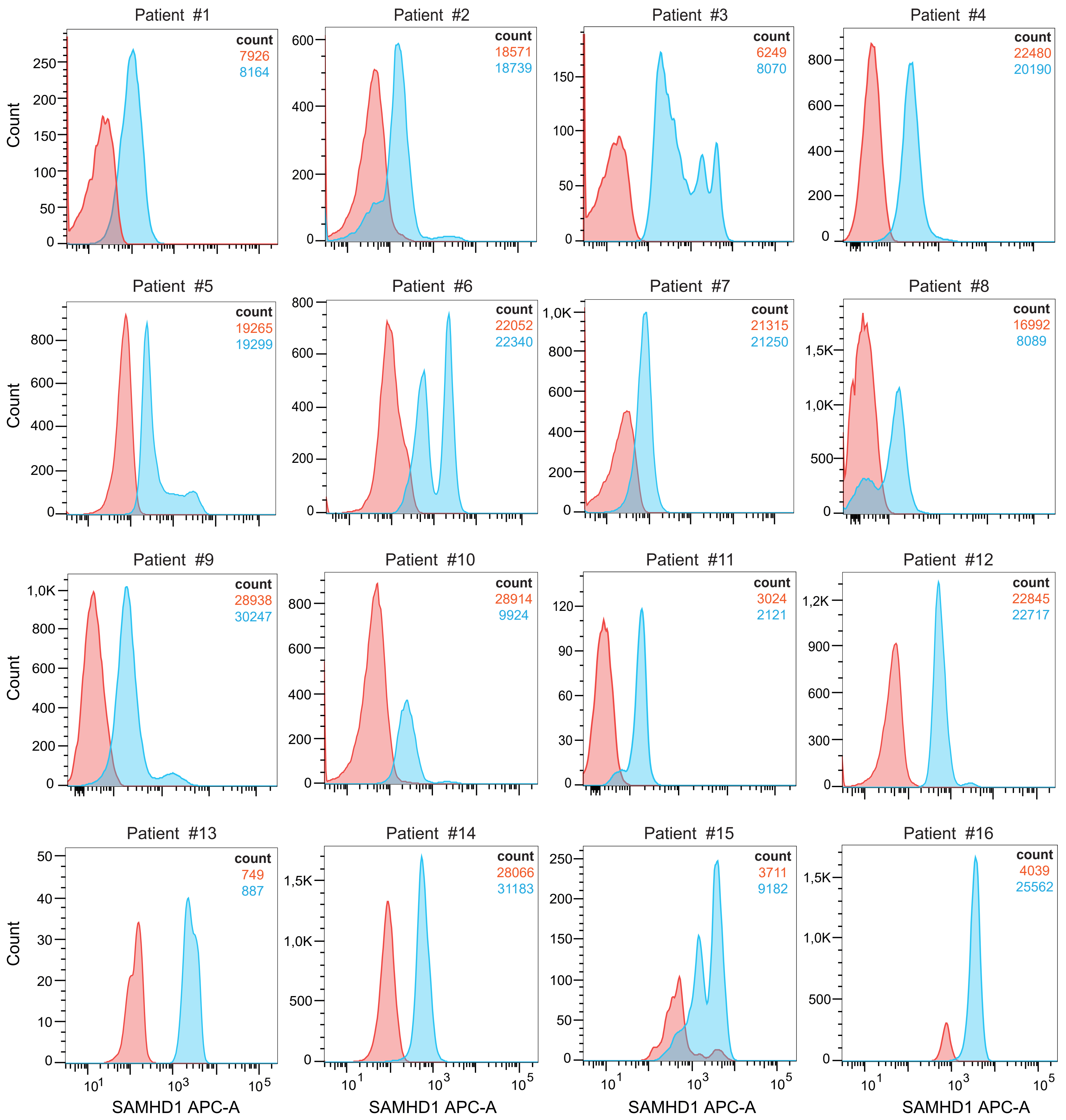

Supplementary Figure 25a. FACS analyses of AML blasts. AML patient samples (see also Supplementary Table 3) with a purity >80% were coimmunostained for CD45, CD33, CD34 (surface markers), and intracellular SAMHD1 and analyzed by flow cytometry (see also Supplementary Figure 27 for gating strategies). Histograms show SAMHD1 expression in CD33+, CD34+,CD45+ AML blasts (blue). The isotype control staining is depicted in red. Numbers indicate the cell count for each population (Source data are provided as Source Data file).

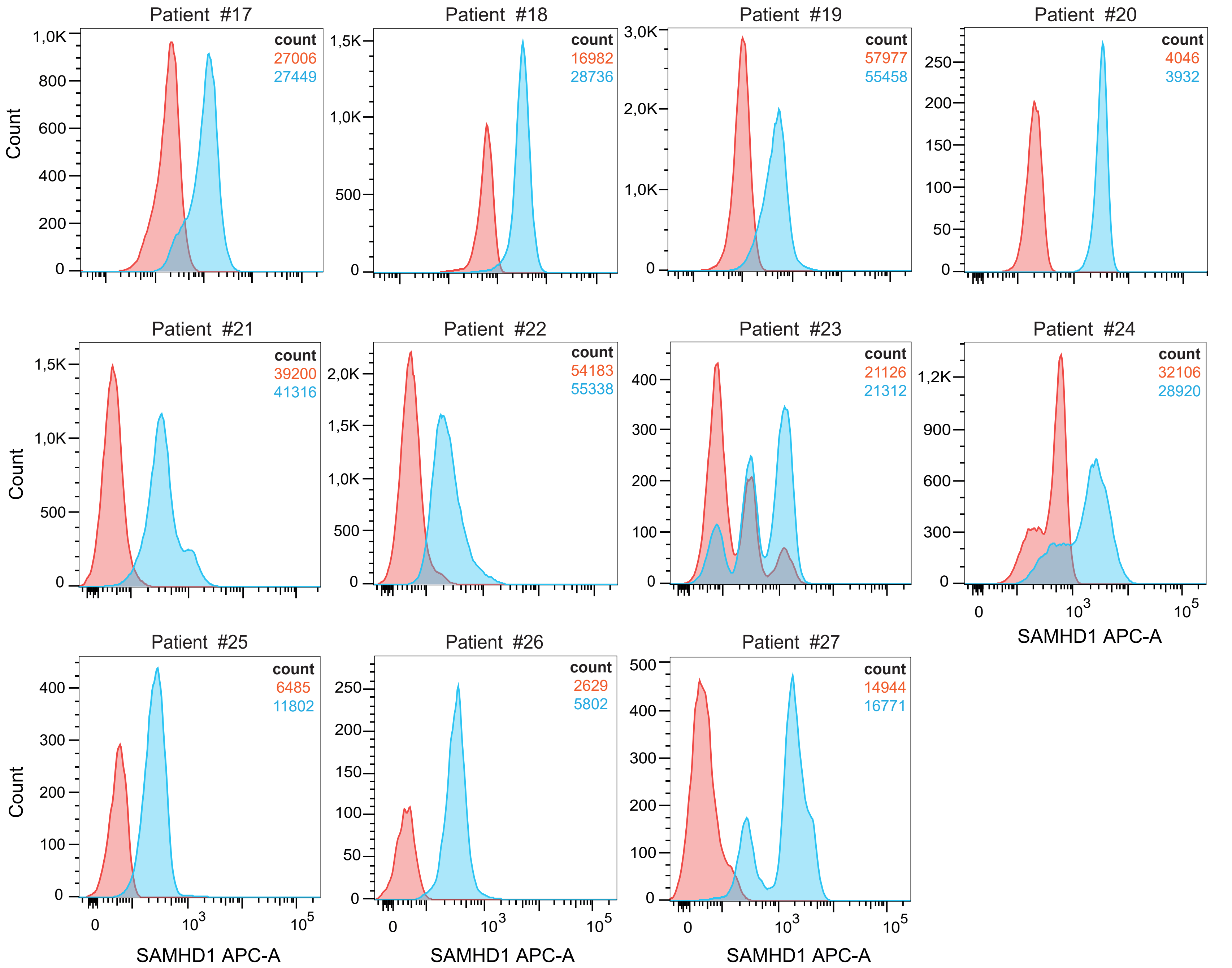

Supplementary Figure 25b. FACs analyses of AML blasts. AML patient samples (see also Supplementary Table 3) with a purity >80 % were co-immunostained for CD45, CD33, CD34 (surface markers), and intracellular SAMHD1 and analyzed by flow cytometry (see also Supplementary Figure 27 for gating strategies). Histograms sho SAMHD1 Expression in CD33+, CD34+, CD45+ AML blasts (blue). The isotype control staining is depicted in red. Numbers indicate the cell count for each population (Source data are provided as Source Data file).

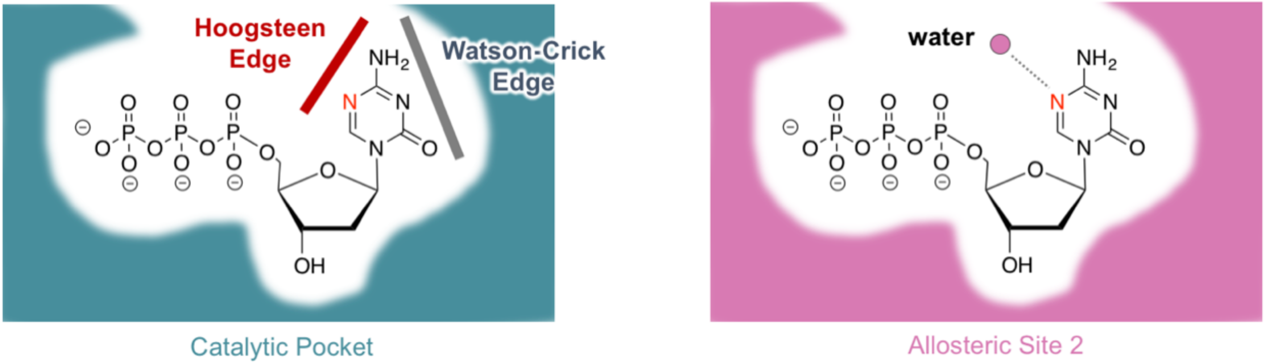

Supplementary Figure 26. The effect of Hoogsteen edge modifications on nucleotide binding to SAMHD1´s catalytic pocket and allosteric site 2. Modest modification to the Hoogsteen edge of a nucleotide is not likely to interfere with binding to the catalytic pocket or allosteric site 2. Both pockets have room to accommodate modest modifications to the Hoogsteen edge and this region does not normally participate in direct binding to the protein. Nucleotide interactions with SAMHD1 are mostly mediated through the Watson-Crick edge of the base and the sugar and phosphate groups. In the case of DAC-TP in allosteric site 2, an additional water happens to hydrogen bond to the modification.

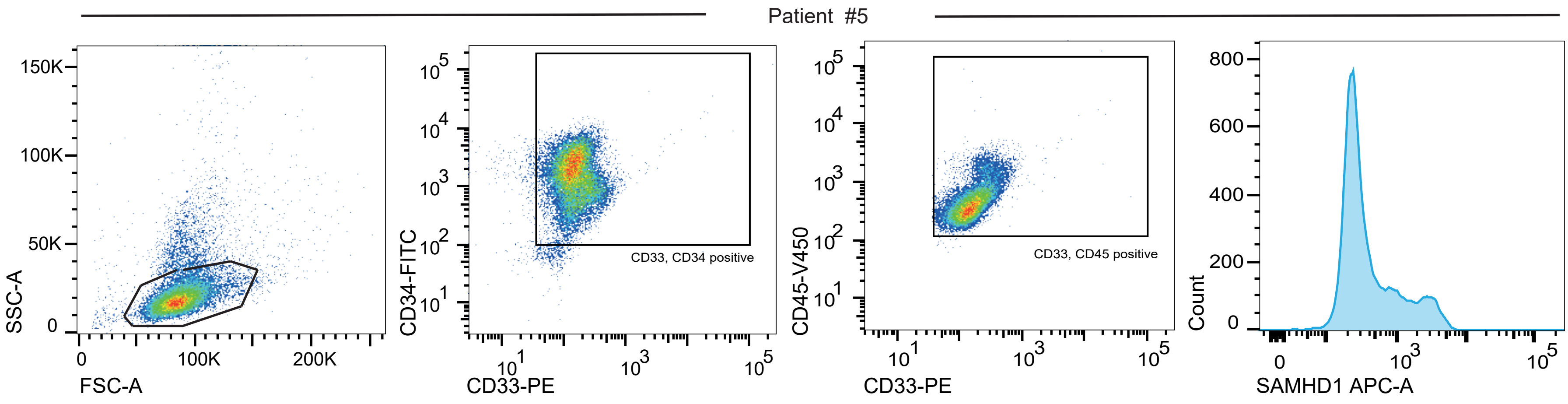

Supplementary Figure 27. Gating strategy for freshly isolated bone marrow samples from AML patients. Panels show data for patient #5 (see also Supplementary Table 3 for details). AML patient samples with a purity > 80% were co-immunostained for CD45, CD33, CD34 (surface markers), and intracellular SAMHD1 and analyzed by flow cytometry. Gates depict CD33+, CD34+ leukemic cells (second panel from the left), which were further analyzed for CD45 expression (second panel from the right). Histograms show the SAMHD1 expression in CD33+, CD34+,CD45+ cells of AML patient #5 (right panel). Data were used in Figure 6a-c for correlative analysis (Source data are provided as Source Data file).

Supplementary Table 1 I Data collection and refinement statistics for DAC-TP co-crystal structure with hSAMHD1 HD. Statistics in parentheses indicate those for the highest resolution shell.

Data Collection	
**Wavelength (Å)**	0,98
**Space Group**	P 21
**Cell dimensions**	
**a, b, c (Å)**	87.5, 146.5, 98.6
**α, β, γ (°)**	90.0, 114.6, 90.0
**Molecules/asymetric unit**	4
**Resolution (Å)**	50-2.14 (2.18-2.14)
**Unique reflections**	125603 (6000)
**Rmerge**	0.132 (>1)
**I/sigma**	9.3 (0.9)
**Completeness (%)**	98.9 (95.0)
**Redundancy**	4.1 (3.3)
**CC½**	0.993 (0.175)
**Refinement**	
**No. reflections**	125515
**Rwork/ Rfree (%)**	17.4/22.5 (33.7/34.7)
**No. atoms**	16496
**Protein**	15772
**Ligand/ion**	356
**B-factors**	
**Protein**	42
**Ligand/ion**	33
**Water**	37
**RMSD**	
**Bond lengths (Å)**	0,02
**Bond angles (°)**	1,86
**Ramachandran Analysis**	
**Preferred regions (%)**	97,1
**Allowed regions (%)**	2,8
**Outliers (%)**	0,1
**PDB ID**	6CM2

Supplementary Table 2 I Concentration of AZA, DAC and SGI-110 that reduce the viability of leukemic cell lines by 50% (IC50) and LC-MS/MS analysis of AZA-TP and DAC-TP. Source data are available as Source Data file.

**Cell line**	**IC50 values (µM)**			**TP intensity (cps, x 10^4)**	
	AZA	DAC	SGI-110	AZA	DAC
THP-1	10.6 ± 1.4	8.5 ± 1.1	7.8 ± 1.8	5.5 ± 3.3	0.08 ± 0.03
OCI-AML3	6.1 ± 1.5	0.94 ± 0.20	0.29 ± 0.02	22.7 ± 3.0	0.68 ± 0.19
OCI-AML2	2.5 ± 0.6	0.48 ± 0.05	0.27 ± 0.04	2.7 ± 0.3	0.19 ± 0.03
Molm-13	1.3 ± 0.3	0.13 ± 0.03	0.12 ± 0.02	11.9 ± 1.8	0.87 ± 0.39
MV4-11	3.6 ± 0.5	2.4 ± 0.5	0.77 ± 0.11	2.2 ± 0.6	0.11 ± 0.03
PL21	4.5 ± 0.5	0.13 ± 0.06	0.29 ± 0.04	39.1 ± 10.3	5.9 ± 2.2
HL-60	12.4 ± 2.8	0.22 ± 0.03	0.41 ± 0.03	8.6 ± 4.1	9.6 ± 2.4
MonoMac6	0.92 ± 0.10	0.93 ± 0.45	1.3 ± 0.5	13.5 ± 2.2	0.57 ± 0.07
NB4	0.56 ± 0.17	0.09 ± 0.004	0.02 ± 0.004	5.4 ± 3.1	1.2 ± 0.5
KG1	9.9 ± 3.9	0.11 ± 0.04	0.10 ± 0.03	13.2 ± 6.3	3.5 ± 0.8
SIG-M5	1.4 ± 0.7	0.20 ± 0.09	1.1 ± 0.08	3.2 ± 0.6	0.63 ± 0.14
HEL	8.6 ± 0.9	0.33 ± 0.11	0.29 ± 0.03	37.9 ± 10.2	4.1 ± 0.7
ML2	2.3 ± 0.2	1.6 ± 0.1	3.5 ± 0.3	4.8 ± 2.3	0.28 ± 0.16
