# Supplementary material for: Selective inactivation of hypomethylating agents by SAMHD1 provides a rationale for therapeutic stratification in AML
Source: Nat Commun. 2019 Aug 2;10:3475. doi: 10.1038/s41467-019-11413-4 (PMC6677770; doi:10.1038/s41467-019-11413-4)
Supplement: Supplementary file 2 — Description of Additional Supplementary Files [file 41467_2019_11413_MOESM2_ESM.pdf]

Description of Additional Supplementary Files

File Name: Supplementary Data 1
Description: Characteristics of AML patients analysed in the data presented in Figure 6a-g.

File Name: Supplementary Data 2
Description: Characteristics of AML patients from the DAC-treated cohort analysed in the data presented in Figs. 6F-H.
