# Supplementary material for: Selective inactivation of hypomethylating agents by SAMHD1 provides a rationale for therapeutic stratification in AML
Source: Nat Commun. 2019 Aug 2;10:3475. doi: 10.1038/s41467-019-11413-4 (PMC6677770; doi:10.1038/s41467-019-11413-4)
Supplement: Supplementary file 5 — Reporting Summary [file 41467_2019_11413_MOESM5_ESM.pdf]

nature research

Corresponding author(s): Jindrich Cinatl Jr (Cinatl@em.uni-frankfurt.de)

Last updated by author(s): Jun 4, 2019

Reporting Summary

Nature Research wishes to improve the reproducibility of the work that we publish. This form provides structure for consistency and transparency in reporting. For further information on Nature Research policies, see Authors & Referees and the Editorial Policy Checklist.

Statistics

For all statistical analyses, confirm that the following items are present in the figure legend, table legend, main text, or Methods section.

n/a	Confirmed	
☐	☒	The exact sample size (*n*) for each experimental group/condition, given as a discrete number and unit of measurement
☐	☒	A statement on whether measurements were taken from distinct samples or whether the same sample was measured repeatedly
☐	☒	The statistical test(s) used AND whether they are one- or two-sided<br>*Only common tests should be described solely by name; describe more complex techniques in the Methods section.*
☒	☐	A description of all covariates tested
☒	☐	A description of any assumptions or corrections, such as tests of normality and adjustment for multiple comparisons
☐	☒	A full description of the statistical parameters including central tendency (e.g. means) or other basic estimates (e.g. regression coefficient) AND variation (e.g. standard deviation) or associated estimates of uncertainty (e.g. confidence intervals)
☐	☒	For null hypothesis testing, the test statistic (e.g. *F*, *t*, *r*) with confidence intervals, effect sizes, degrees of freedom and *P* value noted<br>*Give P values as exact values whenever suitable.*
☒	☐	For Bayesian analysis, information on the choice of priors and Markov chain Monte Carlo settings
☒	☐	For hierarchical and complex designs, identification of the appropriate level for tests and full reporting of outcomes
☒	☐	Estimates of effect sizes (e.g. Cohen's *d*, Pearson's *r*), indicating how they were calculated

Our web collection on statistics for biologists contains articles on many of the points above.

Software and code

Policy information about availability of computer code

Data collection	no software was used
Data analysis	Statistical data analysis was performed in R, version 3.3.2

For manuscripts utilizing custom algorithms or software that are central to the research but not yet described in published literature, software must be made available to editors/reviewers. We strongly encourage code deposition in a community repository (e.g. GitHub). See the Nature Research guidelines for submitting code & software for further information.

Data

Policy information about availability of data

All manuscripts must include a data availability statement. This statement should provide the following information, where applicable:

- Accession codes, unique identifiers, or web links for publicly available datasets
- A list of figures that have associated raw data
- A description of any restrictions on data availability

The atomic coordinates and structure factors have been deposited in the Protein Data Bank, www.pdb.org (PDB ID code 6CM2 [https://www.rcsb.org/structure/4TNP]). The source data underlying Figs 1-7 and Supplementary Figs 1-27 are provided as a Source Data file
Source data are available at figshare [...]

Field-specific reporting

Please select the one below that is the best fit for your research. If you are not sure, read the appropriate sections before making your selection.

☒ Life sciences ☐ Behavioural & social sciences ☐ Ecological, evolutionary & environmental sciences

For a reference copy of the document with all sections, see nature.com/documents/nr-reporting-summary-flat.pdf

Life sciences study design

All studies must disclose on these points even when the disclosure is negative.

Sample size	No sample-size calculation was performed. Cell culture experiments were done in three independent triplicates, patient sample-size was limited by their availability.
Data exclusions	Patient samples were excluded if the IC50 values in viability assays could not been determined because of decreased cell growth after isolation of the AML blasts. No other data were excluded.
Replication	All replicates were successful, showing the reproducibility of the experimental findings.
Randomization	n.a.
Blinding	Two pathologists, who were blinded to clinical history and therapeutic response, independently scored the SAMHD1 immunostaining of bone marrow samples.

Reporting for specific materials, systems and methods

We require information from authors about some types of materials, experimental systems and methods used in many studies. Here, indicate whether each material, system or method listed is relevant to your study. If you are not sure if a list item applies to your research, read the appropriate section before selecting a response.

Materials & experimental systems

n/a	Involved in the study	
☐	☒	Antibodies
☐	☒	Eukaryotic cell lines
☒	☐	Palaeontology
☐	☒	Animals and other organisms
☐	☒	Human research participants
☐	☒	Clinical data

Methods

n/a	Involved in the study	
☒	☐	ChIP-seq
☐	☒	Flow cytometry
☒	☐	MRI-based neuroimaging

Antibodies

Antibodies used	SAMHD1 (12586-1-AP, Proteintech), β-actin (3598R-100, BioVision via BioCat), DCK (sc-393099, Santa Cruz), CDA (sc-365292, Santa Cruz), ENT1 (ab135756, Abcam), UCK1 (Thermo Scientific), UCK2 (PA5-14010, Thermo Scientific), RRM1 (sc-377415, Santa Cruz), RRM2 (sc-398294, Santa Cruz), OCTN1 (H00006583-A01, Abnova), DCTD (NBP1-75825, Novus), cPARP (9542S, Cell Signaling), γH2AX (9718S, Cell Signaling), Chk2 (2662S, Cell Signaling), pChk2 (2661S, Cell Signaling), TIF1β (4123S, Cell Signaling), pTIF1β (4127S, Cell Signaling), DNMT1 (39204, Active Motif). For Flow cytometry CD33-PE (130-091-732), CD34-FITC (130-081-001, both from Miltenyi Biotech), and CD45-V450 (560373, BD Pharmingen) were used.
Validation	Primary antibodies were sufficiently validated by the companies or in literature. For more details see the repective manufacturer ´s websites.

Eukaryotic cell lines

Policy information about cell lines

Cell line source(s)	THP-1 (DSMZ no. ACC11; FAB M6), OCI-AML2 (DSMZ No. ACC 99; FAB M4), OCI-AML3 (DSMZ No. ACC 582; FAB M4), Molm13 (DSMZ No. ACC 554; FAB M5a), PL-21 (DSMZ No. ACC 536; FAB M3), HL-60 (DSMZ No. ACC 3; FAB M2), MV4-11 (DSMZ No. ACC 102; FAB M5), SIG-M5 (DSMZ No. ACC 468; FAB M5a), ML2 (DSMZ No. ACC 15; FAB M4), NB4 (DSMZ No. ACC 207; FAB M3), KG1 (DSMZ No. ACC 14; FAB not indicated), MonoMac6 (DSMZ No. ACC 124; FAB M5), and HEL (DSMZ No. ACC 11; FAB M6) were obtained from DSMZ (Deutsche Sammlung von Mikroorganismen und Zellkulturen GmbH).
Authentication	Cells were authenticated by short tandem repeat profiling (PowerPlex 16 System, Promega)

Mycoplasma contamination	Cells were routinely tested for mycoplasma contamination (LT07-710, Lonza). All cell lines were tested negative.
Commonly misidentified lines (See ICLAC register)	n.a.

Animals and other organisms

Policy information about studies involving animals; ARRIVE guidelines recommended for reporting animal research

Laboratory animals	Female non-obese diabetic severe combined immunodeficient gamma (NSG) mice were purchased from Jackson ImmunoResearch laboratories (Ban Harbor, ME). All mice used in the experiments were between 6 and 10 weeks of age. All animal experiments were performed according to the regulations of the United Kingdom Home Office and German authorities.
Wild animals	n.a.
Field-collected samples	n.a.
Ethics oversight	All animal experiments were performed according to the regulations of the United Kingdom Home Office and German authorities.

Note that full information on the approval of the study protocol must also be provided in the manuscript.

Human research participants

Policy information about studies involving human research participants

Population characteristics	Bone marrow samples were obtained from 27 patients with an initial diagnosis of acute myeloid leukemia. Median age was 58 years (range 35-86) with a predominance of female sex (55.6%). Myeloid blasts (mean blast count: 66%; range 25-83%) were routinely screened for following mutations in: NPM1, FLT3, RUNX1/RUNXT1, PML-RARA, CEBPA, BCR-ABL1, CBFb-MYH11, KMT2A-ELL, -MLLT3,-T4, KMT2A-PTD; IDH1/2. In 21/27 patients (77.8%) cytogenetic analysis was performed and 76.2% revealed a normal karyotype.
Recruitment	Patients were admitted to the Frankfurt and Münster University Hospitals between 2012 and 2017 and were treated for newly diagnosed AML with regimens containing standard dose decitabine or azacytidine. In addition, viable AML cells were purified from the bone marrow of patients who were admitted to the University Hospital Frankfurt in 2016 and 2017. Patients at the Frankfurt and Münster University Hospitals are routinely advised to undergo a bone-marrow biopsy at diagnosis. All patients consented to the scientific analyses of their data and of biomaterial obtained for diagnostic purposes. Whole blood and bone marrow biopsies from patients with AML were obtained and collected pre- and post-treatment. All patients gave informed consent according to the Declaration of Helsinki to participate in the collection of samples.
Ethics oversight	The use of whole blood and bone marrow aspirates was approved by the Ethics Committee of Frankfurt University Hospital (approval no. SHN-11-2016 and SHN-03-2017) and University Hospital Muenster (approval no. 2007-390-f-S)

Note that full information on the approval of the study protocol must also be provided in the manuscript.

Clinical data

Policy information about clinical studies

All manuscripts should comply with the ICMJE guidelines for publication of clinical research and a completed CONSORT checklist must be included with all submissions.

Clinical trial registration	n.a.
Study protocol	n.a.
Data collection	Patients were admitted to the Frankfurt and Münster University Hospitals between 2012 and 2017 and were treated for newly diagnosed AML with regimens containing standard dose decitabine or azacytidine. In addition, viable AML cells were purified from the bone marrow of patients who were admitted to the University Hospital Frankfurt in 2016 and 2017. Patients at the Frankfurt and Münster University Hospitals are routinely advised to undergo a bone-marrow biopsy at diagnosis. All patients consented to the scientific analyses of their data and of biomaterial obtained for diagnostic purposes. All patients gave informed consent according to the Declaration of Helsinki to participate in the collection of samples. The use of whole blood and bone marrow aspirates was approved by the Ethics Committee of Frankfurt University Hospital (approval no. SHN-11-2016 and SHN-03-2017) and University Hospital Muenster (approval no. 2007-390-f-S). For the analyses, patient records were reviewed by physicians who were unaware of the SAMHD1 expression results in the diagnostic biopsies. Remission criteria and cytogenetic risk groups were assessed according to the ELN guidelines.
Outcomes	The best response to DAC or AZA therapy was analyzed in bone-marrow biopsies and aspirates and defined as complete (CR) if the blast count was < 5%, and as “no CR” if the blast count was > 5%.

Flow Cytometry

Plots

Confirm that:

☒ The axis labels state the marker and fluorochrome used (e.g. CD4-FITC).

☒ The axis scales are clearly visible. Include numbers along axes only for bottom left plot of group (a 'group' is an analysis of identical markers).

☒ All plots are contour plots with outliers or pseudocolor plots.

☒ A numerical value for number of cells or percentage (with statistics) is provided.

Methodology

Sample preparation	Staining for surface markers (CD33, CD34, CD45) was applied prior to fixation. The following fluorochrome-conjugated antibodies were used: CD33-PE, CD34-FITC and CD45-V450, all diluted 1:11 per 10^7 cells, and Alexa-Fluor-660 (A-21074, Invitrogen, Life technologies, 1:200).
Instrument	Samples were analyzed using a FACSVerse flow cytometer (BD Biosciences)
Software	FlowJo software (TreeStar)
Cell population abundance	Mononuclear cells from blood or bone-marrow AML samples were purified by Ficoll-Hypaque gradient centrifugation. Leukemic cells were enriched by negative selection with a combination of CD3-, CD19- and CD235a-microbeads (all obtained from Miltenyi Biotec) according to the manufacturer's instructions and separated by the autoMACS™ Pro Separator. All preparations were evaluated for purity, resulting in >80% leukemic blasts.
Gating strategy	AML patient samples with a purity > 80% were co-immunostained for CD45, CD33, CD34 (surface markers), and intracellular SAMHD1 and analyzed by flow cytometry. Gates depict CD33+, CD34+ leukemic cells, which were further analyzed for CD45 expression. Histograms show the SAMHD1 expression in CD33+, CD34+, CD45+ cells of the AML patient (see Supplementary figure 27 for more details).

☒ Tick this box to confirm that a figure exemplifying the gating strategy is provided in the Supplementary Information.
