# Supplementary material for: Selective inactivation of hypomethylating agents by SAMHD1 provides a rationale for therapeutic stratification in AML
Source: Nat Commun. 2019 Aug 2;10:3475. doi: 10.1038/s41467-019-11413-4 (PMC6677770; doi:10.1038/s41467-019-11413-4)

Figure 2a

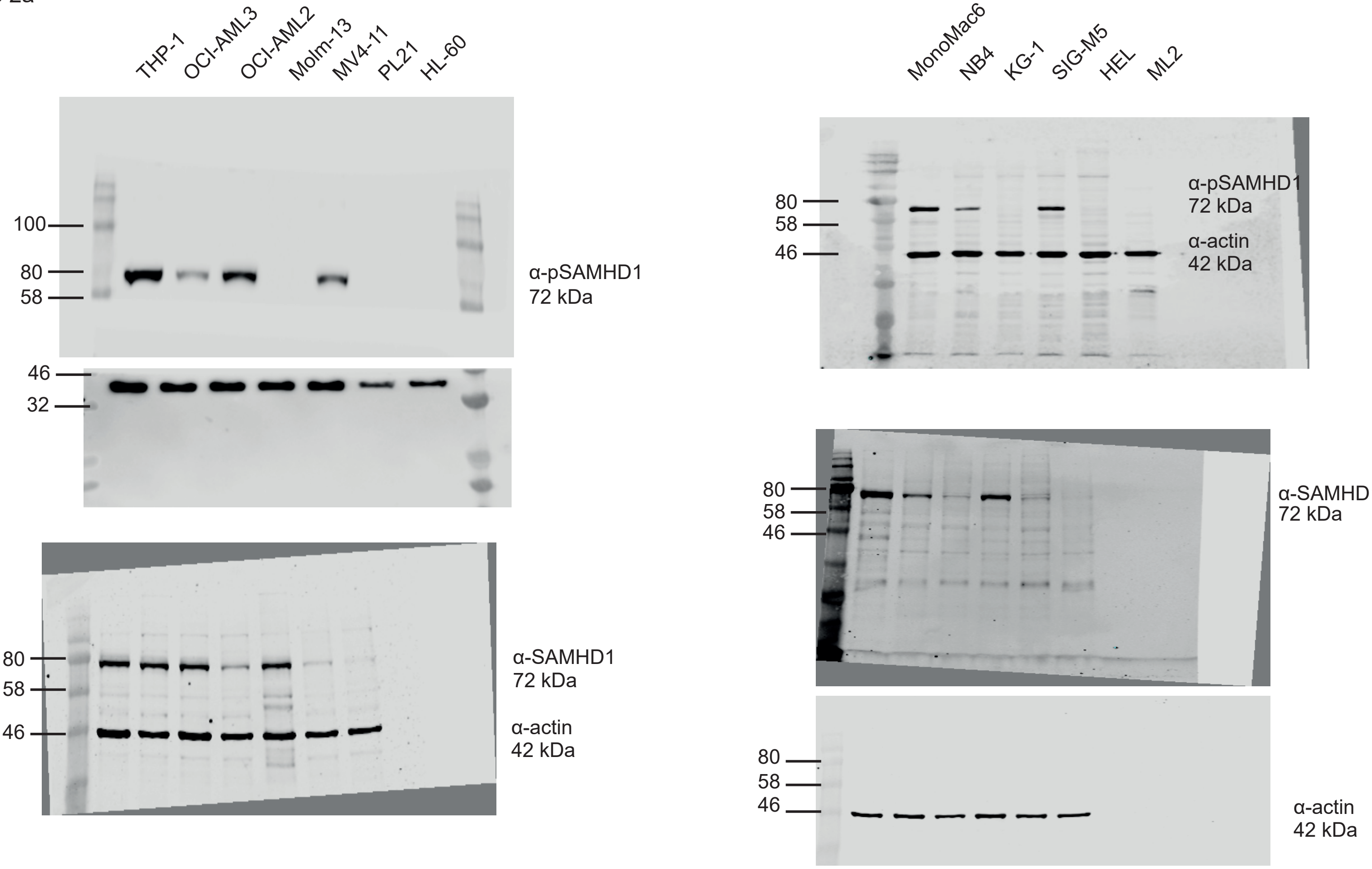

Figure 3b

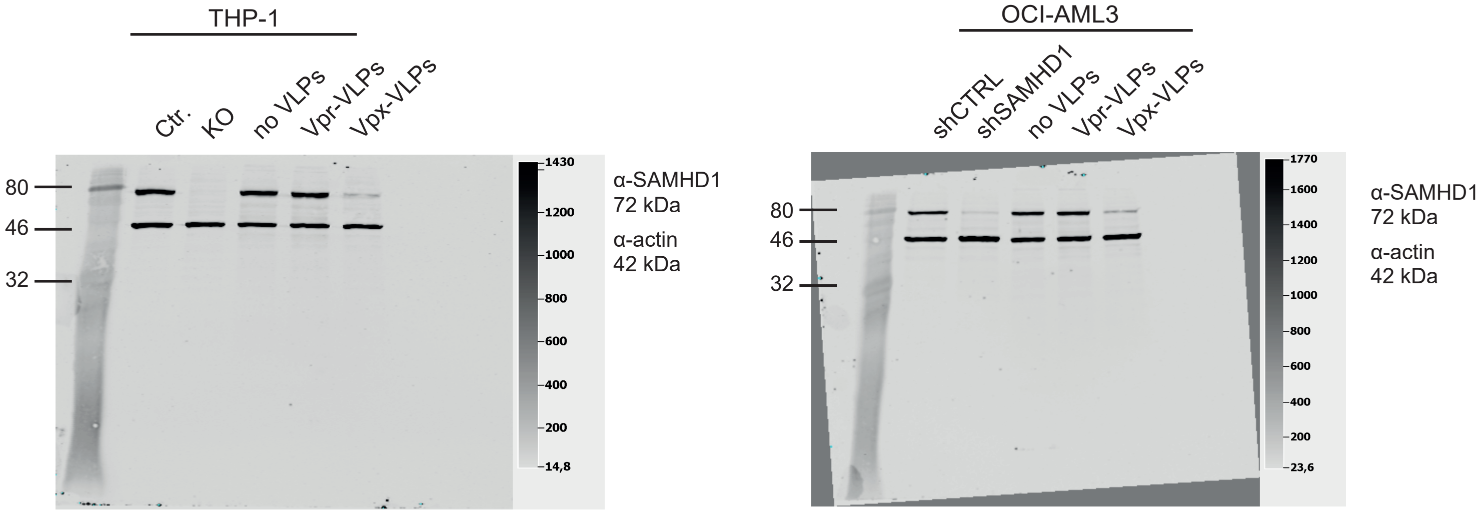

Figure 3e

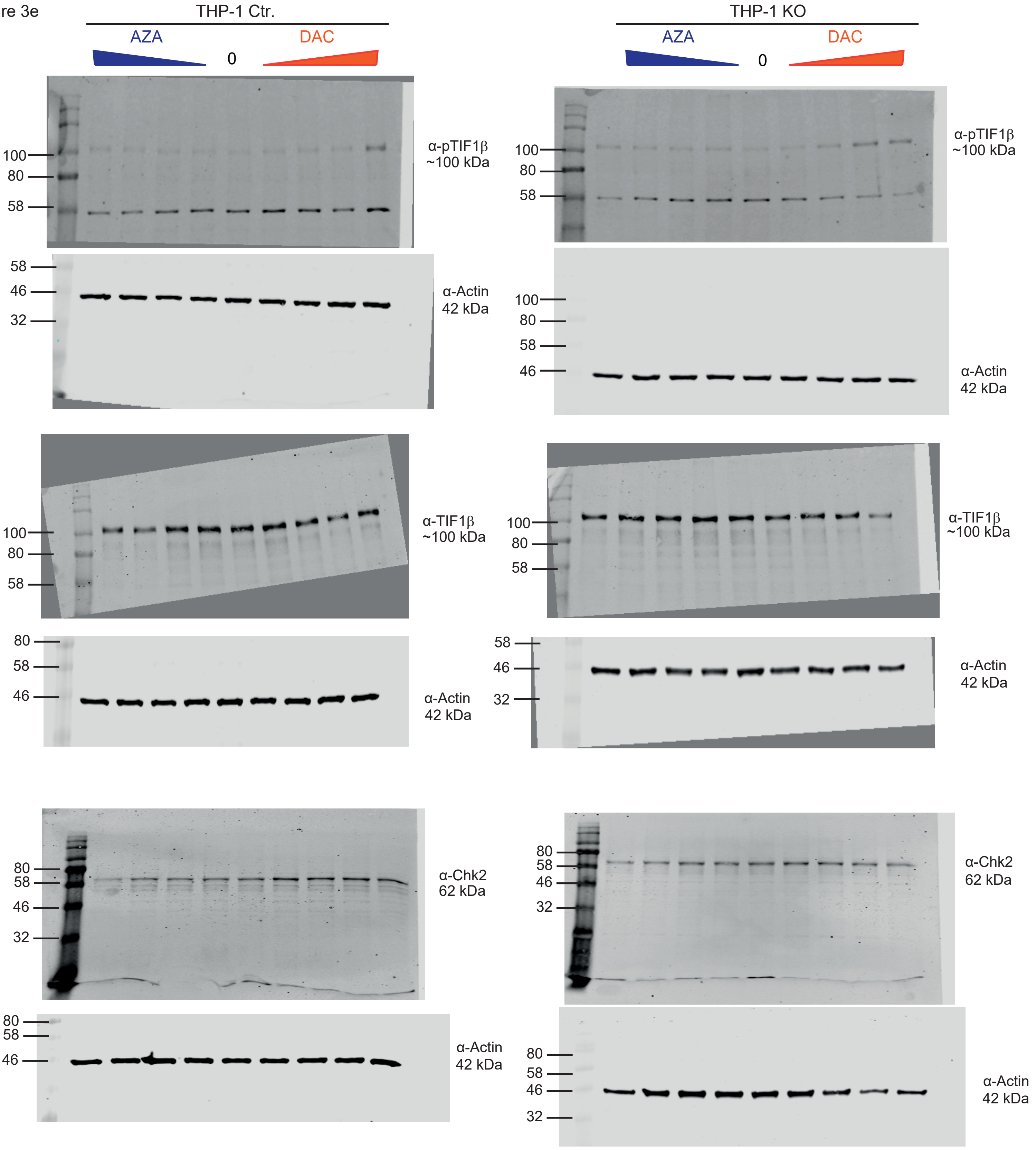
THP-1 Ctr.
AZA
0
DAC
THP-1 KO
AZA
0
DAC
100
80
58
α-pTIF1β
~100 kDa
58
46
32
α-Actin
42 kDa
100
80
58
α-pTIF1β
~100 kDa
100
80
58
46
α-Actin
42 kDa
100
80
58
α-TIF1β
~100 kDa
80
58
46
α-Actin
42 kDa
100
80
58
α-TIF1β
~100 kDa
58
46
32
α-Actin
42 kDa
80
58
46
32
α-Chk2
62 kDa
80
58
46
α-Actin
42 kDa
80
58
46
32
α-Chk2
62 kDa
80
58
46
32
α-Actin
42 kDa

Figure 3e

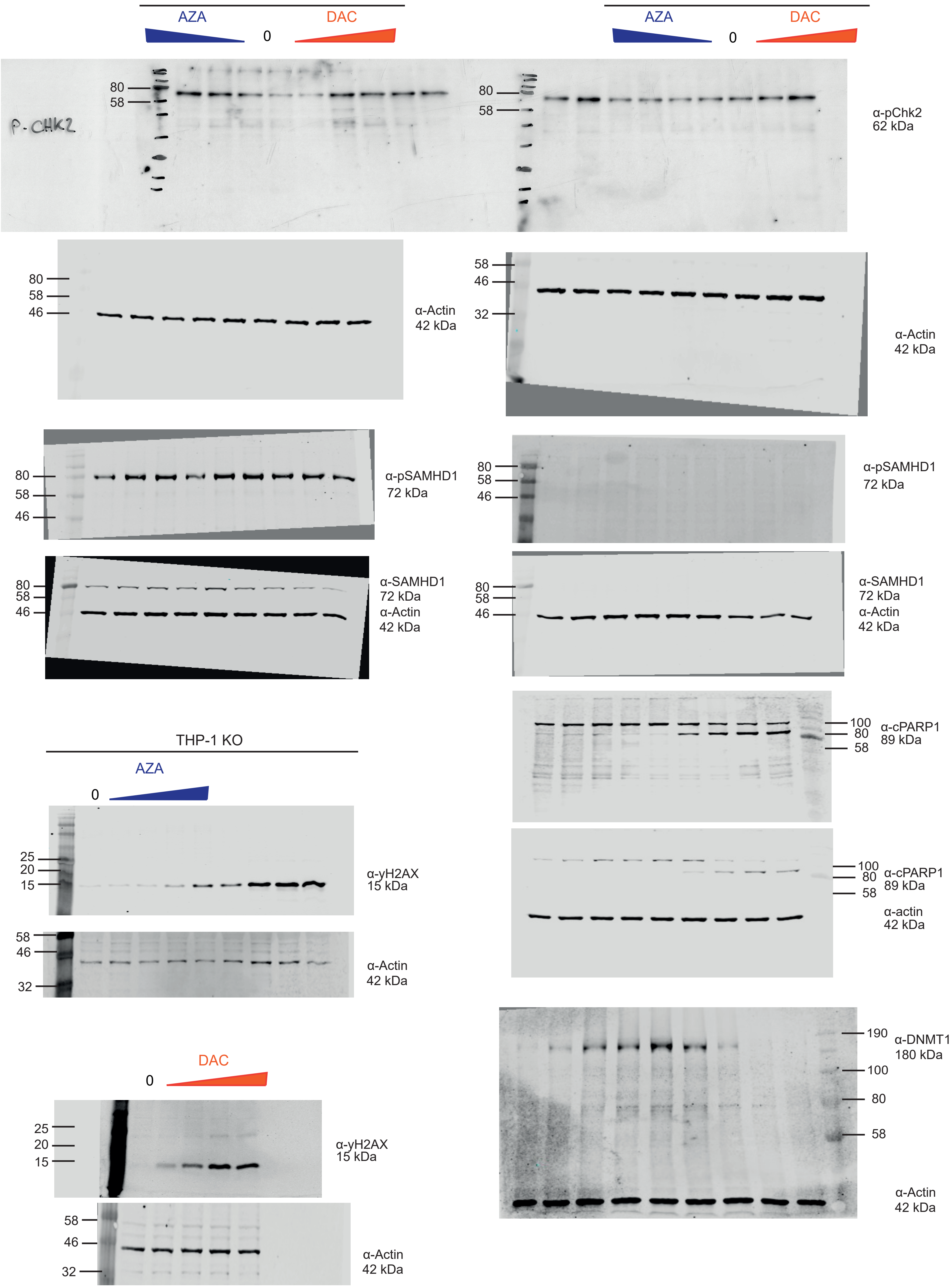

THP-1 Ctr.
AZA
DAC
0
THP-1 KO
AZA
DAC
0
P-CHK2
80
58
α-pChk2
62 kDa
α-Actin
42 kDa
46
32
α-pSAMHD1
72 kDa
α-SAMHD1
72 kDa
α-Actin
42 kDa
THP-1 KO
AZA
0
25
20
15
α-yH2AX
15 kDa
α-Actin
42 kDa
100
α-cPARP1
89 kDa
α-actin
42 kDa
190
α-DNMT1
180 kDa
DAC
0

Figure 3e

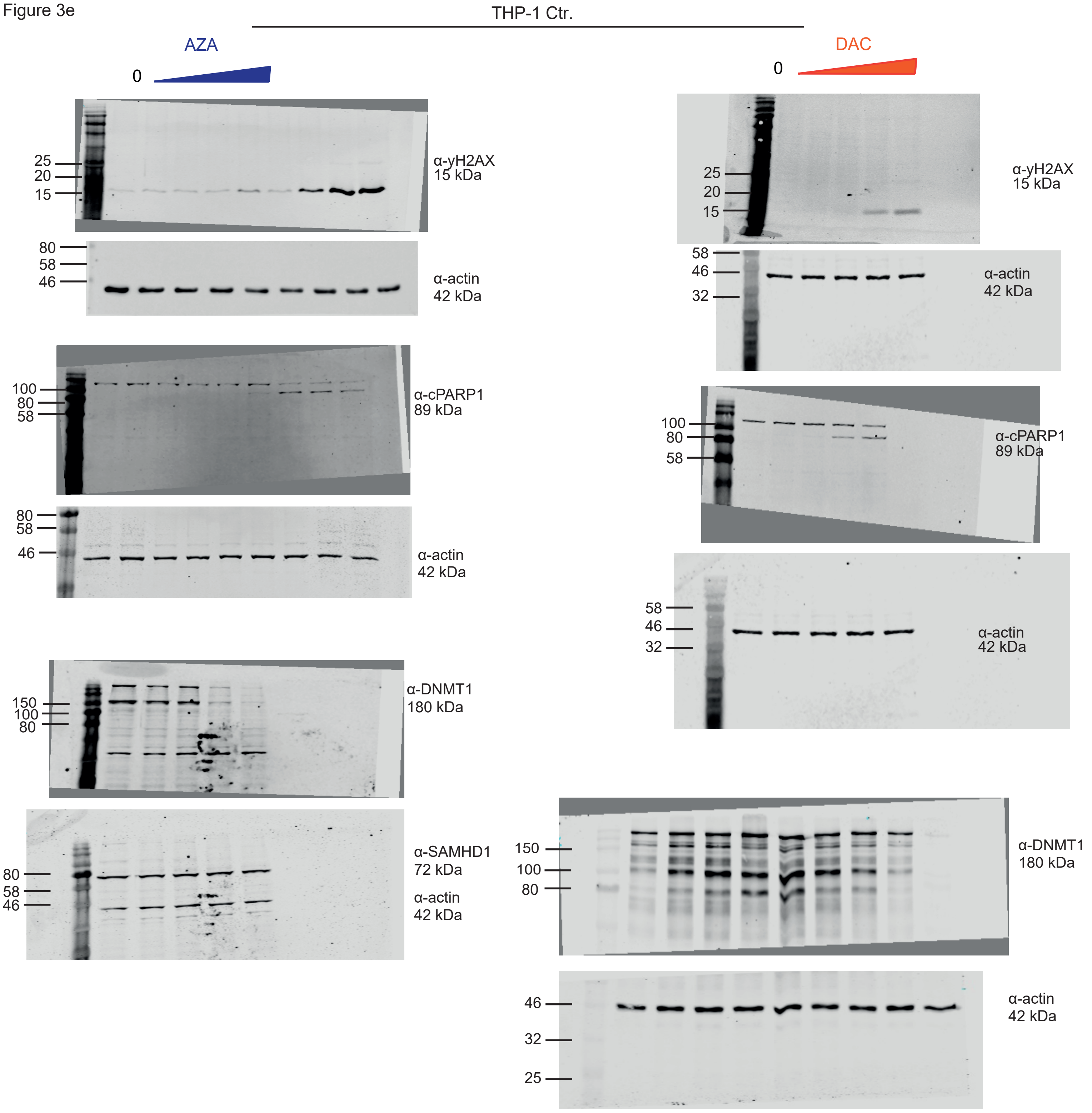

THP-1 Ctr.
AZA
0
DAC
0
25
20
15
α-yH2AX
15 kDa
80
58
46
α-actin
42 kDa
100
80
58
α-cPARP1
89 kDa
80
58
46
α-actin
42 kDa
150
100
80
α-DNMT1
180 kDa
80
58
46
α-SAMHD1
72 kDa
α-actin
42 kDa
25
20
15
α-yH2AX
15 kDa
58
46
32
α-actin
42 kDa
100
80
58
α-cPARP1
89 kDa
58
46
32
α-actin
42 kDa
150
100
80
α-DNMT1
180 kDa
46
32
25
α-actin
42 kDa

Figure 4a

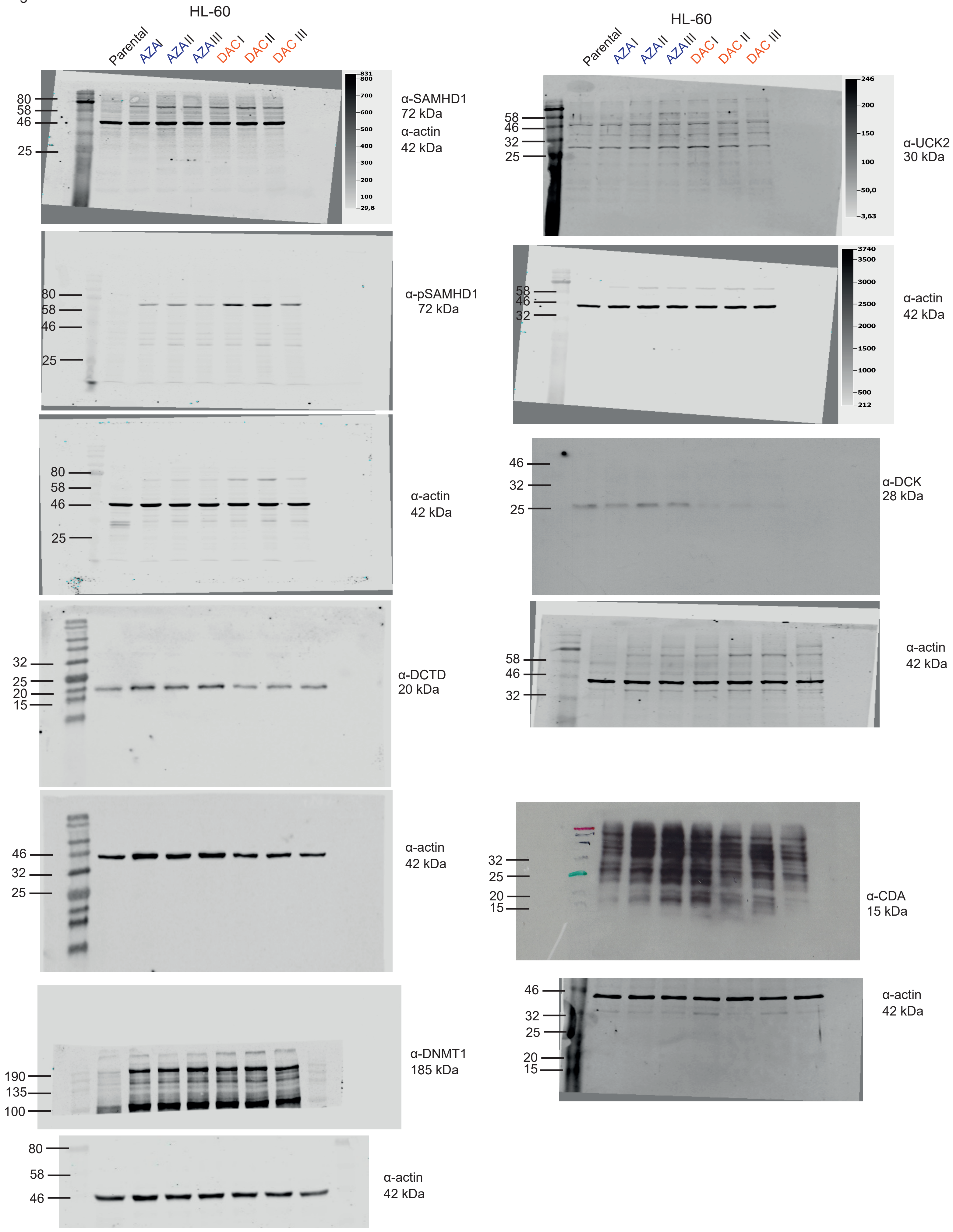
HL-60
Parental
AZA I
AZA II
AZA III
DAC I
DAC II
DAC III
α-SAMHD1
72 kDa
α-actin
42 kDa
α-pSAMHD1
72 kDa
α-actin
42 kDa
α-DCTD
20 kDa
α-actin
42 kDa
α-DNMT1
185 kDa
α-actin
42 kDa
HL-60
Parental
AZA I
AZA II
AZA III
DAC I
DAC II
DAC III
α-UCK2
30 kDa
α-actin
42 kDa
α-DCK
28 kDa
α-actin
42 kDa
α-CDA
15 kDa
α-actin
42 kDa

Figure 4a

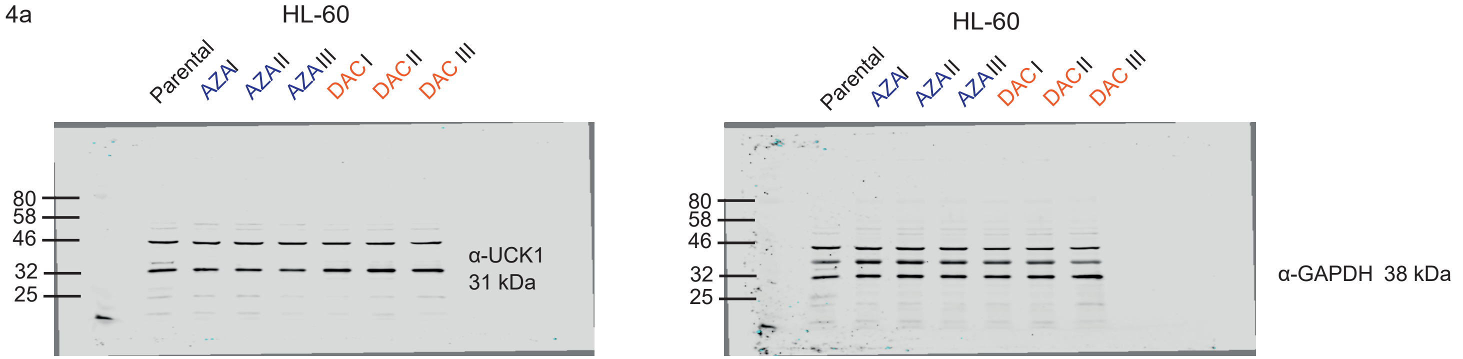

Figure 4d

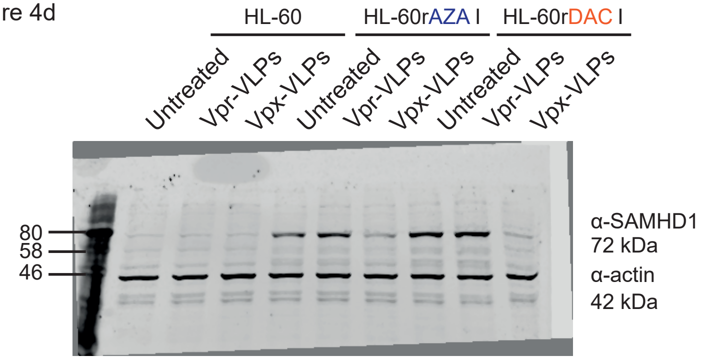

Figure 4g

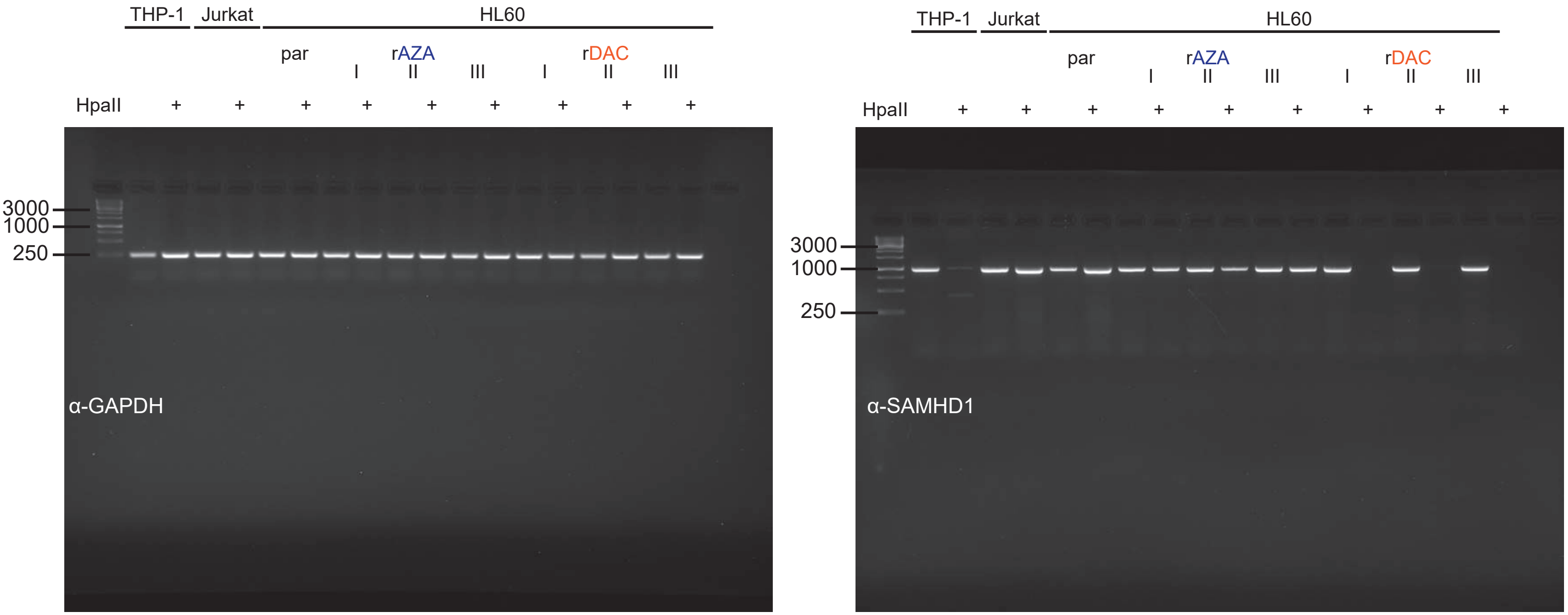

Figure 5

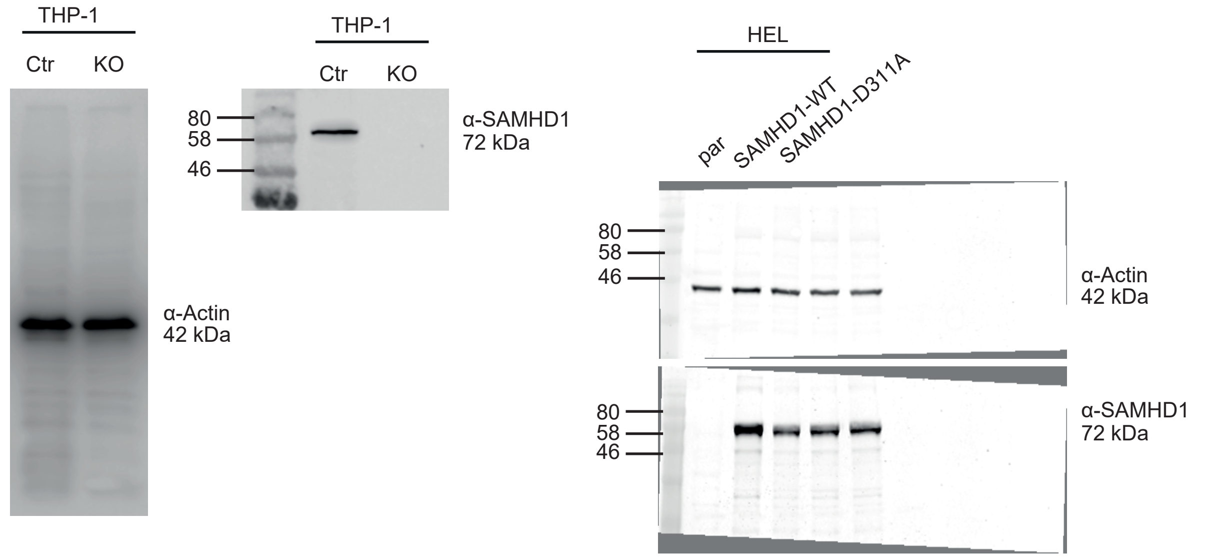

Figure 6e

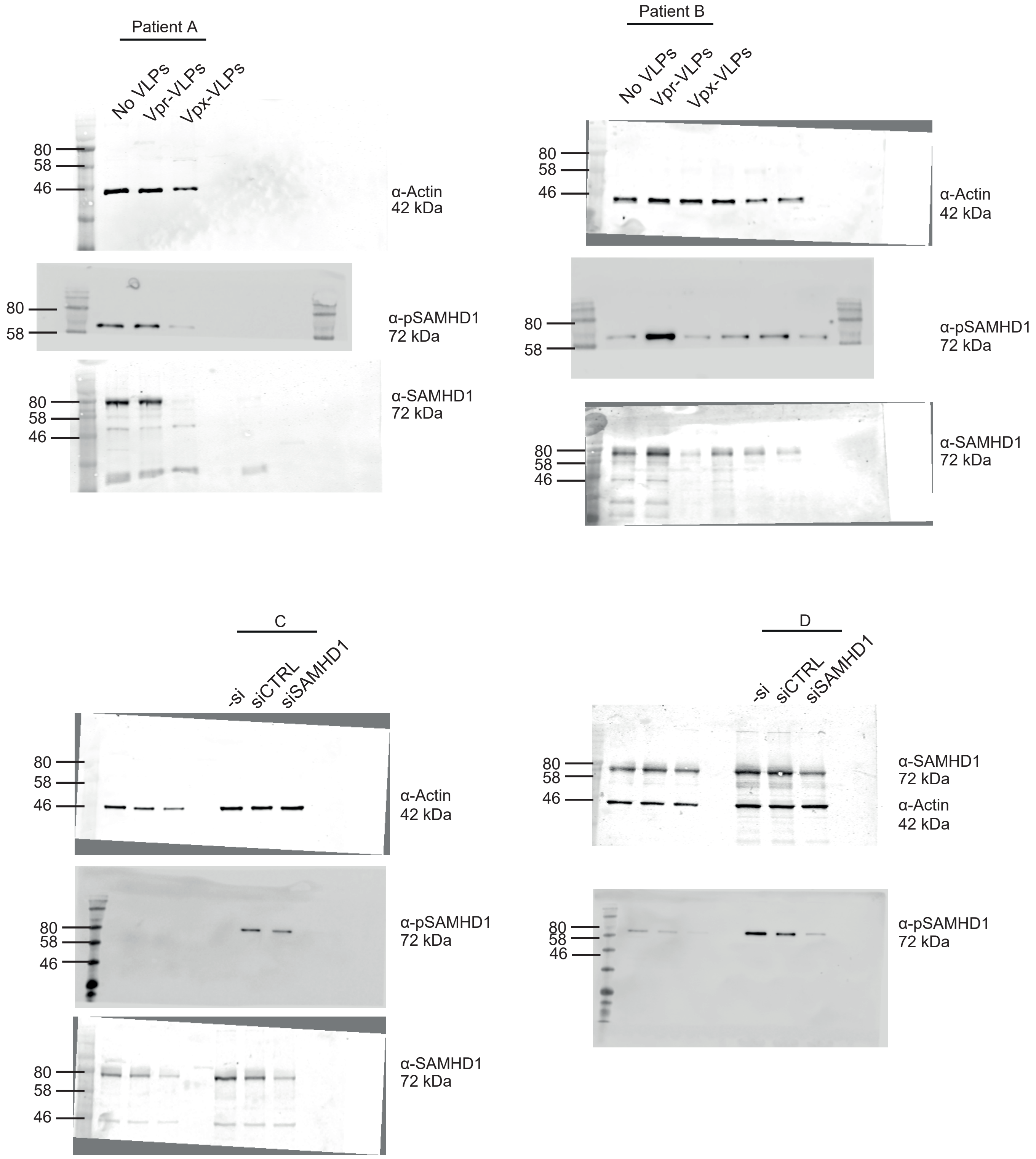

Supplementary Figure 9

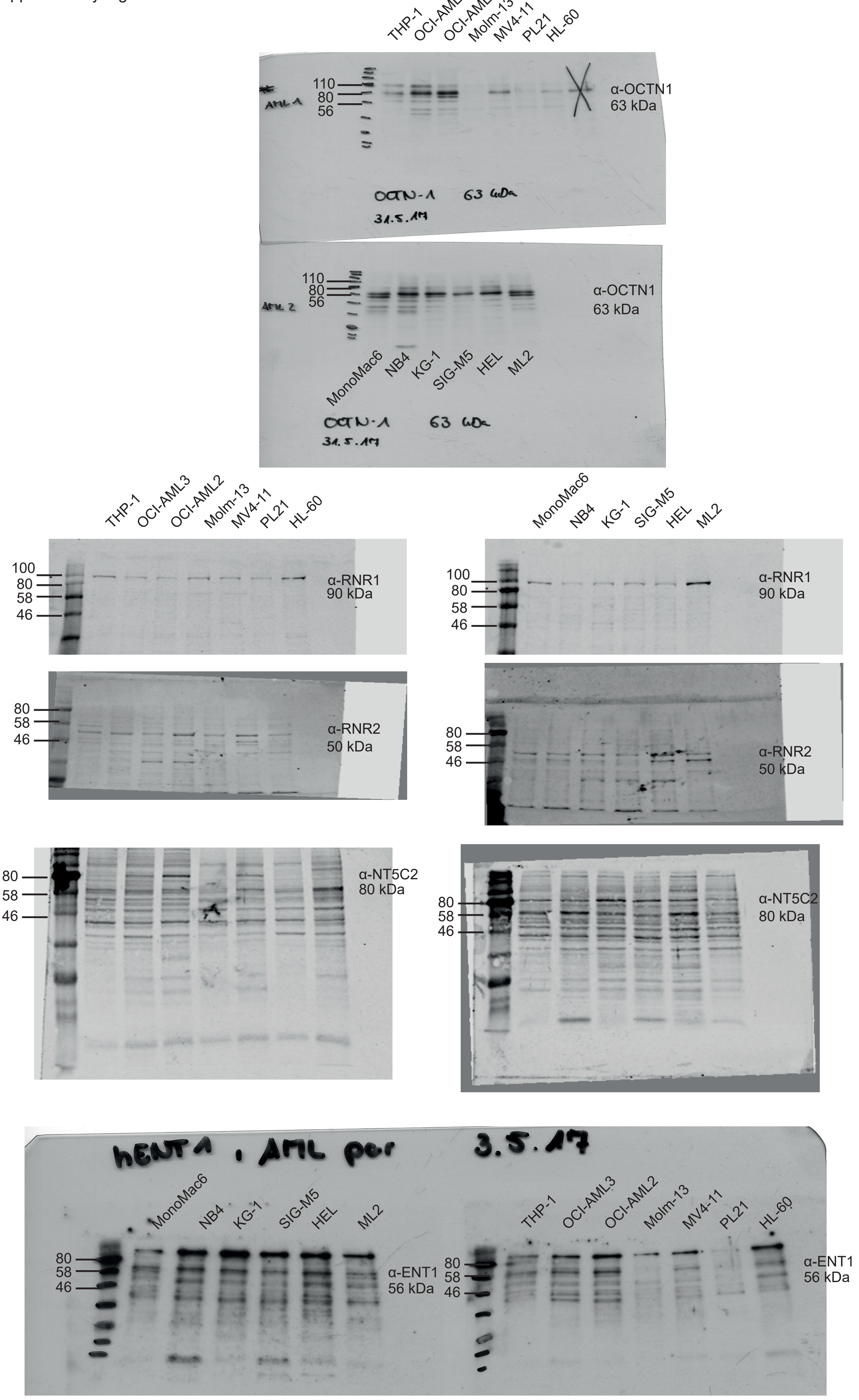

Supplementary Figure 9

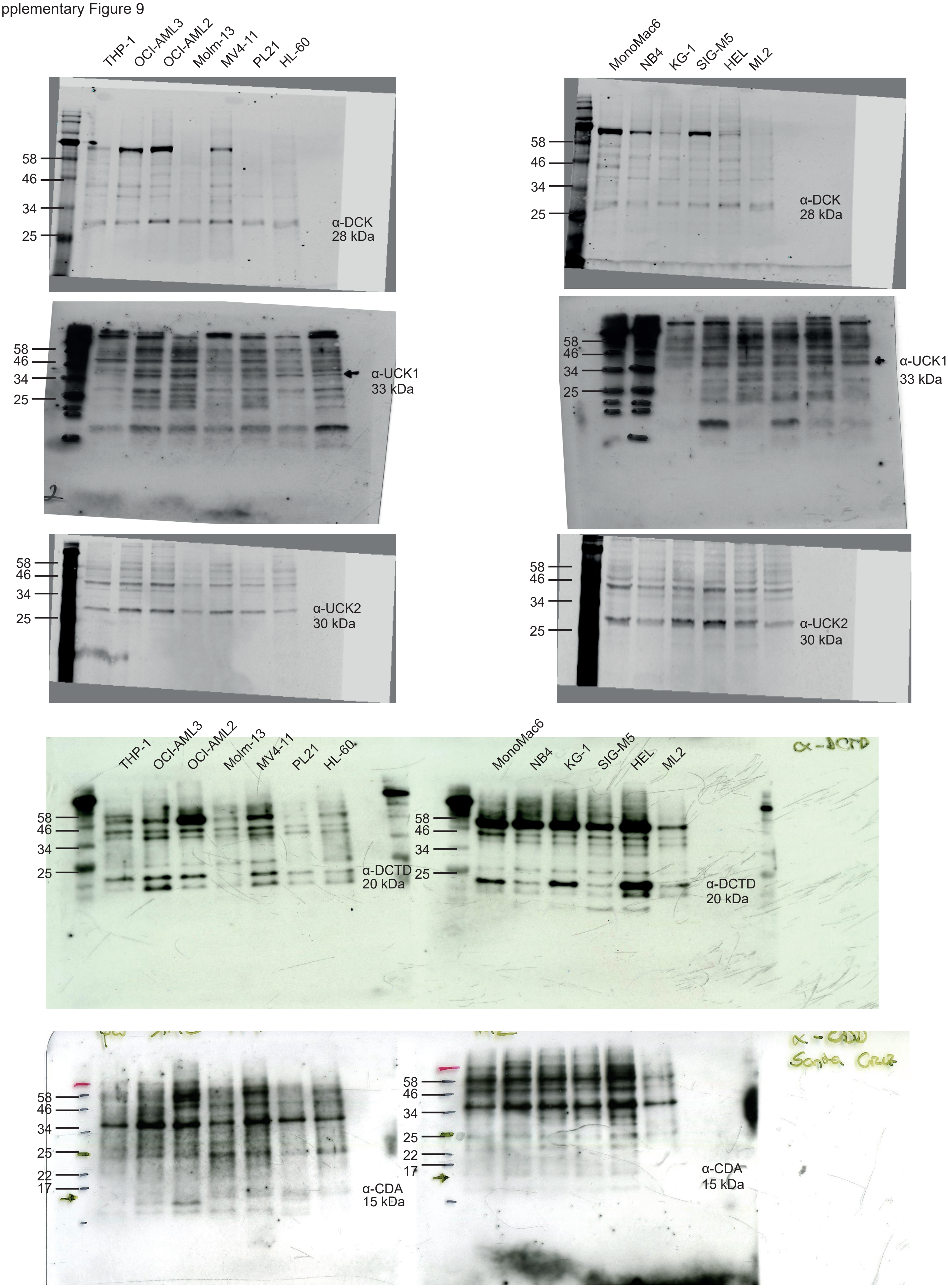

Supplementary Figure 13

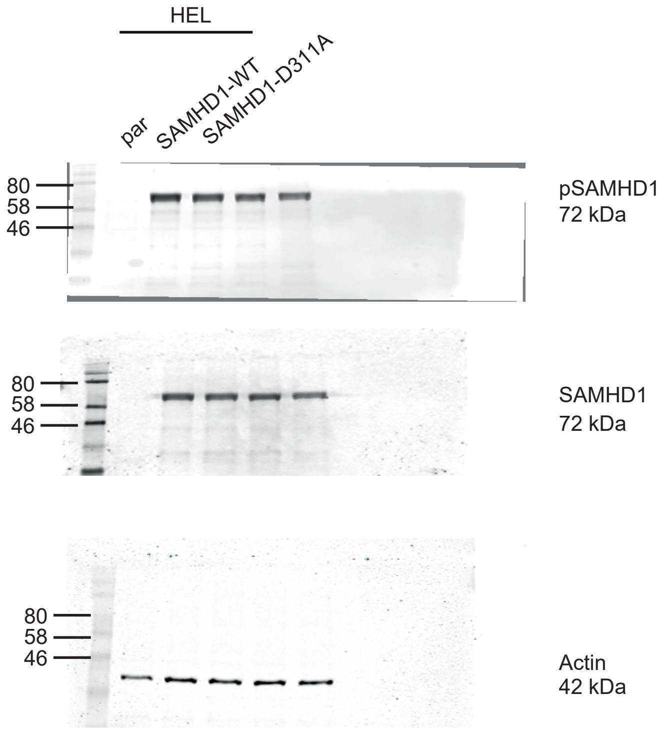

Supplementary Figure 18
48h -Etoposide

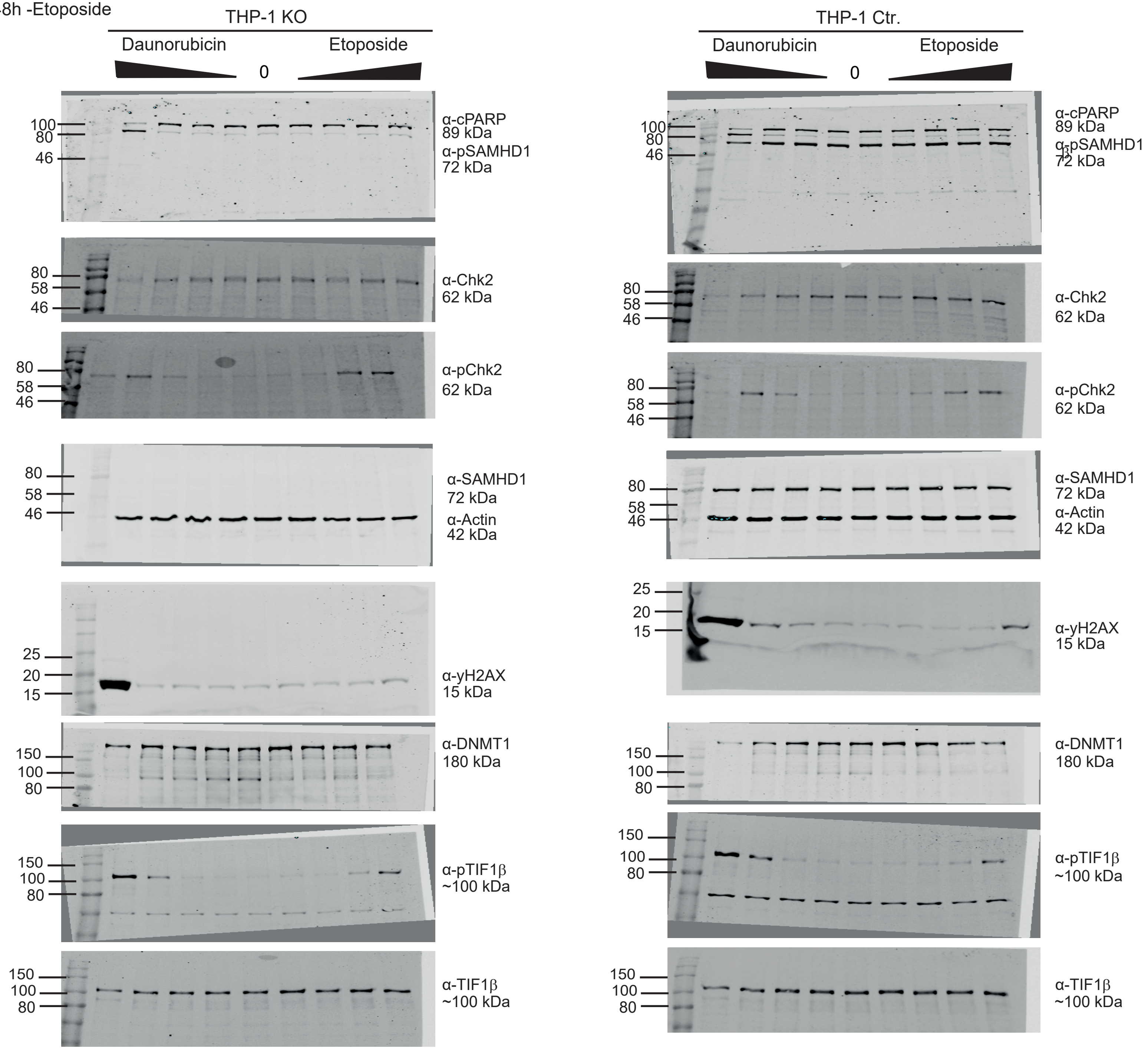

Supplementary Figure 19
72h - Dauno

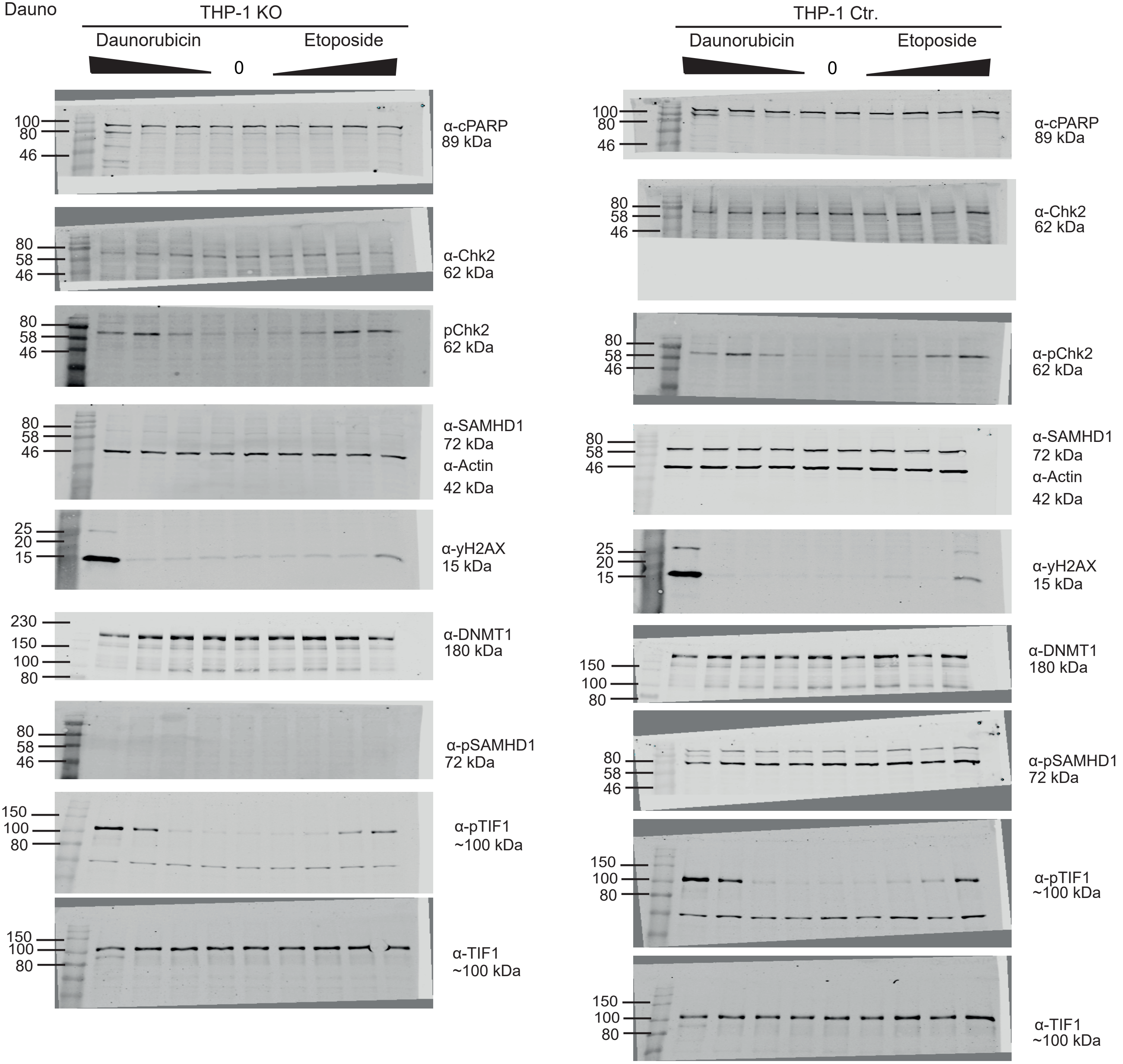

Supplementary Figure 22

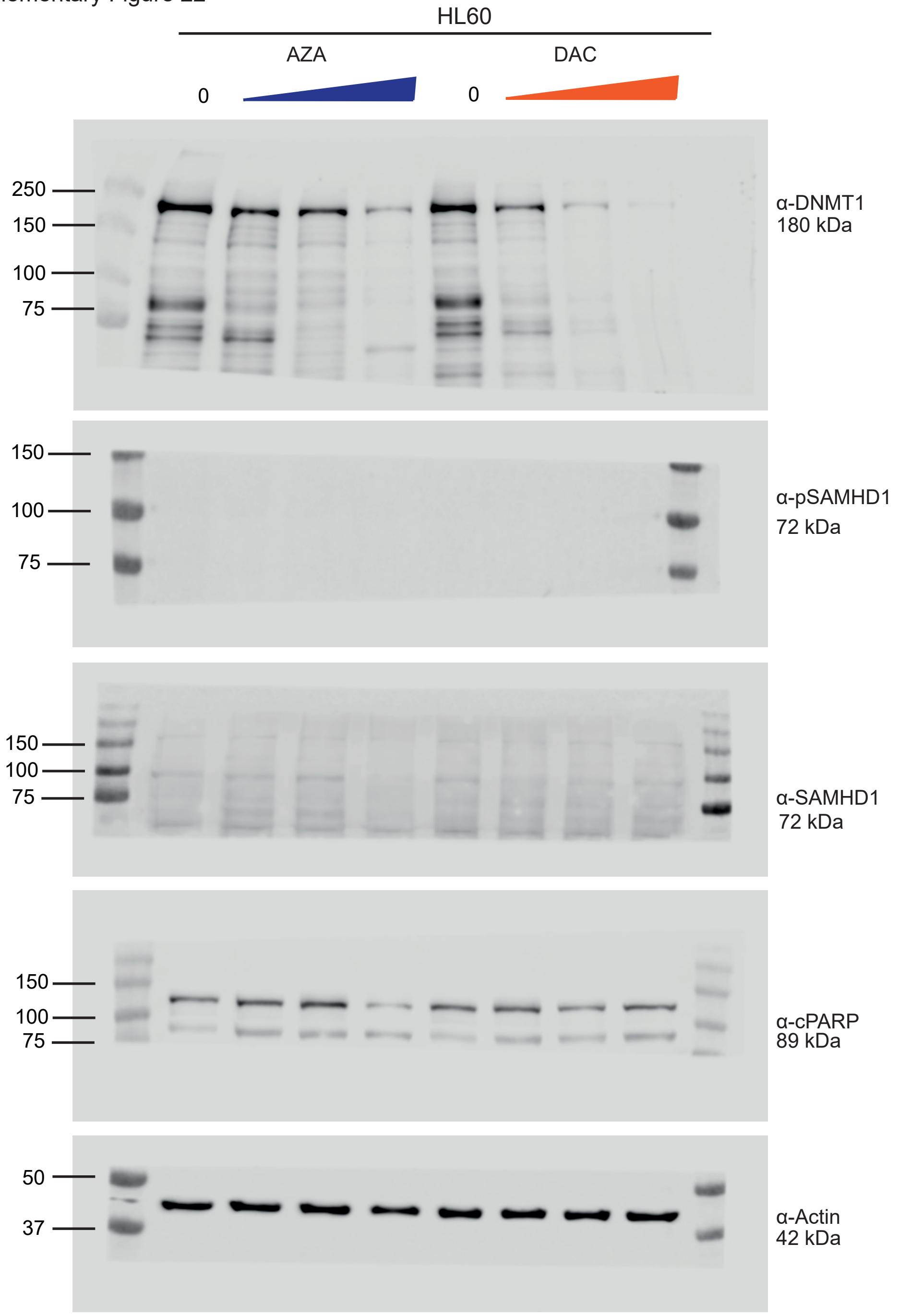

Supplement: Supplementary file 6 — Source Data [file 41467_2019_11413_MOESM6_ESM.zip › Source Data_raw Western Blots.pdf]
